# Total Synthesis of Mycinolide IV and Path‐Scouting for Aldgamycin N

**DOI:** 10.1002/anie.202016475

**Published:** 2021-02-26

**Authors:** Bart Herlé, Georg Späth, Lucas Schreyer, Alois Fürstner

**Affiliations:** ^1^ Max-Planck-Institut für Kohlenforschung 45470 Mülheim/Ruhr Germany

**Keywords:** antibiotics, asymmetric hydroformylation, macrolides, redox-isomerization, ruthenium, *trans*-hydrostannation

## Abstract

Proof‐of‐concept is provided that a large estate of 16‐membered macrolide antibiotics can be reached by a “unified” approach. The key building block was formed on scale by an asymmetric vinylogous Mukaiyama aldol reaction; its alkene terminus was then converted either into the corresponding methyl ketone by Wacker oxidation or into a chain‐extended aldehyde by catalyst‐controlled branch‐selective asymmetric hydroformylation. These transformations ultimately opened access to two structurally distinct series of macrolide targets. Notable late‐stage maneuvers comprise a rare example of a ruthenium‐catalyzed redox isomerization of an 1,3‐enyne‐5‐ol into a 1,3‐diene‐5‐one derivative, as well as the elaboration of a tertiary propargylic alcohol into an acyloin by *trans*‐hydrostannation/Chan‐Lam‐type coupling. Moreover, this case study illustrates the underutilized possibility of forging complex macrolactone rings by transesterification under essentially neutral conditions.

## Graphical abstract

Dozens of macrolide antibiotics are known that differ from each other only in the decoration of an otherwise highly conserved 16‐membered lactone core and in the lateral glycosylation pattern. A unified approach was explored that holds the promise of bringing numerous members of this type into reach from a single “eastern” building block.

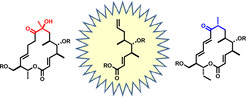

## Introduction

Actinobacteria in general and the genus *Streptomyces* sp. in particular rank amongst the most prolific sources of antibiotics that found their way into clinical use.[^1^, ^2^, ^3^] It has been noticed, however, that the rate of discovery of new antimicrobial agents from these sources is declining, thus making it necessary to explore strains collected at more remote places or in the (deep) sea, which have been studied less systematically in the past.[^1^, ^4^, ^5^] In this context, the isolation of several novel aldgamycin macrolides from the aquatic *Streptomycetes* strain HK‐2006‐1 is noteworthy, which exhibit significant and selective activity against *Staphylococcus aureus* 209P.[^6^, ^7^, ^8^]

**Figure 1:**
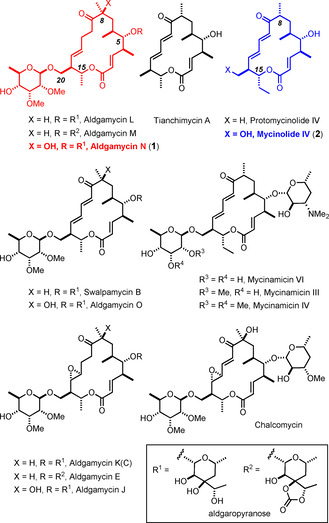
Selected members of the aldgamycin, mycinamicin, swalpamycin and chalcomycin macrolide antibiotics.

The aldgamycins are closely related to the macrolides of the mycinolide/mycinamicin,[^9^, ^10^] tianchimycin,^[11]^ swalpamycin,^[12]^ and chalcomycin^[13]^ series, all of which contain D‐mycinose whenever the primary C20‐OH group is glycosylated (Figure 1). The individual families are distinguished by subtle alterations in the glycosidation pattern at C5: specifically, all aldgamycins carry the eponymous aldgarose at this site, a highly unusual octopyranose, which may or may not feature a cyclic carbonate at the branching point; the other families have different sugars appended to this secondary hydroxy group or may not be glycosylated at all. Another modification in constitutional terms lies in the substituent branching off C15, in that the mycinolide/mycinamicin derivatives have a one‐carbon longer chain ending in an ethyl rather than the usual methyl group. Within a given family, the individual members differ from each other in the level of unsaturation/epoxidation of the “western sector” of the highly conserved 16‐membered macrolide core. An additional variation concerns the substitution pattern at C8, which can either be a tertiary alcohol or a simple methyl branch adjacent to the invariant carbonyl group at C9. Overall, this particular estate of macrocycles is an excellent example for how nature institutes diversity upon a conserved ichnography. Therefore it should potentially lend itself to an “integral synthesis” endeavor: provided one can formulate a modular assembly process, a fairly small number of building blocks should suffice to reach a significant subset of antibiotics of this type as well as non‐natural analogues for biological evaluation.^[14]^

Over this enticing outlook, however, one must not forget the lessons learnt from previous studies in this field. Members of the mycinamicin family were targeted in the past.[^15^, ^16^, ^17^, ^18^, ^19^] This precedent shows the delicacy of these compounds and illustrates the numerous challenges to be met en route to this sensitive chemotype. These issues notwithstanding, it was hoped that a “collective” rather than “individual” approach is feasible.[^20^, ^21^] For proof‐of‐concept, aldgamycin N (**1**)^[6]^ and mycinolide IV (**2**)^[9]^ were chosen as initial targets, because they (i) stand for the two subsets characterized by the different oxygenation pattern at C8, (ii) feature different levels of unsaturation within the macrolide ring, and (iii) represent the families with either a C15‐Me or a C15‐Et branch. If these particular compounds can be made, permutations of the modules needed for their synthesis should bring many of their siblings into reach.

**Scheme 1:**
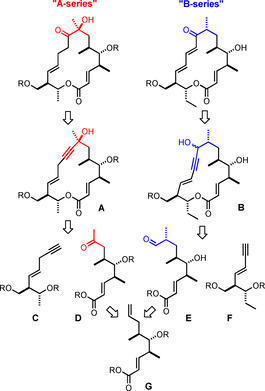
Layout of a potentially “collective” synthesis of the aldgamycin/mycinamicin macrolides.

To this end, the blueprint shown in Scheme 1 was pursued, which traces both series back to the very same unsaturated synthon **G**: Tsuji/Wacker oxidation^[22]^ followed by alkyny‐ lation of the resulting ketone **D** with the skipped enyne **C** was thought to open entry into the “A‐series”, since the triple bond in **A** can be seen as a carbonyl surrogate. Equally straightforward was the projected route to the “B‐series”, which capitalizes on a branch‐selective hydroformylation of the terminal alkene in **G**.[^23^, ^24^, ^25^, ^26^] However, the literature knows of surprisingly little precedent for (late‐stage) applications of this transformation in target‐oriented synthesis.[^27^, ^28^, ^29^] Provided this challenging step can be accomplished with the necessary level of regio‐ and stereoselectivity, alkynylation of the resulting aldehyde **E** with the conjugated 1,3‐enyne **F** followed by cyclization and redox‐isomerization of **B** might open the doorway, even though the exact order of events remains to be determined. In any case, the modular and divergent character of the overall synthesis plan and the need for a single building block **G** representing the eastern hemisphere of both series reduces the synthetic exertion and makes the approach potentially practical and scalable.

## Results and Discussion

**Scheme 2:**
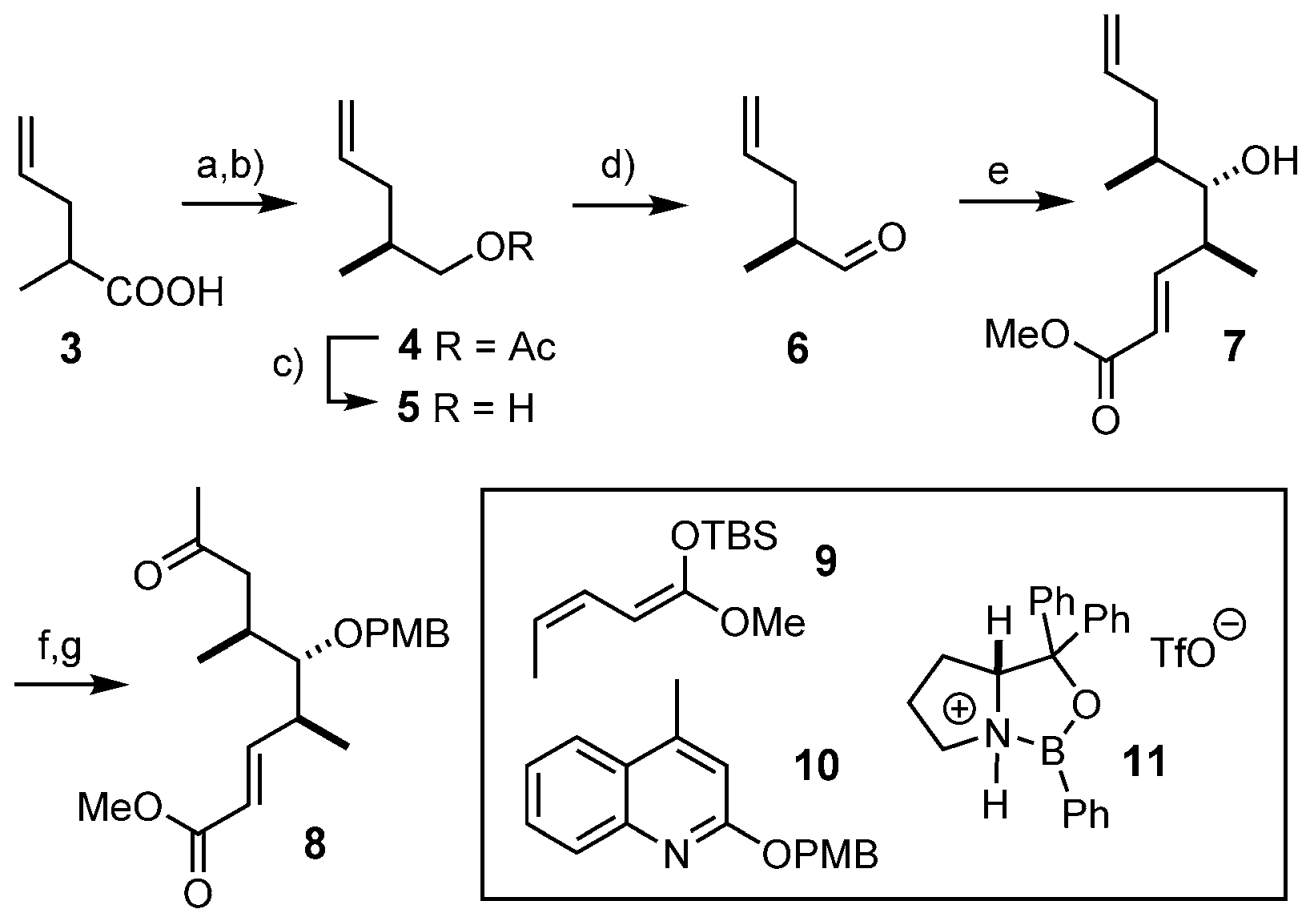
a) LiAlH_4_, THF, 91 %; b) vinyl acetate, Amano lipase (*Pseudomonas fluorescens*), THF, −20 °C, 40 %, 94 % *ee*; c) MeLi, Et_2_O, 75 %, −30 °C → RT; d) (COCl)_2_, DMSO, Et_3_N, CH_2_Cl_2_, −78 °C → RT; e) **9**, **11** (50 mol %), *i*PrOH, CH_2_Cl_2_, −78 °C, 69 % (over two steps, dr=89:11, C6‐epimers) [10 g scale]; f) **10**, TfOH cat., CH_2_Cl_2_, −20 °C → RT, 68 %; g) O_2_, PdCl_2_ (20 mol %), CuCl, THF, H_2_O, 88 %; PMB=*p*‐methoxybenzyl; TBS=*tert*‐butyldimethylsilyl; Tf=trifluoromethanesulfonyl.

A vinylogous asymmetric Mukaiyama aldol reaction was deemed the ideal entry point into the preparation of the common eastern fragment (Scheme 2).[^30^, ^31^] Rather than pursuing an auxiliary‐based approach for the preparation of the required aldehyde **6**, we opted for kinetic resolution. To this end, cheap **3** was reduced and the resulting alcohol reacted with vinyl acetate in the presence of *Pseudomonas fluorescens* lipase to give the corresponding acetate **4** with 94 % *ee* on >20 g scale (40 % yield of possible 50 %).^[32]^ Because of the volatility of the derived alcohol **5**, the deacetylation was best performed with MeLi in Et_2_O, as the lithium salts are easy to remove by aqueous work‐up and the ethereal solvent can be evaporated without undue loss of material. Subsequent Swern oxidation furnished aldehyde **6** without noticeable isomerization; the crude product was used in the subsequent vinylogous Mukaiyama aldol reaction to set the *syn*/*anti*‐configured stereotriade. At the outset we faced massive reproducibility issues, which could ultimately be traced back to the catalyst preparation. Gratifyingly, it was found that samples of complex **11** prepared in a separate step by condensation of diphenylprolinol and phenylboronic acid^[33]^ (rather than from PhBCl_2_)^[30]^ in a Dean–Stark trap followed by activation of the resulting oxazaborolidine with TfOH at low temperature led to good results, consistently furnishing compound **7** in 69 % yield (over two steps, dr=89:11, C6‐epimers, >10 g scale).[^34^, ^35^]

With access to decagram quantities of this key building block in only five operations, a solid basis was reached from which the project could branch out toward the two different product series. To this end, **7** was PMB‐protected; only the quinolone ether **10** worked well,^[36]^ whereas more traditional methods gave complex mixtures. The subsequent Tsuji/Wacker oxidation with catalytic PdCl_2_, CuCl as co‐catalyst, and oxygen as the terminal oxidant furnished the required methyl ketone **8** in high yield.^[22]^

**Scheme 3:**
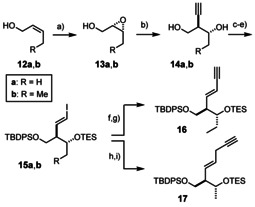
a) Cumene hydroperoxide, Ti(O*i*Pr)_4_, L‐diisopropyl tartrate, CH_2_Cl_2_, −20 °C, 54 % (**13 a**, 92 % *ee*), 73 % (**13 b**, 87 % *ee*); b) (i) [HC≡CLi]⋅eda, THF, 0 °C → RT; (ii) NaIO_4_, CH_2_Cl_2_/H_2_O, 42 % (**14 a**), 29 % (**14 b**, 97 % *ee* after recrystallization); c) TBDPSCl, imidazole, CH_2_Cl_2_, 83 % (R=H); d) TESOTf, 2,6‐lutidine, CH_2_Cl_2_, 0 °C → RT, 96 % (R=H), 91 % (R=Me, over both steps); e) (i) Cp_2_ZrCl_2_, Dibal‐H, THF, 0 °C → RT; (ii) I_2_, 2,6‐lutidine, THF, −78 °C, 65 % (**15 a**), 74 % (**15 b**); f) **15 b**, TMSC≡CH, [(PPh_3_)_2_PdCl_2_] (2.5 mol %), CuI, Et_3_N; g) K_2_CO_3_, MeOH, THF, 96 % (over two steps); h) TMSC≡CMe, *n*BuLi, THF, −78 °C, then **15 a**, CuI, DMAP, 0 °C → RT; i) K_2_CO_3_, MeOH, THF, 84 % (over two steps); Cp=cyclopentadienyl; Dibal‐H=diisobutylaluminum hydride; DMAP=4‐dimethylamino‐pyridine; eda=ethylene‐ 1,2‐diamine; TBDPS=*tert*‐butyldiphenylsilyl; TES=triethylsilyl; TMS=trimethylsilyl.

The necessary alkyne modules of type **C** and **F** could also be accessed by a uniform strategy (Scheme 3). Thus, Sharpless epoxidation of the homologous *Z*‐alkenes **12 a**,**b** followed by opening of the resulting oxirane derivatives **13 a**,**b** with lithium acetylide ethylenediamine complex proved practical:^[37]^ although the attack of the nucleophile is not overly regioselective, the undesired isomer—which is a 1,2‐ rather than 1,3‐diol—is readily discarded by an oxidative work‐up with NaIO_4_.^[37]^ After appropriate differential protection, the terminal alkyne was subjected to hydrozirconation/iodination;^[38]^ the procedure had to be modified in that 2,6‐lutidine was introduced prior to the addition of iodine to avoid cleavage of the TES‐ether. Subsequent Sonogashira coupling^[39]^ of **15 b** with trimethylsilylacetylene followed by selective cleavage of the C−Si bond gave the 1,3‐enyne **16** as required for the assembly of the “B‐series”. Equally facile was the elaboration of **15 a** into **17** by a copper‐mediated coupling with lithiated trimethylsilylpropyne;^[40]^ importantly, no trace of allene was observed in the crude mixture under these conditions nor after desilylation with K_2_CO_3_/MeOH.

**Scheme 4:**
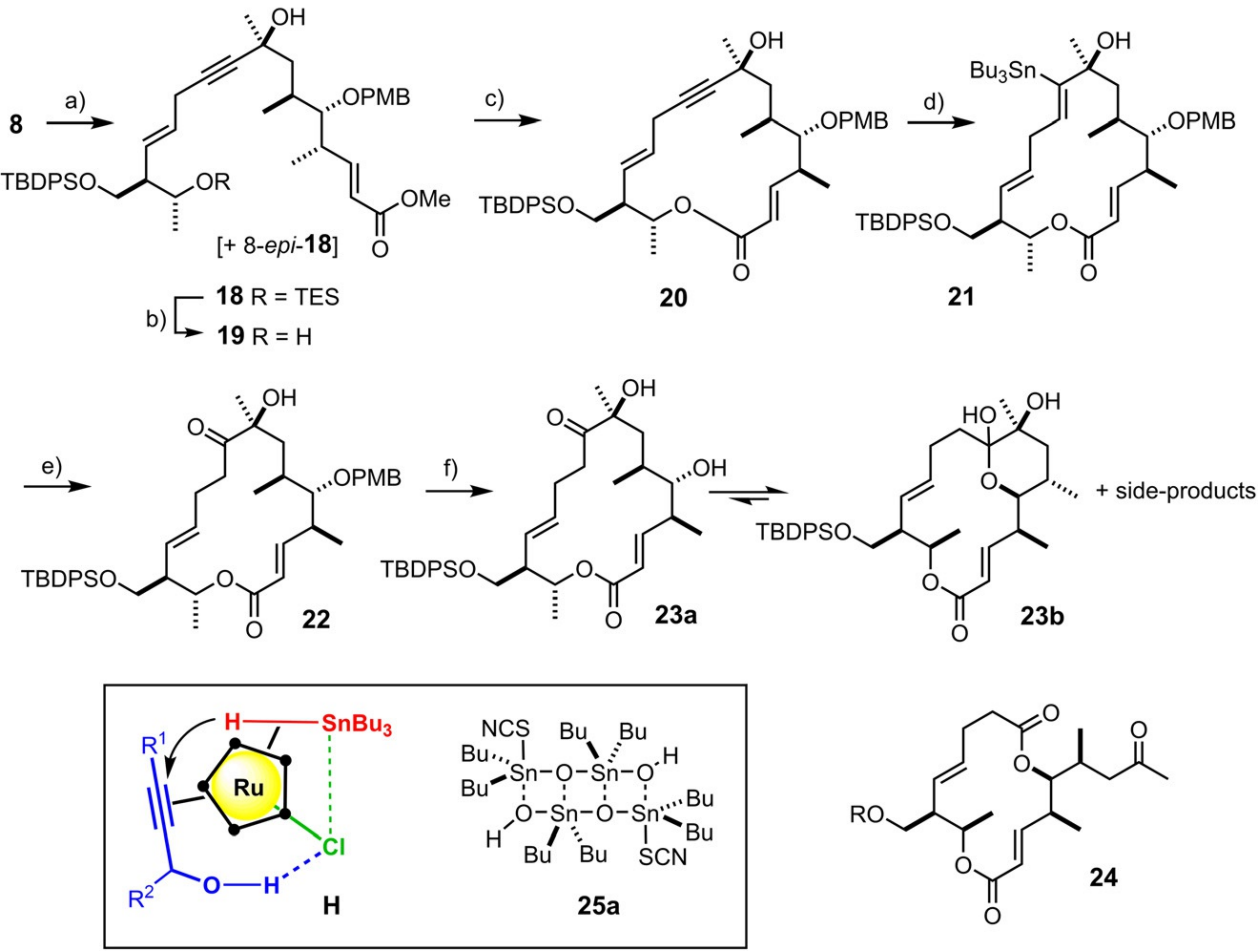
a) **17**, *n*BuLi, LaCl_3_⋅2LiCl, THF, −78 °C, 83 % (dr ≈1:1); b) PPTS, EtOH, 0 °C, 89 %; c) **25 a**, toluene, reflux, 68 %; d) [Cp*RuCl]_4_ (12 mol %), Bu_3_SnH, CH_2_Cl_2_, 72 %; e) [Cu(tfa)_2_]⋅H_2_O, DMAP (40 mol %), DMSO, 45 °C, 83 %; f) DDQ, CH_2_Cl_2_, H_2_O, see Text; Cp*=pentamethylcyclopentadienyl; DDQ=2,3‐dichloro‐5,6‐dicyano‐1,4‐benzoquinone; PPTS=pyridinium *p*‐toluenesulfonate; tfa=trifluoroacetate.

The skipped enyne **17** was deprotonated with *n*BuLi and the resulting lithio‐acetylide added to ketone **8** in the presence of LaCl_3_⋅2 LiCl to reduce the basicity of the reagent (Scheme 4).[^41^, ^42^] Unsurprisingly perhaps, the remote stereocenters in **8** (dr≈90:10) had no significant impact on the stereochemical course of the reaction. Because the two isomers were separable at this stage,^[43]^ no effort was made to impose better control over the addition process;^[44]^ rather, we were pressing forward to check the feasibility of the subsequent key steps en route to aldgamycin N (**1**) and its cousins of the “A‐series”. Whereas the selective deprotection of the TES‐ether of adduct **18** proceeded smoothly in acidic medium under carefully controlled conditions without damaging the acid‐sensitive tertiary alcohol, all attempts to cleave the methyl ester of **19** and release the *seco*‐acid in readiness for macrolactonization were met with poor yields or even complete failure.^[45]^ Rather than opting for a re‐launch of the project with a more orthogonal ester, we explored the possibility of forging the large ring by transesterification. Gratifyingly, stannoxane **25 a** proved adequate in that it allowed lactone **20** to be formed in 68 % yield on a decent scale (>400 mg, single largest batch).^[46]^ The isomeric addition product 8‐*epi*‐**18** was processed analogously to the corresponding epimeric lactone (see the SI); it was at this stage that the configuration of the C8‐stereocenter could be tentatively assigned, which was later confirmed by the total synthesis of aldgamycin N.^[47]^ In the end, this transesterification saved a step in the longest linear sequence as it rendered the formation of the *seco*‐acid obsolete. It is also notable that this example seems to be only the second successful application of this methodology to the synthesis of a macrolide natural product.[^48^, ^49^, ^50^, ^51^]

With the macrocyclic frame closed, we faced the challenge of transforming the propargylic entity of **20** into the acyloin motif characteristic of aldgamycin N (**1**) by regioselective hydration of the triple bond at the *more* hindered site. This goal was reached by resorting to a method previously developed in our laboratory,^[52]^ which was slightly modified and further improved for this particular application. Specifically, **20** was subjected to a ruthenium catalyzed *trans*‐hydrostannation, because this reaction faithfully delivers the ‐SnBu_3_ moiety to the position proximal to the ‐OH substituent.[^53^, ^54^] This regioselective outcome is rooted in a highly ordered transition state, in which the polarized [Ru−Cl] unit of the catalyst [Cp*RuCl] locks the substrate in place by interligand hydrogen bonding; at the same time, the chloride ligand steers the incoming stannane as shown in **H**.[^54^, ^55^] As expected, this directing effect was also operative in the present case in that alkenylstannane **21** was formed in good yield as a single regio‐ and stereoisomer. This compound was then subjected to a Chan‐Lam‐type coupling: rather than using Cu(OAc)_2_ in DMSO/Et_3_N as previously described,^[52]^ we resorted to Cu(tfa)_2_ in DMSO in the presence of catalytic amounts of DMAP. Under these conditions, the reaction proceeded under milder conditions and delivered the unprotected acyloin **22** right away instead of the corresponding acetate derivative that is generated when Cu(OAc)_2_ is used as the reagent.^[52]^

At this stage, the completion of the total synthesis of aldgamycin N seemed just a matter of routine protecting group manipulations and appropriate glycosidation reactions. This optimistic assessment, however, was premature: although cleavage of the PMB‐group per se worked well, the released C5‐OH group invariably engaged the ketone at C9 in transannular lactol formation; on top, the resulting hemiketal **23 b** proved sensitive, resulting in partial decomposition. Although the reasons for this instability were not investigated in any great detail, we noticed in parallel attempts using globally deprotected samples of the aglycone the formation of appreciable amounts (≈20 %) of the ring‐contracted diolide **24** (R=H), likely formed by spontaneous oxidative diol cleavage. This process is presumably also one of the ways by which **23 b** degrades to compounds of type **24** (R=TBDPS) and further downstream products. Attempted elaboration of this mixture into the target compound **1** was to no avail.^[56]^

Although this unforeseen transannular interference stopped this initial foray towards aldgamycin N, the obtained results clearly proved the viability of all key transformations meant to provide access to the “A‐series”. The only significant modification to be implemented concerns the timing of the events: the aldgarose unit must be introduced at an earlier stage rather than in the penultimate step, as originally planned. The price to pay is the need to carry this precious monosaccharide through a number of steps along the longest linear sequence; the accompanying paper describes how this challenge has been met and an efficient synthesis of **1** been accomplished.^[47]^

**Scheme 5:**
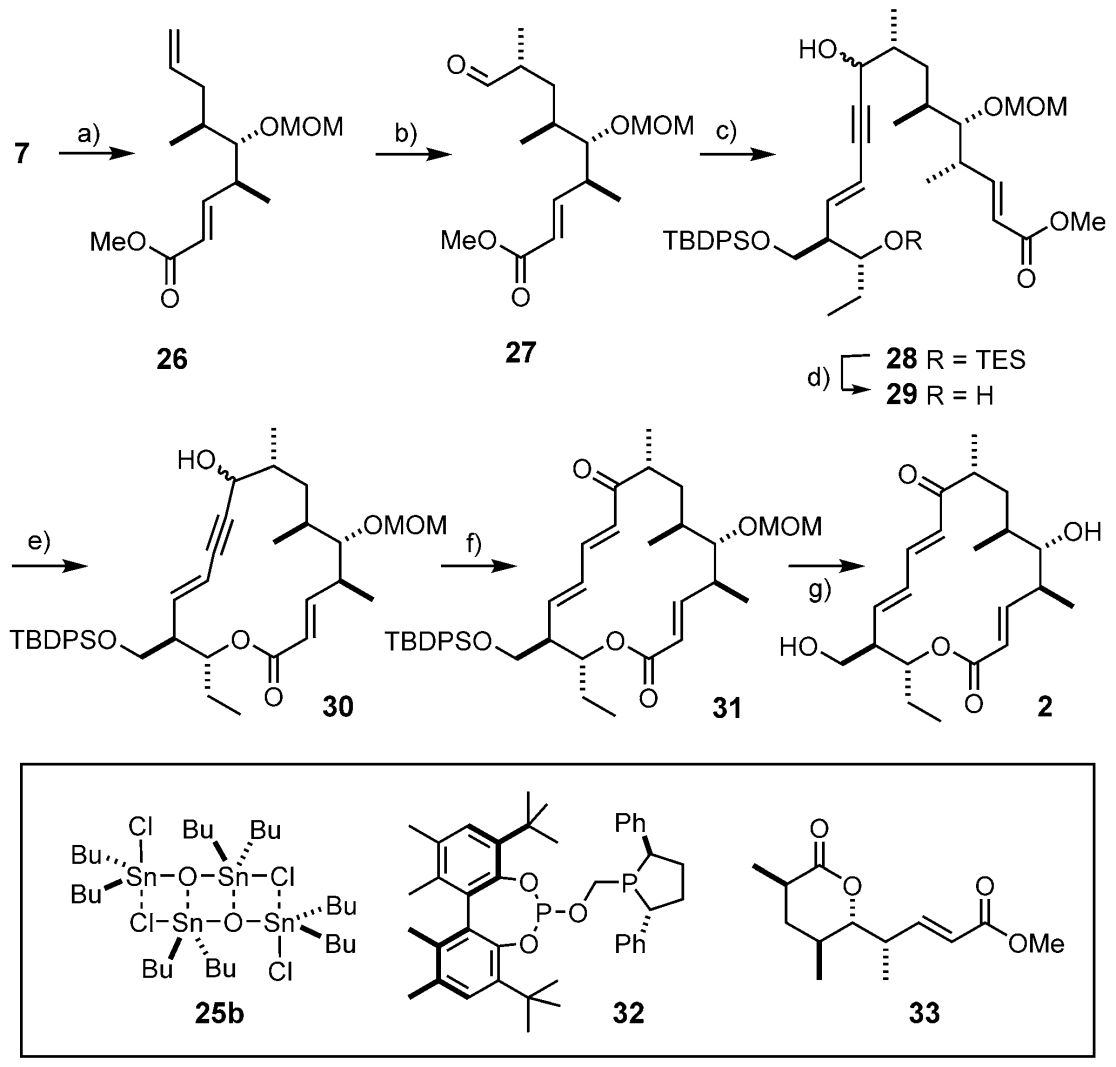
a) CH_2_(OMe)_2_, P_4_O_10_, CH_2_Cl_2_, 87 %; b) [Rh(acac)(CO)_2_] (3.4 mol %), **32** (4.2 mol %), H_2_/CO (1:1, 15 bar), C_6_F_6_, 30±1 °C, 60 % (dr=83: 17 (Σ of all other isomers)); c) **16**, *n*BuLi, THF, −78 °C, 56–65 % (dr=1.4:1); d) camphor‐10‐sulfonic acid (5 mol %), MeOH, CH_2_Cl_2_, −20 °C, 86 %; e) **25 b**, chlorobenzene, reflux, 32–37 %; f) [CpRu(MeCN)_3_]BF_4_ (50 mol %), PhPCy_2_ (50 mol %), THF, reflux, 65 %; g) aq. HCl (3 M), MeOH, 40 °C, 74 %; acac=acetylacetonato.

From a purely strategic perspective, however, it was deemed essential to first validate the projected route to the “B‐series”, which constitutes an equally integral part of the proposed “unified” approach shown in Scheme 1. It was arguably most important to check whether the common building block **7** is amenable to a catalyst‐controlled branch‐selective asymmetric hydroformylation or not. Although the C5‐OH group of this substrate suggested that recourse should be taken to a covalently bound directing group, which is a well‐established tactic for this purpose,^[23]^ the need to introduce and later remove such an auxiliary in separate operations was deemed far from ideal. In the end, it turned out to be unnecessary to resort to such a maneuver because the MOM‐derivative **26** (dr=87:13) succumbed to the desired transformation in the presence of a catalyst that had to be pre‐formed from [Rh(acac)(CO)_2_] and (*R*_ax_,*R*,*R*)‐BOBPhos (**32**) prior to addition of the substrate (Scheme 5).[^26^, ^57^] Further optimization of the reaction showed that *strictest* temperature control during the actual hydroformylation step was quintessential for success:^[58]^ when performed at 30±1 °C in hexafluorobenzene as the preferred solvent, gram scale experiments furnished the desired aldehyde **27** in respectable 60 % yield with an isomer ratio of 83:17 (sum of all undesired isomers). When assessing this result, one has to consider that the chosen substrate **7** had not been isomerically pure but had a dr of 89:11, as set by the Mukaiyama aldol reaction; the methyl‐branched chiral center itself was hence formed under the aegis of the catalyst with a dr=96:4. Control experiments using the enantiomeric BOBPhos ligand proved that the induction results from catalyst‐ rather than substrate‐control; the absolute configuration of the newly formed stereocenter was proven by transformation of **27** into the literature‐known derivative **33** ^[15a]^ and careful comparison of the NMR data (see the Supporting Information).

Addition of the lithiated enyne **16** to freshly prepared aldehyde **27** ^[59]^ gave alcohol **28** as an inconsequential mixture of isomers, which were treated with camphorsulfonic acid in MeOH/CH_2_Cl_2_ at −20 °C to deprotect the TES ether selectively. Once again, attempted formation of the *seco*‐acid by cleavage of the methyl ester in **29** via chemical or enzymatic means was to no avail, but macrolactonization by transesterification with the aid of stannoxane **25 b** proved again viable, although the reaction was very slow even in refluxing chlorobenzene (rather than toluene) and the yield of **30** significantly lower than in case of **20**.[^60^, ^61^] The difference is ascribed to the higher rigidity imposed onto the incipient ring by the conjugated *E*‐configured enyne relative to the skipped enyne subunit in **30** that contains a rotatable bond in between the stiff substructures.

At this stage, the only remaining key step to be accomplished was the reorganization of the π‐system of the propargylic alcohol **30** to the corresponding dienone. Ruthenium‐catalyzed redox isomerizations of 1,3‐enyn‐5‐ol derivatives, however, are extremely scarce;^[62]^ moreover, the conjugated π‐systems present in the enyne substrate and the resulting diene product might disfavor or even impede the reaction as they are able to bind tightly to the active [CpRu] fragment.^[54]^ Despite these concerns, the conversion of **30** into **31** proceeded well under conditions previously optimized in our laboratory,^[63]^ even though a high catalyst loading was indeed necessary.^[43]^ In contrast to the original literature on ruthenium‐catalyzed cycloisomerization,[^62^, ^64^, ^65^] it proved beneficial to leave any acid co‐catalyst out; rather, addition of PhPCy_2_ to the [CpRu(MeCN)_3_]BF_4_ complex ensured that the reaction proceeded cleanly and in well reproducible yield.[^66^, ^67^] Cleavage of the protecting groups with aq. HCl in MeOH also worked well to give mycinolide IV (**2**) as a colorless solid material. The analytical and spectral data of our samples matched those of the authentic compound reported in the literature in every regard.[^9^, ^17a^]

## Conclusion

Needless to say that the two orthogonal protecting groups in **31** ensure that this compound can also be elaborated into the glycosylated siblings of **2**; this aspect is subject to ongoing work in our laboratory. At this point, however, the key strategic goal of this endeavor had essentially been attained in that a “unified” approach to the mycinamicin and the aldgamycin series was shown to be feasible. While the conquest of mycinolide IV (**2**) in only 12 steps (longest linear sequence) leaves no doubt that various natural and non‐natural compounds of the “B‐series” can be formed analogously in a “serial” manner, one might object that a *rigorous* proof for the complementary “A‐series” is yet missing. Although the newly scouted pathway envisaging an early glycosylation event seems perfectly viable, only the crossing of the finish line is what ultimately counts. The accompanying paper describes how this second task has been reached.

## Conflict of interest

The authors declare no conflict of interest.

## Supporting information

As a service to our authors and readers, this journal provides supporting information supplied by the authors. Such materials are peer reviewed and may be re‐organized for online delivery, but are not copy‐edited or typeset. Technical support issues arising from supporting information (other than missing files) should be addressed to the authors.

Supplementary
