# Supplementary material for: Total Synthesis of Mycinolide IV and Path‐Scouting for Aldgamycin N
Source: Angew Chem Int Ed Engl. 2021 Feb 26;60(14):7893–9. doi: 10.1002/anie.202016475 (PMC8048839; doi:10.1002/anie.202016475)
Supplement: Supplementary file 1 — Supplementary [file ANIE-60-7893-s001.pdf]

Angewandte Chemie
Eine Zeitschrift der Gesellschaft Deutscher Chemiker

Supporting Information

Total Synthesis of Mycinolide IV and Path-Scouting for Aldgamycin N

*Bart Herlé[+], Georg Späth[+], Lucas Schreyer, and Alois Fürstner**

anie_202016475_sm_miscellaneous_information.pdf

General.

Unless otherwise stated, all reactions were performed in oven-dried (80 °C) or flame-dried glassware in anhydrous solvents under argon, applying standard Schlenk techniques. Dry argon (>99.5%) was purchased from Air Liquide.

The following solvents were purified by distillation over the indicated drying agents and transferred under argon: tetrahydrofuran and diethyl ether (Mg/anthracene), dichloromethane (CaH_2), hexanes and toluene (Na/K), methanol (Mg, stored over 3 Å molecular sieves). Acetonitrile, dimethyl sulfoxide, dimethylformamide, pyridine and triethylamine were dried using an adsorption (molecular sieves) solvent purification system. Solvents were removed under reduced pressure below 40 °C using a rotary evaporator.

Commercial technical-grade cumene hydroperoxide (Aldrich, 80 % *w/w*) was used as received. Commercial titanium(IV) isopropoxide was distilled under reduced pressure and stored under argon at −20 °C. Trimethylsilylacetylene and hexafluorobenzene were distilled under argon before use. Commercial anhydrous dimethoxymethane was stored over activated 4 Å molecular sieves under argon. Chlorobenzene was dried and stored over activated 4 Å molecular sieves, and degassed by bubbling argon through it. Amano Lipase (*Pseudomonas fluorescens*) was purchased from Aldrich. Silyl ketene acetal **9**,[1] distannoxane **25a**,[2] distannoxane **25b**,[3] and (R_{ax},*R*,*R*)-BOBPhos (**32**)[4] were prepared according to literature procedures.

Thin layer chromatography (TLC) was performed on Macherey-Nagel precoated plates (POLYGRAM® SIL/UV254); the compounds were detected by UV light (254 nm) or heating of the plate with a heat gun after treatment with stain solutions comprising either potassium permanganate or phosphomolybdic acid. Flash chromatography was performed with VWR silica gel 60 (40 – 63 µm). Automated column chromatography was conducted on a Biotage® Isolera™ or a Biotage® Selekt instrument, using the chromatography cartridges indicated in the respective procedure. Diastereomeric ratios (d.r.) of intermediates were determined by ^{1}H NMR spectroscopy from the relative integrals of sufficiently separated, characteristic signals of the respective intermediate.

NMR spectra were recorded on Bruker AV 400, AV 500 or AVIII 600 spectrometers in the solvents indicated. The solvent signals were used as references, chemical shifts were converted to the TMS scale and reported as follows: chemical shift in ppm (multiplicity, coupling constant *J* in Hz, number of protons). Multiplets are designated by the following abbreviations: s for singlet, d for doublet, t for triplet, q for quartet, quint for quintet, m for complex pattern (multiplet); the abbreviation br indicates a broad signal. ^{13}C NMR spectra were recorded in {^{1}H}-decoupled mode. Melting points were determined using a Büchi B-540 apparatus. IR spectra were recorded on a Bruker Alpha Platinum ATR spectrometer at room

temperature. Mass spectra were recorded using the following instruments: MS (EI): Finnigan MAT 8200 (70 eV), ESI-MS: Bruker ESQ3000, accurate mass determinations: Bruker APEX III FT-MS (7 T magnet) or Finnigan MAT 95. GC-MS samples were processed on a Shimadzu GCMS-QP2010 Ultrainstrument. Specific optical rotatory power ($[\alpha]_D$) was measured with the A-Krüss Otronic Model P8000-t polarimeter at a wavelength of 589 nm. The values are given with respect to exact temperature, concentration (c/(10mg/mL)) and solvent.

Preparation of the Alkyne Fragments

(*Z*)-But-2-en-1-ol (12a). In a 500-mL three-necked round-bottomed flask under hydrogen atmosphere

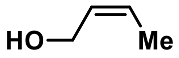

(two balloons), 2-butyn-1-ol (11 mL, 147 mmol) was added to a vigorously stirred suspension of $Pd/BaSO_4$ (10 % *w/w*, 1.07 g, 1.00 mmol) in diethyl ether (200 mL). The suspension was stirred under a hydrogen atmosphere for 44 h, until reaction monitoring by ^1H NMR indicated full consumption of the alkyne. For work-up, the flask was purged with argon and the suspension filtered through a pad of Celite®, which was rinsed with diethyl ether (5 × 15 mL). The combined filtrates were carefully evaporated (500 mbar, 36 °C) to give a yellowish liquid. Distillation at reduced pressure (5 cm Vigreux column; ~100 mbar, bp 60 – 65 °C) gave the title compound as a colorless liquid (5.6 g, 53% yield). **^1H NMR (400 MHz, $CDCl_3$):** δ 5.71 – 5.57 (m, 2H), 4.27 – 4.16 (m, 2H), 1.73 – 1.63 (m, 3H), 1.22 (br s, 1H). **$^{13}C\{^1H\}$ NMR (101 MHz, $CDCl_3$):** δ 129.4, 127.4, 58.4, 13.1.

((2*S*,3*R*)-3-Methyloxiran-2-yl)methanol (13a). A 1-L Schlenk flask with cooling jacket was charged

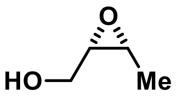

with powdered 4 Å molecular sieves (~6 g) and dichloromethane (320 mL). After the suspension had been cooled to −20 °C, titanium(IV) isopropoxide (5.0 mL, 17 mmol) and (+)-diisopropyl L-tartrate (4.2 mL, 20 mmol) were added and stirring was continued for 20 min at −20 °C. Cumene hydroperoxide (80% technical grade, 27 mL, 146 mmol) was added and the resulting mixture stirred for 30 min before a mixture of **12a** (6.04 g, 83.8 mmol) and powdered 4 Å molecular sieves (~1.5 g) in dichloromethane (40 mL) was introduced. Stirring was continued for another 14 h at −20 °C. After the addition of citric acid monohydrate (3.56 g) in diethyl ether/acetone (225 mL/25 mL), the mixture was warmed to room temperature and stirred for 30 min before the orange suspension was filtered through a short pad of Celite®, which was rinsed with dichloromethane (5 × 10 mL). The combined filtrates were concentrated under reduced pressure (150 mbar, 38 °C) and the residue was purified by flash chromatography (pentane/diethyl ether, 1:1) to give the title compound as a colorless liquid (3.98 g, 54% yield, 92% *ee*). $[\alpha]_D^{20}$ = −8.8 (*c* 1.2, $CHCl_3$). **^1H NMR (400 MHz, $CDCl_3$):** δ 3.90 – 3.80 (m, 1H), 3.75 – 3.65 (m, 1H), 3.19 – 3.11 (m, 2H), 1.75 – 1.69 (m, 1H), 1.33 (d, *J* = 5.7 Hz, 3H). **$^{13}C\{^1H\}$ NMR (101 MHz, $CDCl_3$):** δ 60.7, 56.7, 52.8, 13.4. The enantiomeric purity was determined by GC (see below).

The racemic sample was obtained by epoxidation of (*Z*)-2-buten-1-ol with 3-chloroperbenzoic acid. The analytical data are in agreement with those reported in the literature.[5]

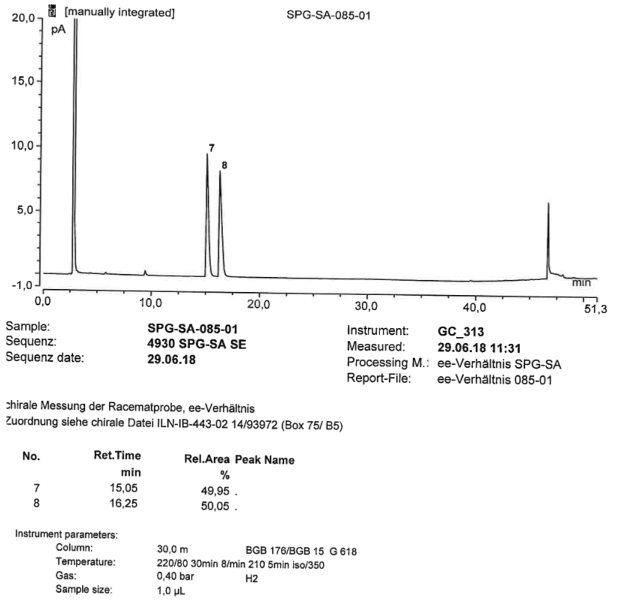

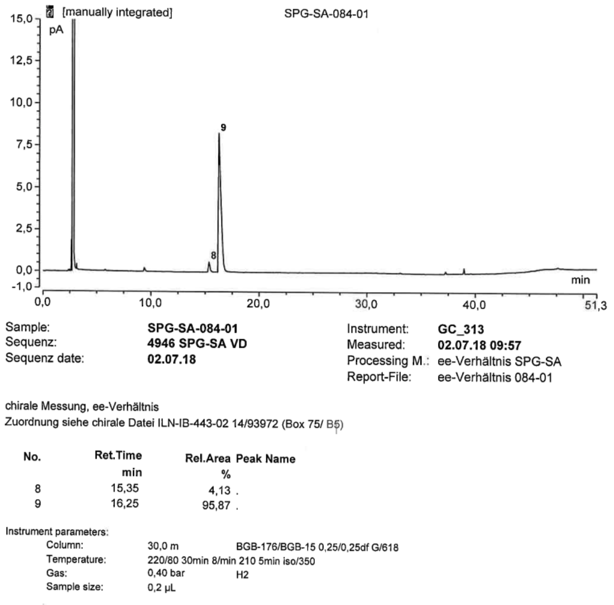

((2*S*,3*R*)-3-Ethyloxiran-2-yl)methanol (13b). A 250-mL Schlenk flask with a cooling jacket was

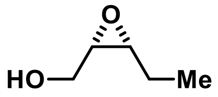

charged with powdered 4 Å molecular sieves (~1.5 g) and dichloromethane (60 mL). After the suspension had been cooled to −20 °C, titanium(IV) isopropoxide (1.1 mL, 3.7 mmol) and (+)-diisopropyl L-tartrate (0.95 mL, 4.5 mmol) were added sequentially and stirring was continued for 20 min at −20 °C before cumene hydroperoxide (80% technical grade, 6.5 mL, 35 mmol) was added. After stirring for further 30 min, a mixture of commercial (*Z*)-2-penten-1-ol (**12b**) (1.9 mL, 19 mmol) and powdered 4 Å molecular sieves (~0.5 g) in dichloromethane (40 mL) was added. The resulting mixture was stirred for 4.5 h at −20 °C. After the addition of a solution of citric acid monohydrate (775 mg) in diethyl ether/acetone (90 mL/10 mL), the mixture was warmed to room temperature and stirring was continued for 30 min before the orange suspension was filtered through a short pad of Celite®, which was rinsed with dichloromethane. The combined filtrates were concentrated under reduced pressure and the residue was purified by flash chromatography on silica gel (hexanes/EtOAc, 3:1) to give the title compound as a colorless liquid (1.40 g, 73% yield, 87% *ee*). $[\alpha]_D^{20} = -11.5$ (*c* 1.6, CH_2Cl_2). **^{1}H NMR (400 MHz, $CDCl_3$):** δ 3.86 (ddd, *J* = 11.8, 7.3, 4.2 Hz, 1H), 3.68 (ddd, *J* = 11.8, 6.9, 4.4 Hz, 1H), 3.17 (dt, *J* = 6.8, 4.2 Hz, 1H), 3.00 (ddd, *J* = 6.9, 6.0, 4.4 Hz, 1H), 1.70 – 1.48 (m, 3H), 1.05 (t, *J* = 7.5 Hz, 3H). **^{13}C{^{1}H} NMR (101 MHz, $CDCl_3$):** δ 60.9, 58.6, 57.2, 21.5, 10.8. The enantiomeric purity was determined by GC (see below). The analytical data are in agreement with those reported in the literature.[6]

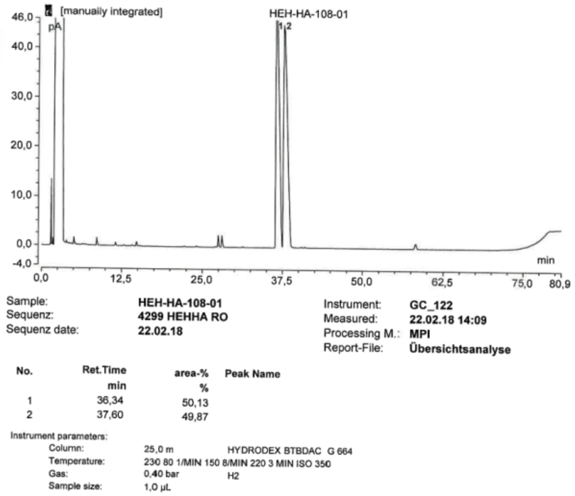

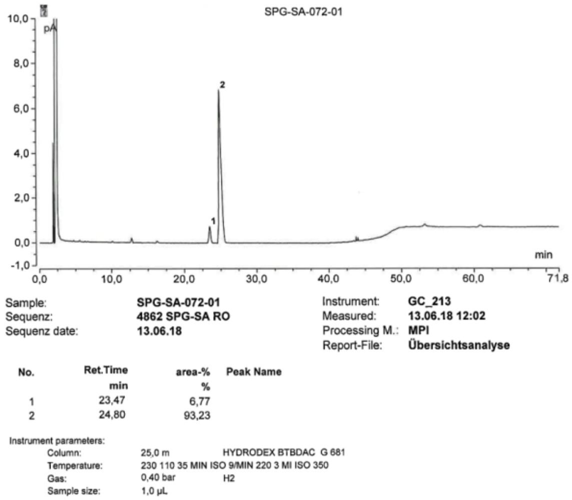

(2*R*,3*R*)-2-Ethynylbutane-1,3-diol (14a). In a 500-mL 2-necked flask, a solution of epoxy alcohol **13a** (3.98 g, 45.2 mmol) in THF (30 mL) was added to a suspension of lithium acetylide ethylene diamine complex (13.6 g, 133 mmol) in THF (120 mL) at 0°C. The mixture was gradually warmed to ambient temperature while stirring for 19 h. The reaction was carefully quenched at 0 °C by the addition of aqueous HCl (1 M, 20 mL) and the resulting mixture was neutralized with concentrated (37 w-%) aqueous HCl. Half-saturated NaCl solution (60 mL) and *tert*-butyl methyl ether (40 mL) were added and the layers were separated. The aqueous layer was extracted with *tert*-butyl methyl ether (1 × 50 mL), before it was further extracted overnight with *tert*-butyl methyl ether using a continuous liquid/liquid extraction apparatus. The combined organic layers were concentrated under reduced pressure and the residue was dissolved in dichloromethane (35 mL). Water (1.0 mL) and sodium periodate (1.69 g) were added and the mixture was vigorously stirred at room temperature for 1 h. For work-up, anhydrous sodium sulfate was added until the organic layer became clear, the solid materials were filtered off and the filtrate was evaporated under reduced pressure. Purification of the residue by flash chromatography (hexanes/EtOAc, 2:3) gave the title compound as a slightly yellowish oil, which crystallized upon standing at −20 °C to give oily needles (2.18 g, 42% yield, 89% *ee*). $[\alpha]_D^{20}$ **=** −8.2 (*c* 1.1, $CHCl_3$). **^{1}H NMR (400 MHz, $CDCl_3$):** δ 4.03 (qd, *J* = 6.3, 3.3 Hz, 1H), 3.90 – 3.78 (m, 2H), 2.63 (dddd, *J* = 6.1, 5.3, 3.3, 2.5 Hz, 1H), 2.35 (br s, 2H), 2.22 (d, *J* = 2.5 Hz, 3H). **^{13}C{^{1}H} NMR (101 MHz, $CDCl_3$):** δ 81.0, 73.3, 67.9, 63.9, 42.1, 21.3. **IR (film):** 3345, 3289, 2974, 2933, 2891, 1641, 1453, 1408, 1377, 1350, 1316, 1252, 1209, 1135, 1108, 1055, 1036, 986, 950, 904, 866, 809, 643, 554, 534, 490, 444 cm^{-1}. **HRMS-ESI *m/z*:** $[M+Na]^+$ calcd for $C_6H_{10}O_2Na$ 137.0573; found 137.0573. The enantiomeric purity was determined by GC (see below).

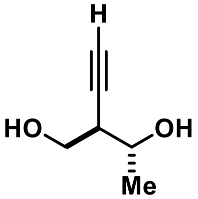

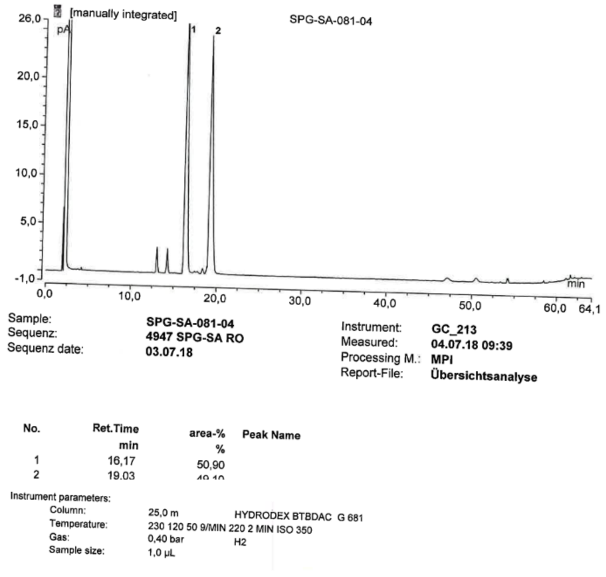

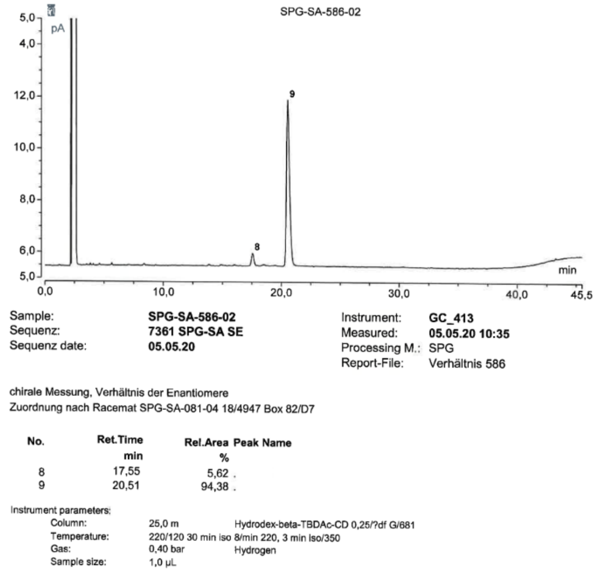

(2*R*,3*R*)-2-Ethynylpentane-1,3-diol (14b). In a 250-mL 2-necked flask, lithium acetylide ethylene diamine complex (8.60 g, 84.1 mmol) was added in one portion to a solution of epoxy alcohol **13b** (3.25 g, 31.8 mmol) in THF (100 mL) at 0 °C (ice bath). After stirring for 4.5 h, the reaction was carefully quenched at 0 °C by the addition of aqueous HCl (1 M, 10 mL) and the resulting mixture was neutralized by the addition of concentrated (37 w-%) aqueous HCl . Brine (40 mL) was added and the aqueous layer was extracted with *tert*-butyl methyl ether (3 × 50 mL), before it was further extracted overnight with *tert*-butyl methyl ether using a continuous liquid/liquid extraction apparatus. The combined organic layers were concentrated under reduced pressure and the residue dissolved in dichloromethane (50 mL). Water (2.5 mL) and sodium periodate (1.36 g) were added and the mixture was vigorously stirred for 1 h. Anhydrous sodium sulfate was then added until the organic layer became clear. The solids were filtered off and the filtrate was evaporated under reduced pressure. Purification of the residue by flash chromatography (hexanes/EtOAc, 1:1) gave the title compound as a slightly yellowish oil, which crystallized upon standing. Recrystallization from chloroform yielded the title compound as colorless needles in high enantiomeric purity (1.19 g, 29% yield, 97% *ee*). $[\alpha]_D^{20}$ = −1.1 (*c* 1.0, CH_2Cl_2). **^{1}H NMR (400 MHz, $CDCl_3$):** δ 3.91 – 3.81 (m, 2H), 3.71 (ddd, *J* = 8.1, 5.7, 2.9 Hz, 1H), 2.70 (ddd, *J* = 6.1, 5.3, 2.7 Hz, 1H), 2.20 (d, *J* = 2.5 Hz, 1H), 1.76-1.56 (m, 2H), 0.98 (t, *J* = 7.5 Hz, 3H). **^{13}C{^{1}H} NMR (101 MHz, $CDCl_3$):** δ 80.9, 73.27, 73.23, 64.2, 40.4, 28.7, 10.3. **IR (film):** 3355 (br), 3295, 2965, 2938, 2880, 1462, 1411, 1340, 1243, 1124, 1046, 958, 803, 642 cm^{-1}. **HRMS-ESI *m/z*:** $[M+Na]^+$ calcd for $C_7H_{12}O_2Na$ 151.0729; found 151.0730. The enantiomeric purity was determined by GC (see below). The spectroscopic data are in agreement with those reported in the literature.[6,7]

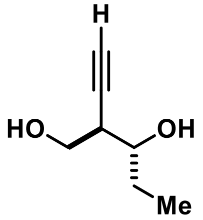

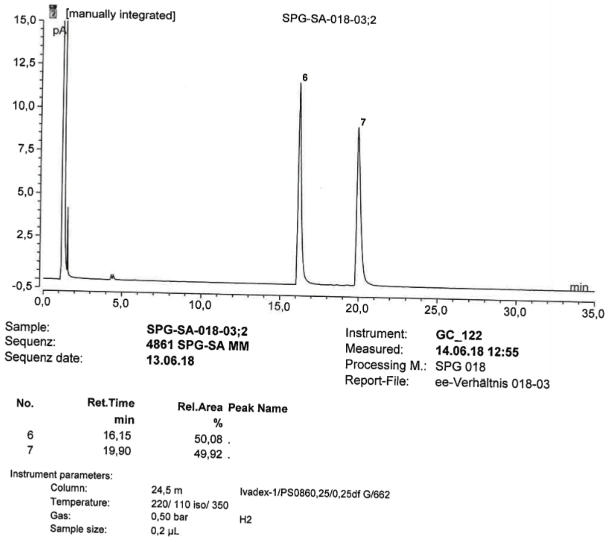

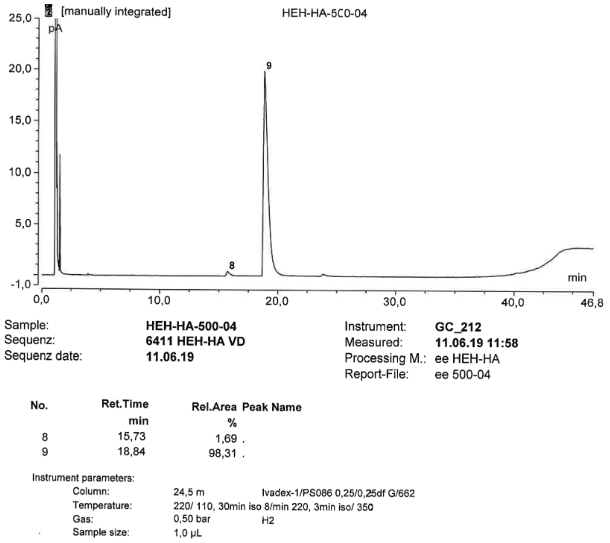

(2*R*,3*R*)-3-(((*tert*-Butyldiphenylsilyl)oxy)methyl)pent-4-yn-2-ol (S1). In a 100-mL two-necked

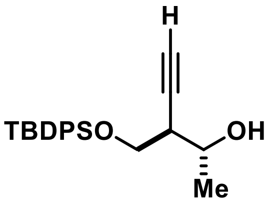

flask, *tert*-butyldiphenylsilyl chloride (2.1 mL, 8.1 mmol) was added a solution of diol **14a** (746 mg, 6.54 mmol) and imidazole (561 mg, 8.24 mmol) in dichloromethane (40 mL) at 0 °C. The ice bath was removed and the mixture stirred for 1 h at room temperature. The mixture was washed with saturated aqueous sodium bicarbonate solution (20 mL) and the aqueous phase was extracted with dichloromethane (3 × 15 mL). The combined organic layers were dried over anhydrous sodium sulfate, the drying agent was filtered off and the solvent was removed under reduced pressure. Purification of the residue by flash chromatography (hexanes/EtOAc, 10:1) led to the title compound as a colorless syrup (1.92 g, 83% yield). $[\alpha]_D^{20}$ **=** +12.1 (*c* 1.3, $CHCl_3$). **^{1}H NMR (400 MHz, $CDCl_3$):** δ 7.74 – 7.64 (m, 4H), 7.48 – 7.35 (m, 6H), 4.14 (quintd, *J* = 6.4, 3.0 Hz, 1H), 3.94 – 3.80 (m, 2H), 2.62 (ddt, *J* = 7.5, 4.9, 2.5 Hz, 1H), 2.39 (d, *J* = 6.6 Hz, 1H), 2.14 (d, *J* = 2.5 Hz, 1H), 1.32 (d, *J* = 6.3 Hz, 3H), 1.07 (s, 9H). **^{13}C{^{1}H} NMR (101 MHz, $CDCl_3$):** δ 135.8, 135.7, 134.9, 133.2, 133.0, 130.0, 129.8, 127.95, 127.92, 127.86, 81.2, 72.7, 66.9, 64.7, 42.0, 27.0, 21.4, 19.4. **IR (film):** 3468, 3305, 3071, 3050, 2960, 2931, 2887, 2858, 1590, 1472, 1428, 1391, 1362, 1260, 1189, 1111, 998, 938, 823, 740, 702, 641, 613, 543, 505 cm^{-1}. **HRMS-ESI *m/z*:** $[M+Na]^+$ calcd for $C_{22}H_{28}O_2SiNa$ 375.1751; found 375.1752.

(2*R*,3*R*)-3-(((*tert*-Butyldiphenylsilyl)oxy)methyl)-2-(triethylsilyloxy)-pent-4-yne (S2). In a 100-mL 2-necked flask, triethylsilyl trifluoromethanesulfonate (2.7 mL, 12 mmol) was added to a solution of alcohol **S1** (3.27 g, 9.28 mmol) and 2,6-lutidine (1.7 mL, 15 mmol) in dichloromethane (60 mL) at 0 °C. The mixture was gradually warmed while stirring for 15 h. The reaction was quenched with saturated aqueous sodium bicarbonate solution (30 mL) and diluted with dichloromethane (10 mL). The aqueous phase was extracted with dichloromethane (5 × 15 mL), the combined organic layers were washed with brine (1 × 20 mL) and dried over anhydrous sodium sulfate. The drying agent was filtered off and the filtrate was concentrated under reduced pressure. Flash chromatography (hexanes/EtOAc, 100:1) furnished the title compound as a colorless oil (4.16 g, 96% yield). $[\alpha]_D^{20}$ = +15.7 (*c* 1.3, $CHCl_3$). **^{1}H NMR (400 MHz, $CDCl_3$):** δ 7.71 – 7.65 (m, 4H), 7.45 – 7.34 (m, 6H), 4.15 (qd, *J* = 6.3, 3.8 Hz, 1H), 3.89 – 3.74 (m, 2H), 2.55 (dddd, *J* = 7.0, 6.2, 3.8, 2.5 Hz, 1H), 2.05 (d, *J* = 2.5 Hz, 1H), 1.25 (d, *J* = 6.3 Hz, 3H), 1.06 (s, 9H), 0.94 (t, *J* = 7.9 Hz, 9H), 0.67 – 0.50 (m, 6H). **^{13}C{^{1}H} NMR (101 MHz, $CDCl_3$):** δ 135.8, 133.8, 129.8, 127.8, 83.1, 71.2, 66.7, 63.4, 43.3, 27.0, 21.8, 19.4, 7.05, 5.20. **IR (film):** 3312, 3072, 3051, 2955, 2933, 2910, 2876, 2859, 1590, 1462, 1428, 1414, 1377, 1362, 1239, 1188, 1148, 1110, 1077, 1006, 980, 939, 892, 823, 784, 738, 637, 613, 505 cm^{-1}. **HRMS-ESI *m/z*:** [M+Na]$^+$ calcd for $C_{28}H_{42}O_2Si_2Na$ 489.2616; found 489.2617.

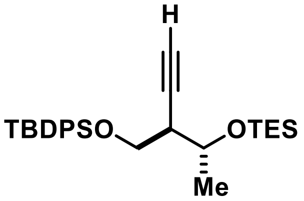

(3*R*,4*R*)-4-(((*tert*-Butyldiphenylsilyl)oxy)methyl)-3-(triethylsilyloxy)-hex-5-yne (S3). In a 100-mL round-bottomed flask, *tert*-butyldiphenylsilyl chloride (2.7 mL, 10 mmol) was added to a solution of diol **14b** (1.19 g, 9.30 mmol) and imidazole (760 mg, 11.2 mmol) in dichloromethane (60 mL) at 0 °C. The ice bath was removed and the mixture stirred for 4 h at room temperature. Filtration of the white suspension through a short plug of silica, which was rinsed with dichloromethane, and concentration of the combined filtrates under reduced pressure led to a colorless oil (3.78 g).

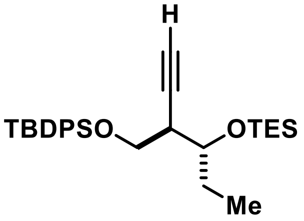

In a 100-mL 2-necked flask, triethylsilyl trifluoromethanesulfonate (3.2 mL, 14 mmol) was added to a solution of this crude material and 2,6-lutidine (2.2 mL, 19 mmol) in dichloromethane (60 mL) at 0 °C. The mixture was allowed to gradually warm to ambient temperature over the course of 14 h. The reaction was quenched with saturated aqueous sodium bicarbonate solution (20 mL) and the mixture was diluted with dichloromethane (15 mL). The aqueous layer was extracted with dichloromethane (3 × 15 mL) and the combined organic layers were dried over anhydrous sodium sulfate. The drying agent was removed by filtration and the filtrate was concentrated under reduced pressure. Flash chromatography (hexanes/EtOAc, 100:1) furnished the title compound as a colorless oil (4.08 g, 91% yield). $[\alpha]_D^{20}$ = +15.8 (*c* 1.3, $CHCl_3$).

^{1}H NMR (400 MHz, CDCl$_3$): δ 7.71 – 7.65 (m, 4H), 7.46 – 7.33 (m, 6H), 3.89 – 3.81 (m, 2H), 3.76 (dd, *J* = 9.8, 6.4 Hz, 1H), 2.70 – 2.63 (m, 1H), 2.02 (d, *J* = 2.5 Hz, 1H), 1.80 – 1.67 (m, 1H), 1.58 – 1.46 (m, 1H), 1.06 (s, 9H), 0.93 (t, *J* = 7.9 Hz, 9H), 0.86 (t, *J* = 7.8 Hz, 3H), 0.65 – 0.54 (m, 6H). **^{13}C{^{1}H} NMR (101 MHz, CDCl$_3$):** δ 135.80, 135.77, 135.26, 133.78, 133.73, 129.79, 129.76, 127.78, 127.76, 127.5, 82.9, 72.2, 71.1, 63.5, 40.6, 28.2, 27.0, 19.4, 10.5, 7.10, 5.33. **IR (film):** 3311, 3072, 2958, 2934, 2877, 1462, 1428, 1380, 1239, 1112, 1010, 824, 739, 702, 637, 613, 506 cm^{-1}. **HRMS-ESI *m/z*:** [M+Na]$^+$ calcd for $C_{29}H_{44}O_2Si_2Na$ 503.2772; found 503.2771.

(2*R*,3*R*,*E*)-3-(((*tert*-Butyldiphenylsilyl)oxy)methyl)-5-iodo-2-(triethylsilyloxy)-pent-4-ene

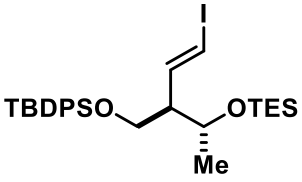

(15a). In a 500-mL two-necked flask, a solution of diisobutylaluminum hydride in hexanes (1.0 M, 24 mL, 24 mmol) was added over 10 min to a solution of zirconocene dichloride (6.96 g, 24.7 mmol) in THF (50 mL) at 0 °C. After stirring for 30 min at this temperature, a solution of alkyne **S2** (9.59 g, 20.5 mmol) in THF (50 mL) was added over 15 min, the cooling bath was removed, and stirring was continued for 1 h. During this time, the originally greyish suspension turned into a very dark green homogeneous solution. 2,6-Lutidine (3.1 mL, 27 mmol) was added and the resulting mixture was cooled to −78 °C. A solution of iodine (6.28 g, 24.7 mmol) in THF (40 mL) was added over 20 min and stirring was continued for 1 h at this temperature. The reaction was quenched with a mixture of saturated aqueous solutions of sodium bicarbonate and sodium thiosulfate (1:1, 100 mL), and the mixture was diluted with *tert*-butyl methyl ether (100 mL) and allowed to reach room temperature. After separation of the two layers in a separatory funnel, both were filtered separately through a pad of Celite®, which was rinsed with *tert*-butyl methyl ether (3 × 20 mL). The aqueous filtrate was extracted with *tert*-butyl methyl ether (3 × 30 mL) and the combined organic layers were dried over anhydrous sodium sulfate. The drying agent was filtered off and the solvent was removed under reduced pressure. Purification of the residue by flash chromatography (hexanes/EtOAc, 100:1 → 15:1) led to the title compound as a slightly amber oil (7.97 g, 65% yield). $[\alpha]_D^{20}$ = +23.6 (*c* 1.2, CHCl$_3$). **^{1}H NMR (400 MHz, CDCl$_3$):** δ 7.66 – 7.61 (m, 4H), 7.46 – 7.34 (m, 6H), 6.49 (dd, *J* = 14.5, 9.6 Hz, 1H), 5.99 (dd, *J* = 14.5, 0.6 Hz, 1H), 4.04 (qd, *J* = 6.37, 3.7 Hz, 1H), 3.73 (dd, *J* = 10.1, 6.3 Hz, 1H), 3.56 (dd, *J* = 10.1, 6.5 Hz, 1H), 2.15 (ddt, *J* = 9.9, 6.4, 3.2 Hz, 1H), 1.08 (d, *J* = 6.3 Hz, 3H), 1.05 (s, 9H), 0.91 (t, *J* = 7.9 Hz, 9H), 0.62 – 0.47 (m, 6H). **^{13}C{^{1}H} NMR (101 MHz, CDCl$_3$):** δ 144.7, 135.8, 135.7, 133.80, 133.76, 129.81, 129.77, 127.82, 127.80, 127.7, 77.3, 66.9, 64.0, 56.8, 27.0, 22.3, 19.4, 7.08, 5.18. **IR (film):** 3071, 3050, 2956, 2931, 2910, 2875, 2858, 1603, 1590, 1471, 1462, 1428, 1376, 1361, 1259, 1241, 1178, 1146, 1111, 1092, 1008, 953, 824, 801, 738, 701, 614, 504, 489 cm^{-1}. **HRMS-ESI *m/z*:** [M+Na]$^+$ calcd for $C_{28}H_{43}IO_2Si_2Na$ 617.1739; found 617.1744.

(3*R*,4*R*,*E*)-4-(((*tert*-Butyldiphenylsilyl)oxy)methyl)-6-iodo-3-(triethylsilyloxy)-hex-5-ene

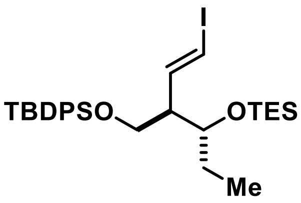

(15b). In a 100-mL two-necked flask, a solution of diisobutylaluminum hydride in toluene (25 w-%, 7.0 mL, 9.8 mmol) was added to a solution of zirconocene dichloride (2.91 g, 10.3 mmol) in THF (20 mL) at 0 °C. After stirring for 30 min, a solution of alkyne **S3** (4.00 g, 8.32 mmol) in THF (10 mL) was added over 5 min. The cooling bath was removed and stirring continued for 1 h, during which time the originally greyish suspension turned into a very dark green homogeneous solution. 2,6-Lutidine (1.3 mL, 11 mmol) was introduced and the mixture cooled to −78 °C. A solution of iodine (2.74 g, 10.8 mmol) in THF (15 mL) was added over 15 min and stirring was continued for 1 h at −78 °C. The reaction was quenched by addition of a mixture of saturated aqueous solutions of sodium bicarbonate and sodium thiosulfate (1:1, 30 mL), the mixture was diluted with *tert*-butyl methyl ether (20 mL) and warmed to room temperature. The mixture was filtered through a short pad of Celite®, which was rinsed with *tert*-butyl methyl ether (5 × 10 mL). The layers were separated and the aqueous phase was extracted with *tert*-butyl methyl ether (4 × 20 mL). The combined organic layers were dried over anhydrous sodium sulfate, the drying agent was filtered off, and the solvent was removed under reduced pressure. Purification by flash chromatography (hexanes/EtOAc, 100:1) led to the title compound as a slightly amber oil (3.76 g, 74% yield). $[\alpha]_D^{20}$ = +19.3 (*c* 1.2, $CHCl_3$). **^{1}H NMR (400 MHz, $CDCl_3$):** δ 7.68 – 7.61 (m, 4H), 7.46 – 7.34 (m, 6H), 6.46 (dd, *J* = 14.5, 9.6 Hz, 1H), 5.94 (dd, *J* = 14.5, 0.6 Hz, 1H), 3.79 – 3.68 (m, 2H), 3.54 (dd, *J* = 10.0, 6.9 Hz, 1H), 2.28 (ddt, *J* = 9.8, 6.8, 3.0 Hz, 1H), 1.47 – 1.38 (m, 2H), 1.05 (s, 9H), 0.90 (t, *J* = 7.9 Hz, 9H), 0.77 (t, *J* = 7.5 Hz, 3H), 0.61 – 0.48 (m, 6H). **^{13}C{^{1}H} NMR (101 MHz, $CDCl_3$):** δ 144.3, 135.8, 135.7, 133.9, 133.8, 129.83, 129.76, 127.81, 127.79, 77.3, 72.4, 64.2, 53.3, 28.4, 27.0, 19.3, 9.95, 7.09, 5.30. **IR (film):** 3071, 3052, 2956, 2933, 2875, 1601, 1488, 1462, 1428, 1390, 1241, 1159, 1111, 1001, 970, 844, 824, 804, 767, 740, 616, 505, 488 cm^{-1}. **HRMS-ESI *m/z*:** $[M+Na]^+$ calcd for $C_{29}H_{45}IO_2Si_2Na$ 631.1895; found 631.1893.

(3*R*,4*R*,*E*)-4-(((*tert*-Butyldiphenylsilyl)oxy)methyl)-3-(triethylsilyloxy)-oct-5-en-7-yne (16). In

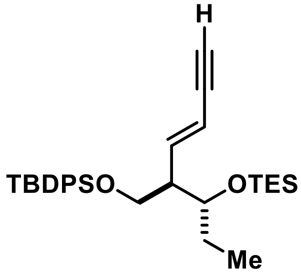

a 100-mL 2-necked flask, bis(triphenylphosphine)palladium(II) dichloride (107 mg, 0.152 mmol) was added to a solution of **15b** (3.68 g, 6.05 mmol) in triethylamine (30 mL) at room temperature . The yellow mixture was stirred for 45 min before the flask was immersed in an ice bath and trimethylsilylacetylene (1.2 mL, 8.5 mmol) and copper(I) iodide (290 mg, 1.52 mmol) were added. The originally yellow solution immediately turned into a brown suspension. After stirring for 20 min, the ice bath was removed and stirring continued for 18 h at room temperature. The mixture was diluted with *tert*-butyl methyl ether (35 mL) and filtered through filter paper, before it was washed with saturated aqueous ammonium chloride solution (2 × 25 mL). The combined aqueous phases were extracted with *tert*-butyl methyl ether (4 × 15 mL) and the organic layers

were dried over anhydrous sodium sulfate. The drying agent was filtered off and the solvent was removed under reduced pressure. Purification of the residue by flash chromatography (hexanes/EtOAc, 100:1) led to a yellowish oil (3.51 g).

Anhydrous potassium carbonate (1.68 g, 12.2 mmol) was added in one portion to a solution of this material in THF/methanol (1:1, 50 mL) at room temperature. Stirring was continued for 2.5 h, before the mixture was concentrated under reduced pressure. The residue was dissolved in *tert*-butyl methyl ether (50 mL), the organic phase was washed with aqueous HCl (1 M, 30 mL) and the aqueous layer was extracted with *tert*-butyl methyl ether (3 × 15 mL). The combined organic layers were dried over anhydrous sodium sulfate, the drying agent was filtered off, and the solvent was removed under reduced pressure. Purification of the crude material by flash chromatography (hexanes/EtOAc, 100:1) led to the title compound as a yellowish oil (2.93 g, 96% yield). $[\alpha]_D^{20}$ **=** +19.2 (*c* 1.2, $CHCl_3$). **^{1}H NMR (400 MHz, $CDCl_3$):** δ 7.67 – 7.62 (m, 4H), 7.45 – 7.34 (m, 6H), 6.18 (ddd, *J* = 16.1, 9.4, 0.6 Hz, 1H), 5.39 (ddd, *J* = 16.1, 2.2, 0.8 Hz, 1H), 3.82 (td, *J* = 6.8, 2.7 Hz, 1H), 3.75 (dd, *J* = 10.0, 7.1 Hz, 1H), 3.53 (dd, *J* = 10.0, 6.7 Hz, 1H), 2.80 (dd, *J* = 2.3, 0.62 Hz, 1H), 2.33 – 2.24 (m, 1H), 1.46 – 1.37 (m, 2H), 1.06 (s, 9H), 0.91 (t, *J* = 7.9 Hz, 9H), 0.77 (t, *J* = 7.5 Hz, 3H), 0.62 – 0.49 (m, 6H). **^{13}C{^{1}H} NMR (101 MHz, $CDCl_3$):** δ 144.2, 135.8, 135.7, 133.9, 133.8, 129.8, 129.7, 127.77, 127.76, 111.4, 82.7, 76.1, 72.7, 64.5, 50.4, 28.5, 27.0, 19.4, 10.1, 7.10, 5.30. **IR (film):** 3313, 3071, 2957, 2933, 2876, 2588, 1462, 1428, 1379, 1240, 1111, 1081, 1008, 963, 823, 738, 702, 612, 504 cm^{-1}. **HRMS-ESI *m/z*:** $[M+H]^+$ calcd for $C_{31}H_{47}O_2Si_2$ 507.3109; found 507.3114.

(2*R*,3*R*,*E*)-3-(((*tert*-Butyldiphenylsilyl)oxy)methyl)-2-(triethylsilyloxy)-oct-4-en-7-yne (17). In a 250-mL two-necked flask, *n*-BuLi (1.6 M in hexanes, 25 mL, 40 mmol) was added over 10 min to a solution of 1-(trimethylsilyl)propyne (7.8 mL, 53 mmol) in THF (100 mL) at –78 °C. The resulting pale yellowish solution was warmed to 0 °C and stirred for 1 h at that temperature. This solution was then transferred via cannula into a suspension of copper(I) iodide (7.67 g, 40.3 mmol) in THF (50 mL) at –78 °C in a 500-mL two-necked flask. After the addition, a greenish/sand-colored suspension had formed, which was stirred for additional 30 min. 4-(Dimethylamino)pyridine (4.91 g, 40.2 mmol) in THF (60 mL) was then introduced and the mixture was gradually warmed to –20 °C over 30 min, eventually leading to a clear, dark brown solution after stirring for further 20 min. At this point, a solution of alkenyl iodide **15a** (7.97 g, 13.4 mmol) in THF (40 mL) was added over 5 min. The mixture was stirred for 17 h, while being gradually warmed to room temperature. Saturated aqueous ammonium chloride solution (10 mL) was introduced, leading to a thick brown precipitation, which was filtered off through a glass frit (pore size 4) and the remaining solids were carefully rinsed with *tert*-butyl methyl ether (5 × 15 mL). The combined filtrates were washed with saturated aqueous ammonium chloride

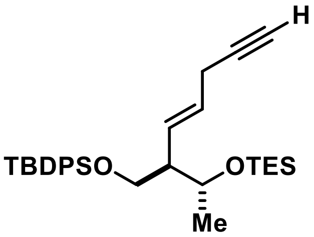

solution (3 × 75 mL) and the combined aqueous phases were extracted with *tert*-butyl methyl ether (3 × 30 mL). The organic layers were dried over anhydrous sodium sulfate, the drying agent was filtered off, and the solvent was removed under reduced pressure. Purification of the residue by flash chromatography (hexanes/EtOAc, 100:1) led to an amber oil (7.73 g).

Anhydrous potassium carbonate (3.70 g, 26.8 mmol) was added to a solution of this material in THF/methanol (1:1, 120 mL), and the resulting suspension was vigorously stirred for 4 h at room temperature (reaction monitoring by ^{1}H NMR). Volatile materials were evaporated and the residue was dissolved in *tert*-butyl methyl ether (90 mL). Aqueous HCl (1 M, 80 mL) was carefully added at 0 °C, the aqueous layer was extracted with *tert*-butyl methyl ether (3 × 25 mL) and the combined organic layers were dried over anhydrous sodium sulfate. The drying agent was filtered off and the solvent was removed under reduced pressure. Purification of the residue by flash chromatography (hexanes/EtOAc, 100:1) led to the title compound as a light amber oil (5.72 g, 84% yield). $[\alpha]_D^{20}$ **=** +11.5 (*c* 1.0, $CHCl_3$). **^{1}H NMR (400 MHz, $CDCl_3$):** δ 7.70 – 7.60 (m, 4H), 7.45 – 7.33 (m, 6H), 5.68 (ddt, *J* = 15.4, 9.1, 1.7 Hz, 1H), 5.39 (dtd, *J* = 15.4, 5.6, 0.7 Hz, 1H), 4.10 (qd, *J* = 6.2, 3.7 Hz, 1H), 3.75 (dd, *J* = 10.0, 6.6 Hz, 1H), 3.58 (dd, *J* = 10.0, 6.2 Hz, 1H), 2.91 (ddd, *J* = 5.6, 2.7, 1.7 Hz, 2H), 2.14 (dtd, *J* = 9.8, 6.3, 3.6 Hz, 1H), 2.06 (t, *J* = 2.7 Hz, 1H), 1.09 (t, *J* = 6.3 Hz, 3H), 1.05 (s, 9H), 0.92 (t, *J* = 7.9 Hz, 9H), 0.63 – 0.48 (m, 6H). **^{13}C{^{1}H} NMR (101 MHz, $CDCl_3$):** δ 135.80, 135.77, 134.1, 130.3, 129.66, 129.63, 127.7, 126.5, 82.2, 69.9, 67.6, 64.8, 53.0, 27.1, 22.2, 22.1, 19.4, 7.08, 5.24. **IR (film):** 3312, 3071, 3049, 2956, 2932, 2911, 2876, 2858, 1590, 1472, 1462, 1427, 1375, 1240, 1188, 1150, 1111, 1007, 972, 824, 807, 739, 702, 624, 614, 505, 489 cm^{-1}. **HRMS-ESI *m/z*:** $[M+Na]^+$ calcd for $C_{31}H_{46}O_2Si_2Na$ 529.2929; found 529.2927.

Preparation of the Common Eastern Fragment

(*S*)-2-Methylpent-4-en-1-yl acetate (4). A 1-L three-necked flask with cooling jacket equipped with

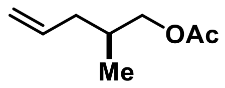

an argon bubbler was charged with Amano Lipase (*pseudomonas fluorescens*) (2.15 g), tetrahydrofuran (430 mL) and 2-methyl-4-penten-1-ol (21.6 g, 215 mmol). The mixture was cooled to −20 °C using a cryostat while the mixture was gently stirred (~250 rpm). Vinyl acetate (29.9 mL, 322 mmol) was added in one portion and stirring continued for 11.5 h at −20 °C. Once the reaction progress had been determined to have reached 45% (GC-FID), the enzyme was filtered off from the cold mixture using a fritted funnel. The filtrate was carefully concentrated to remove most of the solvent. Purification of this concentrated solution by flash chromatography (pentane/diethylether, 9:1) afforded the title compound as a colorless liquid (15.1 g, 40% yield, 94% *ee*). $[\alpha]_D^{20}$ **=** −2.3 (*c* 1.0, $CHCl_3$). **^{1}H NMR (400 MHz, $CDCl_3$):** δ 5.83 – 5.70 (m, 1H), 5.07 – 5.00 (m, 2H), 3.96 (dd, *J* = 10.8, 6.1 Hz, 1H), 3.88 (dd,

J = 10.8, 6.4 Hz, 1H) 2.20 – 2.11 (m, 1H), 2.06 (s, 3H), 1.99 – 1.81 (m, 2H), 1.36 – 1.18 (m, 1H), 0.93 (d, *J* = 6.6 Hz, 3H). **^{13}C{^{1}H} NMR (101 MHz, CDCl$_3$):** δ 171.4, 136.3, 116.7, 68.9, 37.9, 32.5, 21.1, 16.7. **IR (film):** 3078, 2964, 2934, 2914, 1738, 1642, 1461, 1441, 1390, 1366, 1232, 1035, 933, 912 cm^{-1}. **HRMS (GC-CI) *m/z*:** [M]$^+$ calcd for $C_8H_{15}O_2$ 143.1067; found 143.1066. The enantiomeric purity was determined by GC (see below).

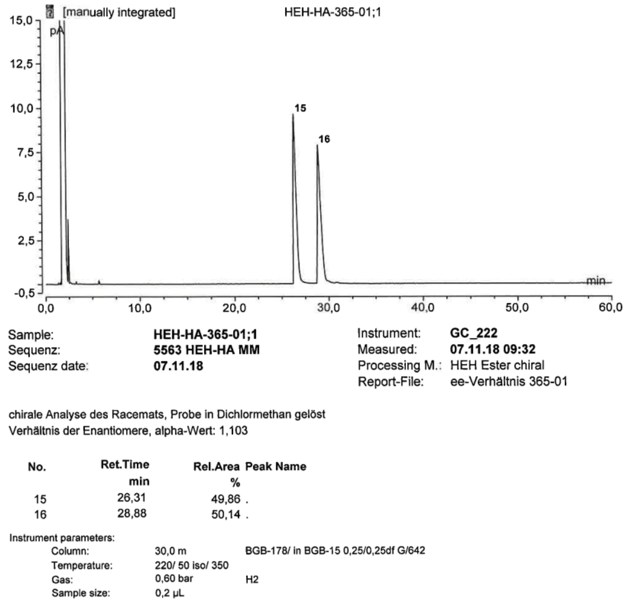

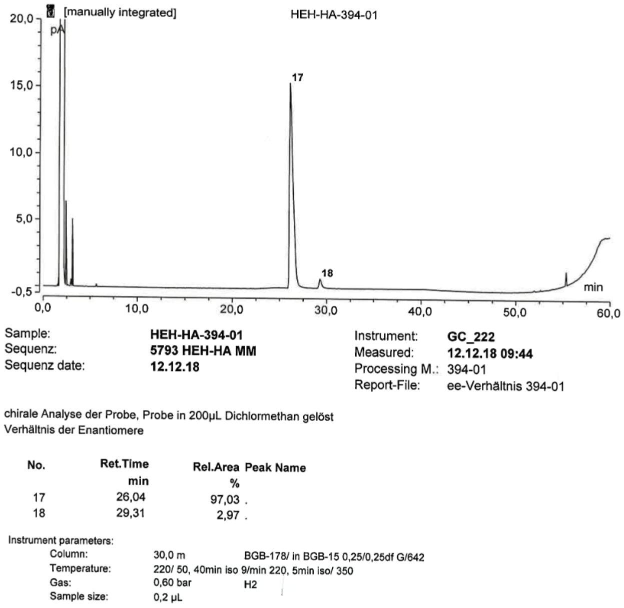

(*S*)-2-Methylpent-4-en-1-ol (5). Methyl lithium in diethyl ether (1.6 M, 64 mL, 102 mmol) was slowly

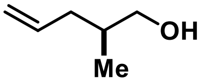

added to a solution of acetate **4** (15.1 g, 85.0 mmol) in diethylether (200 mL) at −30 °C. Once the addition was complete, the mixture was warmed to room temperature and stirring was continued for another 3 h. The reaction was quenched by the addition of water and the mixture was extracted twice with diethylether. The combined organic phases were washed with brine and evaporated, and the residue was purified by flash chromatography (pentane/diethyl ether, 5:1) to afford the title compound as a colorless liquid (6.40 g, 75% yield). $[\alpha]_D^{20}$ = −3.4 (*c* 1.0, CHCl$_3$). **^{1}H NMR (400 MHz, CDCl$_3$):** δ 5.81 (ddt, *J* = 17.2, 10.1, 7.2 Hz, 1H), 5.08 – 4.99 (m, 2H), 3.55 – 3.43 (m, 2H), 2.18 (dddt, *J* = 14.3, 7.2, 6.0, 1.3 Hz, 1H), 2.00 – 1.90 (m, 1H), 1.81 – 1.68 (m, 1H), 0.93 (d, *J* = 6.8 Hz, 3H). **^{13}C{^{1}H} NMR (101 MHz, CDCl$_3$):** δ 137.1, 116.2, 68.1, 38.00, 35.8, 16.5. **IR (film):** 3321, 3077, 2957, 2913, 2875, 1640, 1456, 1439, 1378, 1040, 1028, 992, 909, 628 cm^{-1}. **HRMS-ESI *m/z*:** [M+H]$^+$ calcd for $C_6H_{13}O$ 101.0961, found 101.0961.

(*S*)-2-Methylpent-4-enal (6). In a 250-mL three-necked flask fitted with a dropping funnel, a

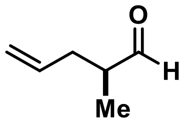

thermometer and an argon inlet, dimethyl sulfoxide (14 mL, 200 mmol) was added over 5 min to a solution of oxalyl chloride (9.0 mL, 104 mmol) in dichloromethane (90 mL) at −78 °C, making sure that the internal temperature stayed below −50 °C at all time. After an additional 5 min, alcohol **5** (9.50 g, 94.8 mmol) was added as a solution in dichloromethane (30 mL) over the course of 30 min. After stirring for additional 30 min, triethylamine (66 mL, 101 mmol) was introduced over 5 min, leading to the formation of a thick white suspension, which was vigorously stirred. After 5 min, the mixture was warmed to room temperature and partitioned between water and dichloromethane. The aqueous phase was extracted with dichloromethane, and the combined organic layers were washed with an aqueous solution of HCl (0.5 M) and brine. They were dried over anhydrous magnesium sulfate, the drying agent was filtered off and the solution was concentrated under reduced pressure (800 mbar, 30 °C). Pentane was added to azeotropically remove most of the dimethylsulfide. Because of the volatility of the title compound, the resulting solution was used without further purification in the next step (29.0 g, ~29 w-% by ^{1}H NMR). **^{1}H NMR (400 MHz, $CDCl_3$)**: δ 9.66 (d, *J* = 1.4 Hz, 1H), 5.83 – 5.70 (m, 1H), 5.14 – 5.04 (m, 2H), 2.52 – 2.40 (m, 2H), 2.21 – 2.11 (m, 1H), 1.11 (d, *J* = 7.0 Hz, 3H).

Methyl (*4S,5S,6S,E*)-5-hydroxy-4,6-dimethylnona-2,8-dienoate (7). ***Catalyst preparation***: A

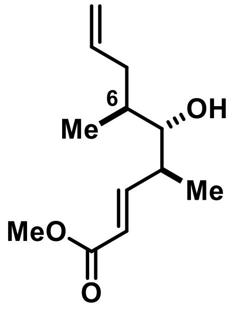

100-mL two-necked flask equipped with a Dean-Stark trap topped by a reflux condenser and argon bubbler was charged with (*R*)-diphenylprolinol (10.0 g, 38.8 mmol) and phenylboronic acid (4.98 g, 38.8 mmol). Toluene (200 mL) was added and the mixture was stirred at reflux temperature for 14 h. After cooling the mixture to 50 °C, the Dean-Stark condenser was replaced by a connection to a vacuum manifold to remove the toluene in vacuum at 50 °C. The residue was dried in high vacuum for 2 h before the semisolid was dissolved in dichloromethane (530 mL).

A three-necked flask with cooling jacked equipped with a 100-mL dropping funnel and an argon bubbler was charged with this catalyst solution. The solution was cooled to −78 °C before freshly distilled triflic acid (2.8 mL, 32 mmol) was added in one portion, causing the immediate formation of a red/orange precipitate. The mixture was stirred at −78 °C until all the solid material had disappeared (20 to 60 min). The dropping funnel was then charged with a solution of aldehyde **6** (28.7 g, 29 w-% in CH_2Cl_2, 86 mmol), dichloromethane (225 mL), ketene acetal **9** (16.0 g, 69.9 mmol) and isopropanol (5.4 mL, 70 mmol) in that order. The mixture in the dropping funnel was added over the course of 2 h to the catalyst solution at −78 °C. After complete addition, the mixture was stirred for an additional hour at this temperature before the reaction was quenched with saturated aqueous sodium bicarbonate solution (50 mL). The resulting mixture was warmed to room temperature, the aqueous phase was extracted thrice with dichloromethane, and the

combined organic layers were stirred vigorously with aqueous HCl (2 M, 400 mL) for 1 h. The resulting aqueous phase was extracted thrice with dichloromethane and the combined organic layers were washed with brine and dried over anhydrous magnesium sulfate. The drying agent was filtered off and the solution was concentrated under reduced pressure. The residue was purified by flash chromatography (hexanes/EtOAc, 9:1) to afford the title compound admixed with the C-6 epimer as a colorless oil (10.2 g, 69% yield, 89:11 d.r.). $[\alpha]_D^{20}$ = −15.7 (*c* 1.0, $CHCl_3$). **^{1}H NMR (400 MHz, $CDCl_3$):** δ 6.97 (dd, *J* = 15.8, 7.6 Hz, 1H), 5.87 (dd, *J* = 15.8, 1.3 Hz, 1H), 5.84 – 5.73 (m, 1H), 5.08 – 4.99 (m, 2H), 3.73 (s, 3H), 3.36 (dt, *J* = 6.9, 5.0 Hz, 1H), 2.62 – 2.52 (m, 1H), 2.40 – 2.31 (m, 1H), 1.95 (dtt, *J* = 13.9, 8.4, 1.1 Hz, 1H), 1.74 – 1.62 (m, 1H), 1.58 (d, *J* = 5.2 Hz, 1H), 1.08 (d, *J* = 6.8 Hz, 3H), 0.90 (d, *J* = 6.9 Hz, 3H). **^{13}C{^{1}H} NMR (101 MHz, $CDCl_3$):** δ 167.2, 152.4, 137.2, 121.0, 116.6, 78.1, 51.7, 39.5, 36.5, 36.1, 16.3, 12.9. **IR (film):** 3470, 2969, 2932, 2910, 2879, 1704, 1653, 1642, 1456, 1436, 1276, 1195, 1178, 986, 911, 864 754 cm^{-1}. **HRMS-ESI *m/z*:** $[M+Na]^+$ calcd for $C_{12}H_{20}O_3Na$ 235.1305, found 235.1306.

Stereochemical Analysis of Product **7** *formed by the Vinylogous Mukaiyama Aldol Reaction*

(1) The identity of the minor diastereoisomer (C-6 epimer) was established by comparison of the crude mixture obtained under the reaction conditions described above to the reaction outcome when racemic aldehyde **6** was used under otherwise identical conditions. Since no other diastereomers were observed by NMR analysis of the crude product mixture, the aldol reaction itself seems to proceed with a *dr* > 98:2.

(2) The relative configuration at C-5 was determined by Mosher ester analysis of alcohol **7**.

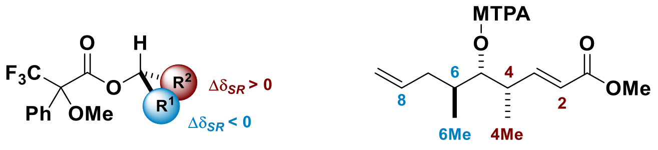

	δ_S (44a)	δ_R (44b)	$\Delta\delta_{SR}$
2	5.83	5.81	+0.02
3	6.85	6.82	+0.03
4	2.75	2.73	+0.02
4Me	1.02	0.99	+0.03
5	5.06	5.06	0.00
6	1.86	1.88	-0.02
6Me	0.86	0.89	-0.03
8	5.66	5.67	-0.01

Table S1. Comparison of chemical shifts of ^{1}H NMR signals for (*S*)-MTPA (δ_S) and (*R*)-MTPA (δ_R) esters derived from alcohol **7**. The chemical shift differences for the ^{1}H atoms on the left side of the stereocenter exhibit negative values; those on the right side exhibit positive differences respectively. This analysis indicates the desired (*S*)-configuration of the C-5-OH stereocenter.

(3) The relative configuration at C-4 was determined by ^{1}H NMR analysis of the lactone formed by hydrogenation/cyclization (see below). The observed coupling constants and NOE interactions support the conformation and configuration shown below.

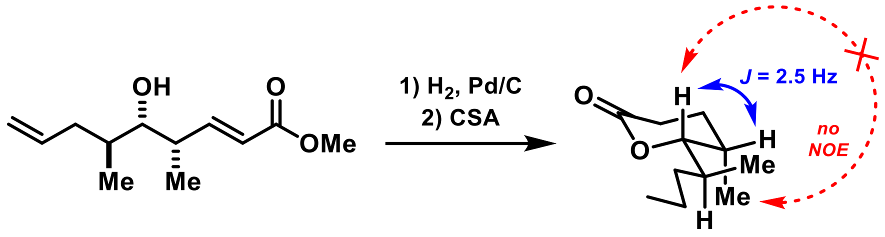

(5*S*,6*S*)-5-Methyl-6-((*S*)-pentan-2-yl)tetrahydro-2*H*-pyran-2-one (S4). A suspension of

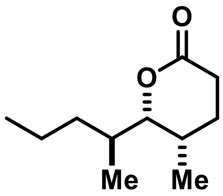

alcohol **7** (7.8 mg, 37 µmol) and Pd/C (5.2 mg) in ethyl acetate (1.0 mL) was stirred at room temperature for 2 h under hydrogen atmosphere. The suspension was filtered through Celite® and the solvent was removed under reduced pressure. The residue was dissolved in dichloromethane (1.0 mL) and camphor-10-sulfonic acid (2.0 mg, 8.6 µmol) was added. After stirring for 15.5 h, saturated aqueous sodium bicarbonate solution (1.0 mL) was introduced, the layers were separated and the aqueous phase was extracted with dichloromethane (3 × 1 mL). The combined organic layers were dried over anhydrous magnesium sulfate, the drying agent was filtered off, and the filtrate was concentrated under reduced pressure to give the title lactone as a colorless oil (6.2 mg, 92% yield). $[\alpha]_D^{20}$ = −54.3 (*c* 0.78, $CHCl_3$); **^{1}H NMR (600 MHz, $CDCl_3$):** δ 3.88 (dd, *J* = 10.1, 2.5 Hz, 1H), 2.52 (dd, *J* = 8.7, 6.3 Hz, 2H), 2.21 – 2.12 (m, 1H), 2.05 (dtd, *J* = 13.7, 8.7, 5.9 Hz, 1H), 1.82 (dddd, *J* = 13.6, 11.1, 5.6, 3.0 Hz, 1H), 1.74 – 1.68 (m, 1H), 1.66 (dtd, *J* = 13.7, 6.3, 3.0 Hz, 1H), 1.48 – 1.38 (m, 1H), 1.29 – 1.19 (m, 1H), 1.12 – 1.06 (m, 1H), 0.94 (d, *J* = 7.1 Hz, 3H), 0.91 (t, *J* = 7.3 Hz, 3H), 0.86 (d, *J* = 6.8 Hz, 3H). **^{13}C{^{1}H} NMR (151 MHz, $CDCl_3$):** δ 172.3, 86.7, 34.9, 34.5, 26.8, 26.7, 26.2, 19.6, 14.5, 14.3, 11.5. **IR (film):** 2959, 2932, 2873, 1734, 1457, 1383, 1325, 1238, 1201, 1126, 1062, 995, 980, 907, 738, 551 cm^{-1}. **HRMS-ESI *m/z*:** $[M+Na]^+$ calcd for $C_{11}H_{20}O_2Na^+$ 207.1355, found 207.1357.

Attempted Synthesis of Aldgamycin N

Methyl (4*S*,5*S*,6*S*,*E*)-5-((4-methoxybenzyl)oxy)-4,6-dimethylnona-2,8-dienoate (S5). In a 50-mL Schlenk tube, two drops of trifluoromethanesulfonic acid were added to a solution of alcohol **7** (1.19 g, 5.61 mmol) and 2-(4-methoxybenzyloxy)-4-methylquinoline (**10**) (3.16 g, 11.3 mmol) in dichloromethane (30 mL) at −20 °C. After 5 min, the cooling bath was removed and stirring continued at ambient temperature for 67 h. Triethylamine (0.03 mL) was added to the white suspension, which was filtered through a pad Celite®, carefully rinsing with dichloromethane (5 × 15 mL). The combined filtrates were concentrated under reduced pressure and the crude product was loaded onto Celite®. Purification by flash chromatography (hexanes/EtOAc, 25:1) furnished the title compound as a colorless oil (1.26 g, 68% yield). $[\alpha]_D^{20}$ = +11.3 (*c* 1.3, $CHCl_3$). **^{1}H NMR (400 MHz, $CDCl_3$):** δ 7.31 – 7.32 (m, 2H), 7.04 (dd, *J* = 15.8, 7.9 Hz, 1H), 6.90 – 6.83 (m, 2H), 5.86 (dd, *J* = 15.8, 1.3 Hz, 1H), 5.75 (dddd, *J* = 16.6, 10.3, 8.0, 6.2 Hz, 1H), 5.05 – 4.97 (m, 2H), 4.46 (s, 2H), 3.80 (s, 3H), 3.74 (s, 3H), 3.17 (dd, *J* = 6.3, 5.0 Hz, 1H), 2.71 – 2.59 (m, 1H), 2.43 – 2.34 (m, 1H), 1.91 (dddt, *J* = 13.7, 9.2, 8.0, 1.0 Hz, 1H), 1.83 – 1.71 (m, 1H), 1.12 (d, *J* = 6.7 Hz, 3H), 0.92 (d, *J* = 6.8 Hz, 3H). **^{13}C{^{1}H} NMR (101 MHz, $CDCl_3$):** δ 167.3, 159.3, 153.1, 137.6, 130.8, 129.5, 120.2, 116.2, 113.9, 86.4, 74.7, 55.4, 51.6, 39.5, 36.7, 36.2, 16.7, 14.2. **IR (film)** 3072, 2964, 2935, 2910, 2875, 2838, 1722, 1655, 1613, 1586, 1514, 1461, 1436, 1339, 1318, 1301, 1248, 1193, 1175, 1109, 1063, 1035, 1012, 990, 958, 913, 851, 823, 759, 731 cm^{-1}. **HRMS-ESI *m/z*:** $[M+Na]^+$ calcd for $C_{20}H_{28}O_4Na$ 355.1880; found 355.1881.

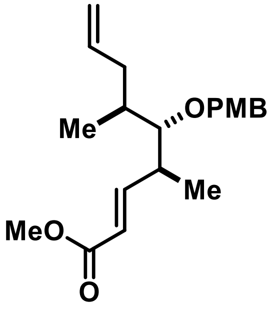

Methyl (4*S*,5*S*,6*S*,*E*)-5-((4-methoxybenzyl)oxy)-4,6-dimethyl-8-oxonon-2-enoate (8). In a 100-mL two-necked flask, oxygen gas was bubbled via a syringe needle through a mixture of THF and water (1:1, 40 mL) for 10 min. Copper(I) chloride (1.06 g, 10.7 mmol) and palladium(II) chloride (190 mg, 1.07 mmol) were added, followed by a solution of alkene **S5** (1.78 g, 5.35 mmol) in THF/water (1:1, 20 mL). The resulting greenish brown mixture was vigorously stirred under oxygen atmosphere (balloon) at room temperature. After 5 min, the mixture turned black and eventually became a green suspension, when TLC analysis (hexanes/EtOAc, 8:1) indicated full conversion of the starting material. The suspension was filtered through a pad of Celite®, which was rinsed with *tert*-butyl methyl ether (5 × 10 mL). The combined filtrates were washed with saturated aqueous ammonium chloride solution (80 mL) and the aqueous phase was extracted with *tert*-butyl methyl ether (6 × 20 mL). The combined organic layers were dried over anhydrous sodium sulfate, the drying agent was filtered off, and the solvent was removed under reduced pressure. Purification

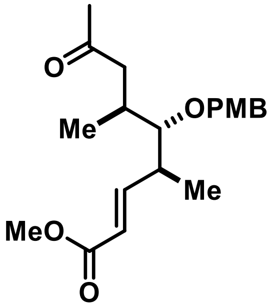

of the residue by flash chromatography (hexanes/EtOAc, 7:2) furnished the title compound as a colorless oil (1.64 g, 88% yield) $[\alpha]_D^{20}$ = +12.0 (*c* 0.85, $CHCl_3$). **^{1}H NMR (400 MHz, $CDCl_3$):** δ 7.25 – 7.21 (m, 2H), 7.03 (dd, *J* = 15.8, 7.7 Hz, 1H), 6.90 – 6.85 (m, 2H), 5.85 (dd, *J* = 15.8, 1.4 Hz, 1H), 4.50 – 4.37 (m, 2H), 3.80 (s, 3H), 3.74 (s, 3H), 3.17 (t, *J* = 5.4 Hz, 1H), 2.68 – 2.55 (m, 2H), 2.32 – 2.19 (m, 2H), 2.06 (s, 3H), 1.13 (d, *J* = 6.7 Hz, 3H), 0.95 (d, *J* = 6.4 Hz, 3H). **^{13}C{^{1}H} NMR (101 MHz, $CDCl_3$):** δ 208.5, 167.1, 159.4, 152.3, 130.5, 129.6, 120.5, 114.0, 86.3, 74.5, 55.4, 51.7, 46.8, 39.4, 32.5, 30.4, 18.1, 14.3. **IR (film):** 2968, 2878, 2839, 1718, 1654, 1613, 1587, 1514, 1458, 1435, 1337, 1300, 1274, 1249, 1194, 1176, 1081, 1034, 992, 929, 866, 849, 821 cm^{-1}. **HRMS-ESI *m/z*:** $[M+Na]^+$ calcd for $C_{20}H_{28}O_5Na$ 371.1829; found 371.1832.

Addition products 18 and 8-*epi*-18. In a 100-mL two-necked flask, *n*-BuLi (1.6 M in hexanes, 4.9 mL, 7.8 mmol) was added over 10 min to a solution of alkyne **17** (4.06 g, 8.01 mmol) in THF (55 mL) at –50 °C and stirring was continued at this temperature for 2 h.

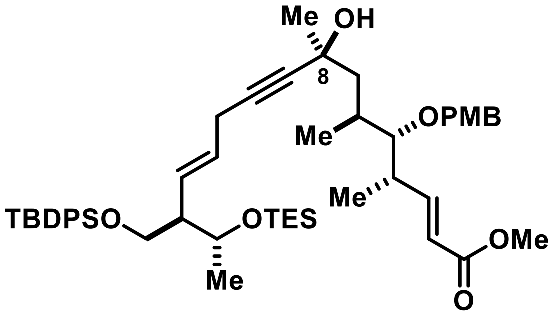

In a separate 250-mL two-necked flask, a solution of ketone **8** (1.64 g, 4.71 mmol) in THF (20 mL) was added to lanthanum(III) chloride bis(lithium chloride) complex (0.6 M in THF, 13 mL, 7.8 mmol) and the resulting mixture was stirred for 1 h at room temperature.

Both flasks were cooled to –78 °C and the solution of the lithium acetylide was transferred to the second flask containing the lanthanide chloride adduct via cannula. Stirring was continued for 1 h at this temperature before the mixture was diluted with *tert*-butyl methyl ether (50 mL) and washed with saturated aqueous ammonium chloride solution (80 mL). Both layers were carefully separated and the aqeous layer was filtered through a pad of Celite® to remove a white precipitate, which was carefully rinsed with *tert*-butyl methyl ether (5 × 15 mL). The aqueous phase was extracted with *tert*-butyl methyl ether (5 × 25 mL), all organic layers were combined and dried over anhydrous sodium sulfate. The drying agent was filtered off, and the solvent was removed under reduced pressure. Purification of the residue by flash chromatography (hexanes/EtOAc, 20:1 → 12:1 → 6:1 → 4:1) furnished unreacted alkyne **17** (1.31 g, 2.58 mmol) and the title compound as a mixture of diastereomers (3.34 g, 83% yield, d. r. = 1:1).

The C-8 epimers (3.34 g, 12 mL MeCN) were separated by preparative HPLC (stationary phase: Kromasil 100-5-C18, 2 × 150 mm length × 30 mm diameter, 5 µm (two columns used in series), eluent: MeCN/MeOH (95:5, v/v), 42.5 mL/min (6.5 MPa, 308 K, UV detection at 254 nm)). The desired 8-(*S*) epimer was eluted first (t_R = 22.0 min), then the undesired 8-(*R*) epimer (t_R = 24.5 min).

*Analytical and spectral data of the desired isomer **(8S)-18***: colorless gum (1.00 g, 25% yield); $[\alpha]_D^{20}$ = +10.0 (*c* 0.62, $CHCl_3$). **^{1}H NMR (400 MHz, $CDCl_3$):** δ 7.69 – 7.60 (m, 4H), 7.45 – 7.32 (m, 6H),

7.27 – 7.22 (m, 2H), 7.01 (dd, *J* = 15.7, 7.9 Hz, 1H), 6.89 – 6.82 (m, 2H), 5.85 (dd, *J* = 15.8, 1.3 Hz, 1H), 5.60 (ddt, *J* = 15.4, 9.2, 1.7 Hz, 1H), 5.36 (dt, *J* = 15.5, 5.7 Hz, 1H), 4.53 – 4.43 (m, 2H), 4.12 (qd, *J* = 6.3, 3.4 Hz, 1H), 3.78 (s, 3H), 3.76 – 3.71 (m, 4H), 3.54 (dd, *J* = 9.9, 6.2 Hz, 1H), 3.13 (t, *J* = 5.6 Hz, 1H), 2.96 – 2.88 (m, 2H), 2.69 (s, 1H), 2.68 – 2.58 (m, 1H), 2.12 (dtd, *J* = 9.7, 6.5, 3.3 Hz, 1H), 2.07 – 1.97 (m, 1H), 1.91 (dd, *J* = 14.4, 3.2 Hz, 1H), 1.53 (dd, *J* = 14.4, 6.9 Hz, 1H), 1.41 (s, 3H), 1.103 (d, *J* = 6.7 Hz, 3H), 1.100 (d, *J* = 7.0 Hz, 3H), 1.07 (d, *J* = 6.3 Hz, 3H), 1.04 (s, 9H), 0.92 (t, *J* = 7.9 Hz, 9H), 0.62 – 0.49 (m, 6H). **$^{13}C\{^1H\}$ NMR (101 MHz, $CDCl_3$):** δ 167.2, 159.4, 152.7, 135.77, 135.74, 134.13, 134.10, 130.3, 129.71, 129.68, 129.64, 129.5, 129.4, 127.7, 127.3, 120.4, 113.9, 87.7, 86.6, 81.1, 74.8, 67.8, 67.3, 64.9, 55.4, 53.0, 51.6, 45.8, 39.5, 33.2, 31.1, 27.1, 22.3, 22.2, 19.8, 19.4, 14.7, 7.11, 5.24. **IR (film):** 3480, 3070, 3050, 2957, 2932, 2876, 1724, 1656, 1613, 1588, 1514, 1462, 1428, 1374, 1335, 1301, 1248, 1193, 1175, 1152, 1111, 1080, 1036, 1011, 974, 824, 741, 703, 614, 567, 505, 492 cm^{-1}. **HRMS-ESI *m/z*:** $[M+Na]^+$ calcd for $C_{51}H_{74}O_7Si_2Na$ 877.4865; found 877.4857.

Analytical and spectral data of the undesired 8-(R) epimer **8-*epi*-18***:* colorless gum (885 mg, 22% yield) $[\alpha]_D^{20}$ = +28.5 (*c* 1.3, $CHCl_3$). **^1H NMR (400 MHz, $CDCl_3$):** δ 7.67 – 7.7.61 (m, 4H), 7.44 – 7.32 (m, 6H), 7.25 – 7.21 (m, 2H), 7.09 (dd, *J* = 15.8, 7.3 Hz, 1H), 6.88 – 6.83 (m, 2H), 5.86 (dd, *J* = 15.8, 1.4 Hz, 1H), 5.57 (ddt, *J* = 15.4, 9.1, 1.7 Hz, 1H), 5.35 (dt, *J* = 15.5, 5.5 Hz, 1H), 4.50 – 4.42 (m, 2H), 4.11 (qd, *J* = 6.3, 3.3 Hz, 1H), 3.79 (s, 3H), 3.77 – 3.72 (m, 4H), 3.55 (s, 1H), 3.53 (dd, *J* = 6.2, 10.0 Hz, 1H), 3.16 (dd, *J* = 7.3, 4.1 Hz, 1H), 2.95 – 2.87 (m, 2H), 2.68 – 2.57 (m, 1H), 2.20 – 2.07 (m, 2H), 1.76 (dd, *J* = 14.5, 4.5 Hz, 1H), 1.48 (dd, *J* = 14.5, 4.7 Hz, 1H), 1.41 (s, 3H), 1.10 (d, *J* = 6.8 Hz, 3H), 1.06 (d, *J* = 6.3 Hz, 3H), 1.04 (s, 9H), 1.01 (d, *J* = 6.9 Hz, 3H), 0.91 (t, *J* = 7.9 Hz, 9H), 0.63 – 0.47 (m, 6H). **$^{13}C\{^1H\}$ NMR (101 MHz, $CDCl_3$):** δ 167.3, 159.5, 153.3, 135.78, 135.74, 134.1, 130.0, 129.8, 129.7, 129.6, 129.3, 127.7, 127.6, 120.3, 113.9, 87.2, 86.1, 80.8, 74.4, 67.6, 67.3, 64.9, 55.4, 53.0, 51.7, 47.6, 41.5, 39.3, 33.3, 32.0, 29.2, 27.8, 27.1, 22.8, 22.4, 22.2, 20.6, 20.5, 19.6, 19.4, 14.5, 13.3, 7.11, 5.23. **IR (film):** 3435, 3071, 3051, 2956, 2932, 2876, 1724, 1655, 1613, 1588, 1514, 1462, 1428, 1373, 1321, 1301, 1249, 1176, 1151, 1111, 1078, 1036, 1011, 974, 824, 740, 703, 612, 556, 505 cm^{-1}. **HRMS-ESI *m/z*:** $[M+Na]^+$ calcd for $C_{51}H_{74}O_7Si_2Na$ 877.4865; found 877.4859.

Macrocyclization precursor 19. In a 10-mL round-bottom flask, pyridinium *p*-toluenesulfonate (18.5 mg, 73.6 µmol) was added to a solution of silyl ether **18** (523 mg, 0.612 mmol) in ethanol (10 mL) and THF (1.1 mL) at 0 °C and the resulting solution was stirred for 6.5 h at this temperature. The mixture was diluted with ethyl acetate (20 mL) and washed with half-saturated aqueous sodium bicarbonate solution (20 mL).

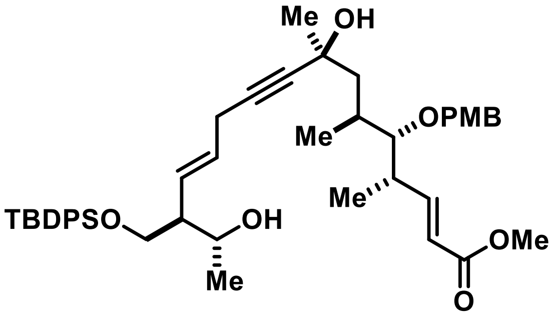

The aqueous phase was extracted with ethyl acetate (6 × 10 mL), the combined organic layers were dried over anhydrous sodium sulfate, the drying agent was filtered off, and the solvent was removed under reduced pressure. Purification of the residue by flash chromatography (hexanes/EtOAc, 3:1 → 2:1) furnished the title compound as a colorless gum (402 mg, 89% yield). $[\alpha]_D^{20}$ = +7.5 (*c* 0.05, $CHCl_3$). **^{1}H NMR (400 MHz, $CDCl_3$):** δ 7.68 – 7.62 (m, 4H), 7.47 – 7.35 (m, 6H), 7.26 – 7.22 (m, 2H), 7.01 (dd, *J* = 15.8, 7.9 Hz, 1H), 6.89 – 6.82 (m, 2H), 5.85 (dd, *J* = 15.7, 1.2 Hz, 1H), 5.71 (ddt, *J* = 15.4, 9.1, 1.7 Hz, 1H), 5.46 (dtd, *J* = 15.5, 5.5, 0.8 Hz, 1H), 4.52 – 4.44 (m, 2H), 4.07 (dtd, *J* = 10.2, 6.4, 3.9 Hz, 1H), 3.81 – 3.70 (m, 8H), 3.13 (t, *J* = 5.6 Hz, 1H), 2.98 – 2.92 (m, 2H), 2.71 (s, 1H), 2.69 – 2.59 (m, 2H), 2.24 (dq, *J* = 9.3, 4.8 Hz, 1H), 2.08 – 1.98 (m, 1H), 1.91 (dd, *J* = 14.4, 3.3 Hz, 1H), 1.53 (dd, *J* = 14.4, 7.0 Hz, 1H), 1.42 (s, 3H), 1.15 (d, *J* = 6.4 Hz, 3H), 1.12 – 1.08 (m, 6H), 1.05 (s, 9H). **^{13}C{^{1}H} NMR (101 MHz, $CDCl_3$):** δ 167.2, 159.4, 152.7, 135.78, 135.72, 133.18, 133.13, 130.30, 129.98, 129.7, 128.5, 128.4, 127.9, 120.4, 113.9, 87.7, 86.9, 80.7, 74.8, 68.9, 67.8, 66.5, 55.4, 51.6, 50.8, 45.7, 39.5, 33.2, 31.2, 27.0, 22.4, 20.6, 19.8, 19.3, 14.7. **IR (film):** 3460, 2961, 2880, 1721, 1647, 1612, 1514, 1464, 1428, 1299, 1249, 1176, 1111, 1087, 1037, 1009, 850, 824, 740, 704, 617, 505, 490 cm^{-1}. **HRMS-ESI *m/z*:** $[M+Na]^+$ calcd for $C_{45}H_{60}O_7SiNa$ 763.4001; found 763.4002.

(8*R*)-Configured Macrocyclization Precursor 8-*epi*-19. Prepared analogously from **8-*epi*-18** as a colorless gum (202 mg, 77% yield). $[\alpha]_D^{20}$ = +24.9 (*c* 0.92, $CHCl_3$). **^{1}H NMR (400 MHz, $CDCl_3$):** δ 7.68 – 7.62 (m, 4H), 7.47 – 7.35 (m, 6H), 7.26 – 7.20 (m, 2H), 7.09 (dd, *J* = 15.7, 7.3 Hz, 1H), 6.89 – 6.82 (m, 2H), 5.87 (dd, *J* = 15.8, 1.5 Hz, 1H), 5.69 (ddt, *J* = 15.4, 9.0, 1.7 Hz, 1H), 5.45 (dtd, *J* = 15.4, 5.5, 0.8 Hz, 1H), 4.51 – 4.42 (m, 2H), 4.07 (qd, *J* = 6.4, 3.6 Hz, 1H), 3.82 – 3.69 (m, 9H), 3.16 (dd, *J* = 7.4, 4.4 Hz, 1H), 2.98 – 2.91 (m, 2H), 2.69 – 2.57 (m, 1H), 2.29 – 2.20 (m, 1H), 2.20 – 2.09 (m, 1H), 1.76 (dd, *J* = 14.5, 4.5 Hz, 1H), 1.49 (dd, *J* = 14.5, 4.5 Hz, 1H), 1.41 (s, 3H), 1.15 (d, *J* = 6.5 Hz, 3H), 1.09 (d, *J* = 6.8 Hz, 3H), 1.05 (s, 9H), 1.01 (d, *J* = 7.0 Hz, 3H). **^{13}C{^{1}H} NMR (101 MHz, $CDCl_3$):** δ 167.3, 159.5, 153.2, 135.8, 135.7, 133.2, 133.1, 130.03, 129.98, 129.96, 129.7, 128.6, 128.2, 127.9, 120.3, 113.9, 87.2, 86.5, 80.2, 74.5, 68.9, 67.6, 66.5, 55.4, 51.7, 50.7, 47.7, 39.2, 33.3, 32.0, 27.0, 22.4, 20.60, 20.56, 19.3, 13.2. **IR (film):** 3434, 3071, 3047, 2961, 2931, 2859, 1721, 1654, 1613, 1588, 1514, 1461, 1428, 1363, 1300, 1249, 1194, 1177, 1146, 1112, 1086, 1036, 941, 823, 742, 704, 613, 506 cm^{-1}. **HRMS-ESI *m/z*:** $[M+Na]^+$ calcd for $C_{45}H_{60}O_7SiNa$ 763.4001; found 730.4012.

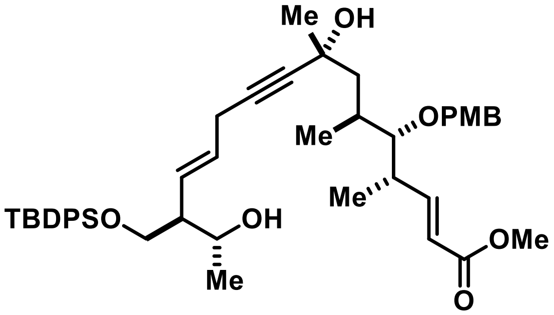

Macrolactone 20. In a 500-mL round-bottomed flask equipped with a reflux condenser, distannoxane **25a**

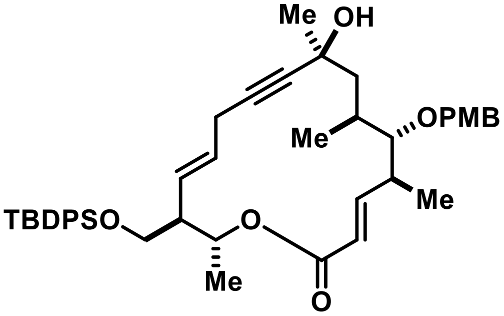

(457 mg, 0.410 mmol) was added to a solution of hydroxy ester **19** (402 mg, 0.543 mmol) in toluene (300 mL) and the resulting solution was stirred at reflux temperature for 5 d. After reaching room temperature, the solvent was evaporated under reduced pressure and the residue was purified by flash chromatography on silica gel (15 – 40 µm, hexanes/EtOAc, 5:1 → 4:1) to furnish the title compound as a pale yellow gum (260 mg, 68% yield). $[\alpha]_D^{20}$ = +62.7 (*c* 0.07, $CHCl_3$). **^{1}H NMR (400 MHz, $CDCl_3$):** δ 7.66 – 7.59 (m, 4H), 7.45 – 7.33 (m, 6H), 7.30 – 7.25 (m, 2H), 6.90 – 6.85 (m, 2H), 6.76 (dd, *J* = 15.6, 9.5 Hz, 1H), 5.75 (dd, *J* = 15.7, 0.8 Hz, 1H), 5.58 (dd, *J* = 15.3, 9.4 Hz, 1H), 5.39 (ddd, *J* = 15.2, 7.8, 4.1 Hz, 1H), 5.23 (dq, *J* = 7.7, 6.3 Hz, 1H), 4.61 (d, *J* = 10.6 Hz, 1H), 4.51 (d, *J* = 10.6 Hz, 1H), 3.80 (s, 3H), 3.70 – 3.59 (m, 2H), 3.10 (dd, *J* = 9.8, 1.9 Hz, 1H), 2.93 (ddd, *J* = 18.2, 4.1, 1.9 Hz, 1H), 2.83 (ddd, *J* = 18.3, 7.9, 1.2 Hz, 1H), 2.58 (tdd, *J* = 9.6, 6.4, 3.1 Hz, 1H), 2.29 – 2.19 (m, 1H), 2.19 – 2.09 (m, 1H), 1.74 (dd, *J* = 14.5, 2.8 Hz, 1H), 1.69 (s, 1H), 1.59 (dd, *J* = 14.5, 10.5 Hz, 1H), 1.49 (s, 3H), 1.21 (d, *J* = 6.3 Hz, 3H), 1.20 (d, *J* = 6.8 Hz, 3H), 1.15 (d, *J* = 6.6 Hz, 3H), 1.05 (s, 9H). **^{13}C{^{1}H} NMR (101 MHz, $CDCl_3$):** δ 166.1, 159.4, 150.5, 135.8, 133.61, 133.56, 130.9, 129.9, 129.8, 129.7, 129.6, 127.9, 127.83, 127.81, 121.4, 114.0, 89.1, 81.9, 75.4, 69.5, 68.9, 64.4, 55.4, 52.0, 42.6, 40.8, 34.2, 33.1, 27.0, 22.6, 19.7, 19.4, 18.8, 18.1. **IR (film):** 3496, 3071, 3048, 2959, 2930, 2858, 1715, 1654, 1613, 1587, 1514, 1462, 1428, 1361, 1327, 1302, 1248, 1180, 1111, 1083, 1036, 986, 823, 741, 703, 610, 504 cm^{-1}. **HRMS-ESI *m/z*:** $[M+Na]^+$ calcd for $C_{44}H_{56}O_6SiNa$ 731.3738; found 731.3743.

(8*R*)-Configured Macrolactone 8-*epi*-20. Prepared analogously from **8-*epi*-19** as a colorless

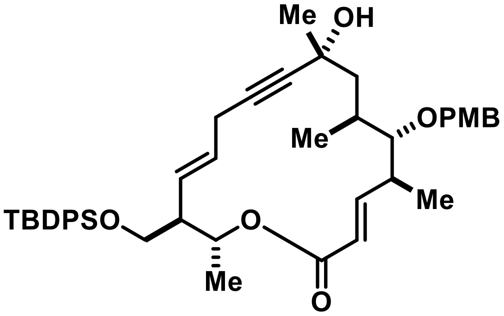

gum (113 mg, 69% yield). $[\alpha]_D^{20}$ = +12.6 (*c* 1.2, $CHCl_3$). **^{1}H NMR (400 MHz, $CDCl_3$):** δ 7.65 – 7.60 (m, 4H), 7.44 – 7.32 (m, 6H), 7.29 – 7.25 (m, 2H), 6.98 (dd, *J* = 16.0, 6.8 Hz, 1H), 6.91 – 6.86 (m, 2H), 5.80 (dd, *J* = 15.9, 1.5 Hz, 1H), 5.66 (ddt, *J* = 15.4, 9.2, 1.7 Hz, 1H), 5.46 (ddd, 15.3, 6.3, 4.0 Hz, 1H), 5.28 (quint, *J* = 6.2 Hz, 1H), 4.59 – 4.48 (m, 2H), 3.80 (s, 3H), 3.70 (br s, 1H), 3.64 – 3.52 (m, 2H), 3.02 – 2.92 (m, 2H), 2.87 – 2.73 (m, 2H), 2.37 – 2.28 (m, 1H), 2.27 – 2.17 (m, 1H), 1.70 (dd, *J* = 14.4, 4.6 Hz, 1H), 1.53 (dd, *J* = 14.4, 5.4 Hz, 1H), 1.44 (s, 3H), 1.24 (d, *J* = 6.3 Hz, 3H), 1.15 (d, *J* = 7.0 Hz, 3H), 1.04 (s, 9H), 0.90 (d, *J* = 7.0 Hz, 3H). **^{13}C{^{1}H} NMR (101 MHz, $CDCl_3$):** δ 165.9, 159.6, 149.3, 135.80, 135.76, 133.7, 133.6, 130.1, 129.78, 129.75, 129.6, 128.5, 128.2, 127.79, 127.77, 122.0, 114.0, 87.9, 80.9, 72.6, 69.6, 68.2, 64.6, 55.4, 51.3, 48.3, 38.7, 33.8, 31.1, 27.0, 22.4, 19.8, 19.4, 19.0, 17.9. **IR (film):** 3406,

3071, 3046, 2959, 2931, 2858, 1714, 1655, 1612, 1587, 1514, 1462, 1428, 1363, 1302, 1249, 1180, 1111, 1086, 1037, 986, 823, 742, 704, 610, 504 cm^{-1}. **HRMS-ESI *m/z*:** $[M+Na]^+$ calcd for $C_{44}H_{56}O_6SiNa$ 731.3738; found 731.3747.

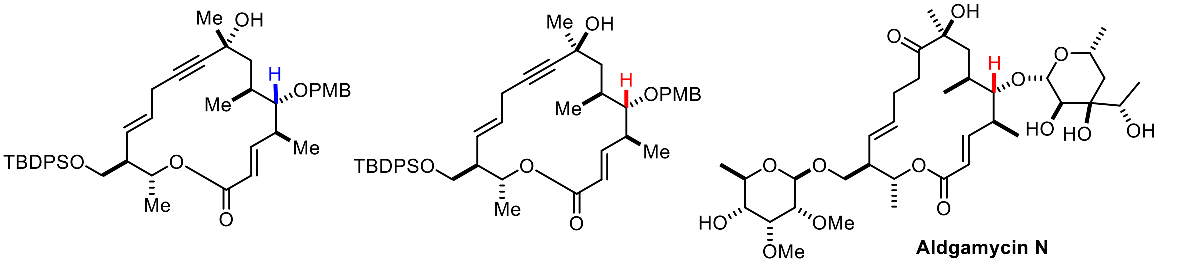

Aldgamycin N

8-*epi*-**20**: H5: ≈ 6, 5 Hz   **20**: H5: 9.8, 1.9 Hz   H5, lit.: br d, 9.0 Hz

The configuration at C8 was tentatively assigned at this point by comparison of the ^{1}*H NMR signals at the C5 position for both epimers with the reported literature value of natural Aldgamycin N (3.38 ppm, br d, 9.0 Hz).*[8] *Whereas* ***20*** *exhibits for H-5 a doublet of doublet with a similarly large coupling (3.10 ppm, J = 9.8, 1.9 Hz) as in the natural product,* ***8-epi-20*** *exhibits for H-5 a broad triplet (2.99 ppm, br t, J ~ 6, 5 Hz). The accompanying paper confirms this assigment by synthesis of the natural product Aldgamycin N from* ***20****.*[9]

Alkenylstannane 21. In a 10-mL Schlenk tube, a solution of $[Cp^*RuCl]_4$ in dichloromethane (75 mg/mL, 0.20 mL, 14 µmol) was added to propargylic alcohol **20** (81.9 mg, 116 µmol) at room temperature, which caused the very dark green color of the catalyst solution to turn dark brown. A solution of tributyltin hydride in dichloromethane (0.35 M, 0.38 mL, 0.13 mmol) was then added over 1.5 h at room temperature. After complete addition, the dark red/brown solution was stirred for another 20 min before it was concentrated under reduced pressure. The residue was purified by flash chromatography, eluting with hexanes and hexanes/EtOAc (10:1) to give the title compound as a colorless gum (83.0 mg, 72% yield).

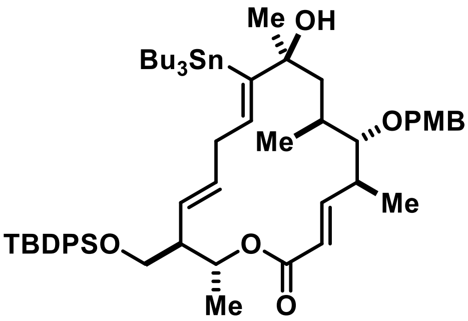

^{1}H NMR (400 MHz, $CDCl_3$): δ 7.67 – 7.61 (m, 4H), 7.45 – 7.32 (m, 6H), 7.30 – 7.25 (m, 2H), 6.90 – 6.85 (m, 2H), 6.73 (dd, *J* = 15.6, 9.5 Hz, 1H), 5.78 (dd, *J* = 15.6, 0.8 Hz, 1H), 5.71 (t, *J* = 6.8 Hz, 1H), 5.49 – 5.33 (m, 2H), 5.26 (quint, *J* = 6.3 Hz, 1H), 4.61 – 4.45 (m, 2H), 3.80 (s, 3H), 3.69 – 3.57 (m, 2H), 3.08 (dd, *J* = 9.5, 2.4 Hz, 1H), 2.78 – 2.53 (m, 3H), 2.29 – 2.20 (m, 1H), 2.07 – 1.95 (m, 1H), 1.83 – 1.73 (m, 1H), 1.50 – 1.38 (m, 7H), 1.32 – 1.24 (m, 6H), 1.24 – 1.20 (m, 6H), 1.12 (d, *J* = 6.7 Hz,

3H), 1.07 – 1.03 (m, 12H), 0.93 – 0.82 (m, 15H). **^{13}C{^{1}H} NMR (101 MHz, CDCl$_3$):** δ 166.0, 150.6, 135.8, 135.6, 132.4, 131.0, 129.8, 129.7, 129.6, 127.80, 127.78, 121.5, 113.9, 88.4, 78.4, 74.2, 69.6, 64.4, 55.4, 52.6, 40.6, 36.0, 32.6, 29.4, 27.6, 27.0, 22.8, 19.4, 18.9, 18.7, 18.1, 13.9, 12.6. **IR (film):** 3499, 2956, 2928, 2870, 2857, 1717, 1700, 1652, 1613, 1513, 1462, 1428, 1375, 1248, 1173, 1111, 1071, 1039, 981, 940, 863, 824, 740, 702, 614, 505 cm^{-1}. **HRMS-ESI *m/z*:** [M+Na]$^+$ calcd for $C_{56}H_{84}O_6SiSnNa$ 1023.4962; found 1023.4962.

Acyloin 22. In a 10-mL Schlenk tube, 4-(dimethylamino)pyridine (4.1 mg, 34 µmol) and copper(II) trifluoroacetate monohydrate (46.3 mg, 0.150 mmol) were added to a solution of alkenylstannane **21** (82.0 mg, 82.0 µmol) in DMSO (0.66 mL) at room temperature to give a green, homogeneous solution. The flask was immersed in a preheated oil bath at 45 – 50 °C and the mixture stirred at this temperature for 2 h. During this time, the color of the mixture became very dark green; eventually a fine, dark suspension was obtained. At this point, the mixture was diluted with *tert*-butyl methyl ether (10 mL) and washed with saturated aqueous ammonium chloride solution (10 mL). The aqueous phase was extracted with *tert*-butyl methyl ether (5 × 10 mL) and the organic layers were dried over anhydrous sodium sulfate. The drying agent was filtered off and the filtrate was concentrated under reduced pressure. The residue was purified by flash chromatography (hexanes/EtOAc, 8:1 → 4:1) to give the title compound as a colorless gum (49.5 mg, 83% yield). $[\alpha]_D^{20}$ = +18.6 (*c* 0.87, CHCl$_3$). **^{1}H NMR (400 MHz, CDCl$_3$):** δ 7.67 – 7.61 (m, 4H), 7.46 – 7.35 (m, 6H), 7.30 – 7.25 (m , 2H), 6.90 – 6.85 (m, 2H), 6.74 (dd, *J* = 15.5, 10.2 Hz, 1H), 5.80 (d, *J* = 15.5 Hz, 1H), 5.46 (dd, *J* = 15.2, 9.4 Hz, 1H), 5.39 – 5.30 (m, 1H), 5.24 (dq, *J* = 9.9, 6.3 Hz, 1H), 4.62 (d, *J* = 10.3 Hz, 1H), 4.49 (d, *J* = 10.3 Hz, 1H), 3.85 (s, 1H), 3.81 (s, 3H), 3.73 – 3.62 (m, 2H), 3.09 (dd, *J* = 10.2, 1.5 Hz, 1H), 2.71 – 2.55 (m, 2H), 2.36 – 2.24 (m, 1H), 2.23 – 2.09 (m, 3H), 1.96 – 1.86 (m, 1H), 1.84 – 1.77 (m, 1H), 1.49 – 1.37 (br m, 1H), 1.35 (s, 3H), 1.21 (d, *J* = 6.3 Hz, 3H), 1.15 (d, *J* = 6.6 Hz, 3H), 1.11 (d, *J* = 6.9 Hz, 3H), 1.07 (s, 9H). **^{13}C{^{1}H} NMR (101 MHz, CDCl$_3$):** δ 212.9, 165.8, 159.4, 151.4, 135.83, 135.80, 133.56, 133.53, 131.7, 130.6, 130.3, 129.9, 129.8, 129.7, 127.84, 127.82, 121.7, 114.0, 89.3, 79.2, 76.3, 69.7, 64.3, 55.5, 53.5, 41.2, 37.4, 34.9, 29.1, 26.5, 19.8, 19.5, 19.1, 18.9. **IR (film):** 3479, 2959, 2931, 2859, 1712, 1651, 1613, 1588, 1514, 1461, 1428, 1355, 1338, 1249, 1180, 1112, 1070, 1037, 993, 823, 741, 703, 610, 505, 493 cm^{-1}. **HRMS-ESI *m/z*:** [M+Na]$^+$ calcd for $C_{44}H_{58}O_7SiNa$ 749.3844; found 749.3849.

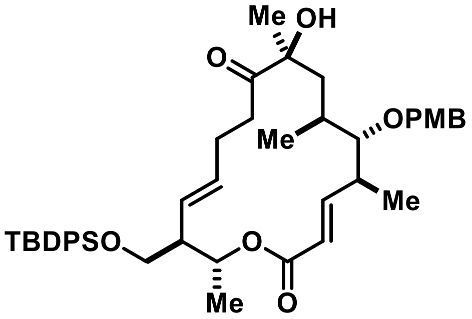

Attempted Liberation of the Aldgamycin N Aglycone

A. From PMB-Ether 22. In a pear-shaped flask, DDQ (9.9 mg, 44 μmol) was added in one portion to a vigorously stirred solution of compound **22** (18.4 mg, 25.3 μmol) in dichloromethane (0.25 mL) and water (0.05 mL) at 0 °C. After stirring for 1 h, the reaction mixture, which had turned from very dark green to brown, was diluted with *tert*-butyl methyl ether (10 mL) and washed with saturated aqueous sodium bicarbonate solution (10 mL). The aqueous phase was extracted with *tert*-butyl methyl ether (4 × 10 mL) and the organic layers were dried over anhydrous sodium sulfate. The drying agent was filtered off and the filtrate was concentrated under reduced pressure. Flash chromatography (hexanes/EtOAc, 5:1 → 2:1) furnished a mixture of ketone **23a** and hemiketals **23b** as a colorless gum (12.2 mg, 79% yield). Due to the complexity of this three-component mixture, the obtained material was only rudimentarily characterized. Characteristic C9 $^{13}C\{^{1}H\}$ NMR (101 MHz, $CDCl_3$) signals: δ 212.8 (ketone **23a**), 100.7 (hemiketals **23b**).

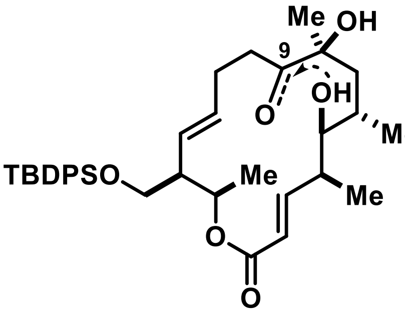

B. From the Analogous TBS-Ether S6

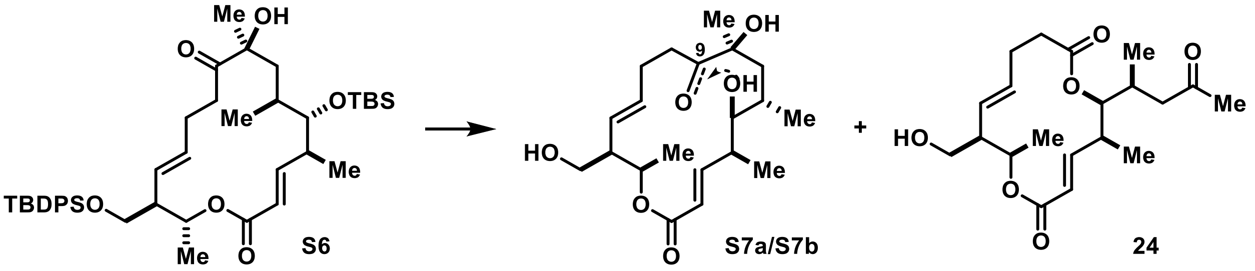

In a 4-mL Nalgene™ screw-capped reaction vessel, a solution of aqueous hydrogen fluoride (48 – 51% *w*/*w*) in acetonitrile (3:10 *v*/*v*, 1.45 mL) was added to silyl ether **S6** (52.0 mg, 72.1 μmol). The mixture was stirred for 21 h at room temperature before it was diluted with ethyl acetate (10 mL) and carefully poured onto saturated aqueous sodium bicarbonate solution (10 mL). The layers were separated and the aqueous phase was extracted with ethyl acetate (5 × 10 mL). The organic layers were dried over anhydrous sodium sulfate, the drying agent was filtered off, and the solvent was removed under reduced pressure. The residue was purified by flash chromatography (dichloromethane/methanol, 100:1 → 40:1). A fraction of macrodiolide **24** (5.4 mg, 20% yield) was eluted first, followed by a fraction containing a mixture of ketone **S7a** and hemiketals **S7b** (15.0 mg, ca. 56% yield), admixed with small amounts of unidentified impurities.

*Analytical and spectra data of macrodiolide **24***: colorless gum; $[\alpha]_D^{20}$ = –110.8 (*c* 0.13, $CHCl_3$). **^{1}H NMR (600 MHz, $CDCl_3$):** δ 6.79 (dd, *J* = 16.0, 4.3 Hz, 1H), 5.75 (dd, 16.0, 2.0 Hz, 1H), 5.55 (dtd, *J* = 15.8, 6.9, 1.0 Hz, 1H), 5.42 (ddt, *J* = 15.8, 7.7, 1.2 Hz, 1H), 5.01 (dq, *J* = 7.5, 6.4 Hz, 1H), 4.94 (dd, *J* = 10.5, 2.8 Hz, 1H), 3.60 (t, *J* = 6.2 Hz, 1H), 2.72 – 2.66 (m, 1H), 2.51 – 2.34 (m, 5H), 2.31 – 2.18 (m, 2H), 2.17 – 2.10 (m, 1H), 2.11 (s, 3H), 1.68 (t, *J* = 6.2 Hz, 1H), 1.34 (d, *J* = 6.4 Hz, 3H), 1.11 (d, *J* = 6.8 Hz, 3H), 0.95 (d, *J* = 6.7 Hz, 3H). **^{13}C{^{1}H} NMR (151 MHz, $CDCl_3$):** δ 207.7, 173.0, 165.8, 150.7, 132.5, 128.9, 122.1, 78.4, 69.4, 62.6, 51.1, 47.6, 36.4, 34.2, 30.7, 30.6, 27.0, 19.0, 16.4, 10.2. **IR (film):** 3489, 2974, 2940, 2884, 1710, 1642, 1457, 1417, 1360, 1273, 1196, 1166, 1103, 1051, 977, 914, 866, 732 cm^{-1}. **HRMS-ESI *m/z*:** $[M+Na]^+$ calcd for $C_{20}H_{30}O_6Na$ 389.1935; found 389.1934.

Total Synthesis of Mycinolide IV: Branch-Selective Asymmetric Hydroformylation

Methyl (*4S,5S,6S,E*)-5-(methoxymethoxy)-4,6-dimethylnona-2,8-dienoate (26). Phosphorus pentoxide (1.45 g, 5.11 mmol) was added in one portion to a solution of alcohol **7** (1.09 g, 5.12 mmol) and dimethoxymethane (4.1 mL, 46 mmol) in dichloromethane (46 mL) at room temperature, resulting in formation of a suspension with a rapidly darkening solid. After 16 h, the mixture was filtered through a pad of aluminum oxide (neutral), which was washed with 20% EtOAc in dichloromethane (100 mL). The combined filtrates were concentrated, giving a light yellow liquid. Purification by automated column chromatography (Biotage® 25 g SNAP Ultra HP-Sphere™ 25µm cartridge, loading as solution in hexanes; gradient of 8 − 66% EtOAc in hexanes over 15 column volumes) afforded the title compound as a colourless liquid (1.05 g, 87% yield, 87:13 d.r.). $[\alpha]_D^{20}$ = –16.0 (*c* 1.0, $CHCl_3$). **^{1}H NMR (400 MHz, $CDCl_3$):** δ 7.00 (dd, *J* = 15.8, 7.8 Hz, 1H), 5.84 (dd, *J* = 15.8, 1.3 Hz, 1H), 5.75 (dddd, *J* = 16.7, 10.3, 7.8, 6.3 Hz, 1H), 5.06 – 4.97 (m, 2H), 4.65 – 4.60 (m, 2H), 3.73 (s, 3H), 3.39 (s, 3H), 3.29 (t, *J* = 5.4 Hz, 1H), 2.68 – 2.58 (m, 1H), 2.36 – 2.28 (m, 1H), 1.91 – 1.82 (m, 1H), 1.80 – 1.68 (m, 1H), 1.10 (d, *J* = 6.7 Hz, 3H), 0.91 (d, *J* = 6.8 Hz, 3H). **^{13}C{^{1}H} NMR (101 MHz, $CDCl_3$):** δ 167.0, 152.4, 137.3, 120.2, 116.1, 98.1, 85.9, 56.1, 51.5, 39.1, 36.4, 35.8, 16.4, 14.4. **IR (film):** 2951, 2887, 1772, 1655, 1642, 1436, 1270, 1147, 1092, 1027, 990, 915, 728 cm^{-1}. **HRMS-ESI *m/z*:** $[M+Na]^+$ calcd for $C_{14}H_{24}O_4Na$ 279.1566; found 279.1567.

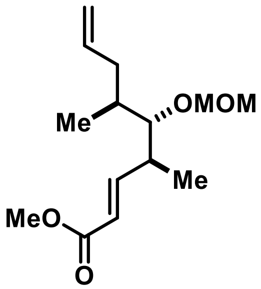

Methyl (*4S,5S,6S,8R,E*)-5-(methoxymethoxy)-4,6,8-trimethyl-9-oxonon-2-enoate (27).

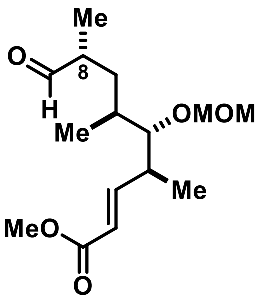

$Rh(acac)(CO)_2$ (34 mg, 132 µmol) and (R_{ax},*R*,*R*)-BOBPhos (**32**) (108 mg, 165 µmol) were added to toluene (previously degassed with argon; 3.5 mL) in a Schlenk tube, resulting in immediate gas evolution and the formation of a clear, yellow solution. This solution was transferred into an oven-dried (80 °C), argon-flushed autoclave equipped with a glass-inlet and stirring bar, pre-charged with hexafluorobenzene (140 mL). The autoclave was sealed, pressurized with hydrogen (7.5 bar) and carbon monoxide (7.5 bar) and heated to 50 °C (external temperature) while the mixture was vigorously stirred.

After 1 h, the autoclave was cooled to room temperature, overpressure was released, and a solution of olefin **26** (1.00 g, 3.92 mmol) in hexafluorobenzene (5 mL) was added to the catalyst solution in an argon counter-flow. The autoclave was again sealed, pressurized with hydrogen/carbon monoxide (7.5 bar each) and warmed to 31 °C (internal temperature after equilibration) while the mixture was vigorously stirred. After 3 d, the overpressure was released, and the crude mixture was directly subjected to flash chromatography on silica gel (15 − 40 µm particle size, gradient of 1 – 10% EtOAc in hexanes) affording aldehyde **27** as a yellow liquid (675 mg, 60% yield, **27**/8-*epi*-**27** = 96:4, d.r. (27:Σ of all other diastereomers) = 83:17). $[\alpha]_D^{20}$ = –31.1 (*c* 0.99, $CHCl_3$). **^{1}H NMR (400 MHz, $CDCl_3$):** δ 9.52 (d, *J* = 2.8 Hz, 1H), 6.95 (dd, *J* = 15.7, 8.0 Hz, 1H), 5.85 (dd, *J* = 15.7, 1.3 Hz, 1H), 4.65 – 4.61 (m, 2H), 3.73 (s, 3H), 3.39 (s, 3H), 3.25 (dd, *J* = 6.0, 4.6 Hz, 1H), 2.69 – 2.58 (m, 1H), 2.51 – 2.38 (m, 1H), 1.94 (ddd, *J* = 13.7, 9.9, 3.4 Hz, 1H), 1.75 – 1.62 (m, 1H), 1.22 – 1.13 (m, 1H), 1.10 (d, 4.2 Hz, 3H), 1.09 (d, *J* = 3.9 Hz, 3H), 0.95 (d, *J* = 6.9 Hz, 3H). **^{13}C{^{1}H} NMR (101 MHz, $CDCl_3$):** δ 205.0, 166.9, 151.8, 120.5, 98.1, 86.5, 56.1, 51.5, 44.1, 39.4, 33.6, 33.1, 17.3, 15.0, 14.9. **IR (film):** 2964, 2935, 2881, 1720, 1665, 1459, 1436, 1273, 1193, 1177, 1147, 1093, 1029, 988, 919, 864, 753 cm^{-1}. **HRMS-ESI *m/z*:** $[M+Na]^+$ calcd for $C_{15}H_{26}O_5Na$ 309.1672; found 309.1675.

Stereochemical Analysis of the Hydroformylation Product

The analogous reaction using the enantiomeric ligand *ent*-**32** gave 8-*epi*-**27** as the major product (see below); **27** and 8-*epi*-**27** were then transformed into the corresponding lactones **33** and 8-*epi*-**33**; the NMR data of these samples could be compared with those of authentic **33** reported in the literature.[10]

As shown in Table S2, an excellent match with the reported ^{1}H NMR data was found for the lactone obtained from aldehyde **27**, which was formed with the aid of (R_{ax},*R*,*R*)-BOBPhos (**32**) as the chiral ligand.

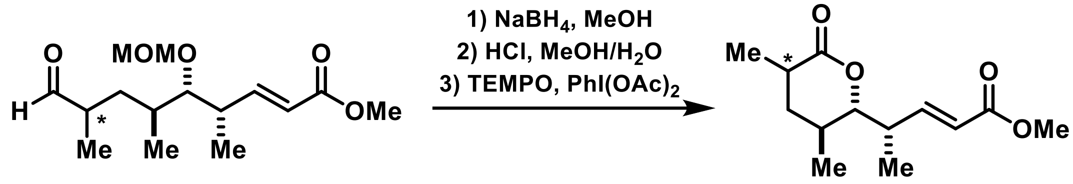

**33 (literature data)**				**33 (obtained from aldehyde 27)**							
**δ (ppm)**		***J* (Hz)**		**δ (ppm)**		***J* (Hz)**			**Δδ (ppm)**	**Δ*J* (Hz)**	
7.08	dd	15.8	7.2	7.07	dd	15.8	7.4		-0.01	0.0	0.2
5.88	dd	15.8	1.3	5.88	dd	15.8	1.4		0.00	0.0	0.1
4.04	dd	10.0	2.3	4.02	dd	10.0	2.3		-0.02	0.0	0.0
3.73	s			3.73	s				0.00		
				2.64	m					n.a.	
2.57	m								0.00		
				2.50	ddt	14.2	13.1	6.5		n.a.	
n.a.				1.94	m				n.a.		
n.a.				1.38	dt	13.9	12.4		n.a.		
1.27	d	7.0		1.28	d	7.1			0.01	0.0	
										-	
1.11	d	7.0		1.10	d	6.9			0.01	0.1	
1.01	d	6.4		1.01	d	6.4			0.00	0.0	

Table S2. Comparison of ^{1}H NMR data of lactone **33** obtained from aldehyde **27** with reported data[10] confirms the desired configuration of the stereocenter at C-8.

Methyl (*4S,5S,6S,8S,E*)-5-(methoxymethoxy)-4,6,8-trimethyl-9-oxonon-2-enoate (8-*epi*-27). A 10-mL Schlenk tube was charged with Rh(acac)(CO)$_2$ (1.0 mg, 3.9 µmol), (S_{ax},*S*,*S*)-BOBPhos (***ent*-32**; 5.0 mg, 7.7 µmol) and hexafluorobenzene (6 mL). The mixture was sonicated until all solids had disappeared. This solution was then transferred into an oven-dried (80 °C), argon-flushed autoclave (equipped with a glass-inlet and stirring bar). The autoclave was sealed, pressurized with hydrogen (7.5 bar) and carbon monoxide (7.5 bar) and the resulting mixture vigorously stirred at 50 °C (external temperature).

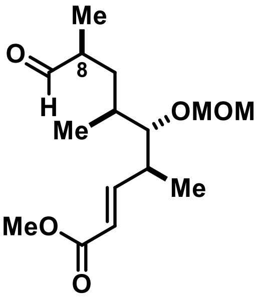

After 1 h, the autoclave was cooled to room temperature, overpressure was released, and a solution of olefin **27** (50.0 mg, 0.195 mmol) in hexafluorobenzene (3 mL) was added to the mixture in an argon counter-flow. The autoclave was again sealed, pressurized with hydrogen/carbon monoxide (7.5 bar each) and warmed to 30 °C (internal temperature after equilibration) while the mixture was vigorously stirred. After 24 h, the overpressure was released, and the contents of the autoclave were transferred to a round-bottom flask. All volatile materials were removed under high vacuum and the residue was subjected to flash chromatography

(hexanes/EtOAc/dichloromethane, 8:1:1) to afford aldehyde **8-*epi*-27** as a colorless liquid (39.9 mg, 71% yield, 8-*epi*-**27**:**27** = 96:4; 8-*epi*-**27**:Σ of all other isomers = 72:28). $[\alpha]_D^{20}$ = –32.0 (*c* 0.99, $CHCl_3$). **^{1}H NMR (400 MHz, $CDCl_3$):** δ 9.62 (d, *J* = 1.7 Hz, 1H), 6.93 (dd, *J* = 15.8, 8.2 Hz, 1H), 5.83 (dd, *J* = 15.8, 1.2 Hz, 1H), 4.63 (d, *J* = 1.2 Hz, 2H), 3.73 (s, 3H), 3.38 (s, 3H), 3.31 – 3.25 (m, 1H), 2.62 (dtd, *J* = 7.9, 6.5, 1.3 Hz, 1H), 2.36 (dddd, *J* = 10.0, 7.1, 4.5, 1.7 Hz, 1H), 1.79 – 1.67 (m, 1H), 1.63 – 1.53 (m, 1H), 1.43 (ddd, *J* = 13.6, 10.0, 3.3 Hz, 1H), 1.10 (d, *J* = 6.7 Hz, 3H), 1.03 (d, *J* = 6.9 Hz, 3H), 0.95 (d, *J* = 6.8 Hz, 3H). **^{13}C{^{1}H} NMR (101 MHz, $CDCl_3$):** δ 205.1, 167.1, 151.8, 120.7, 98.5, 86.8, 56.3, 51.7, 44.3, 39.7, 33.4, 31.7, 16.9, 15.3, 13.0. **IR (film):** 2964, 2937, 2882, 2824, 1723, 1656, 1460, 1437, 1380, 1273, 1178, 1149, 1093, 1033, 920 cm^{-1}. **HRMS-ESI *m/z*:** $[M+Na]^+$ calcd for $C_{15}H_{26}O_5Na$ 309.1672; found 309.1667.

Methyl (4*S*,5*S*,6*S*,8*R*,*E*)-9-hydroxy-5-(methoxymethoxy)-4,6,8-trimethylnon-2-enoate (S8).

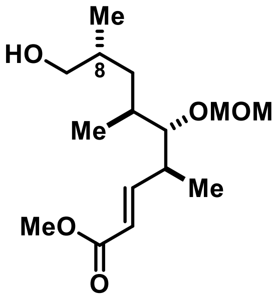

Sodium borohydride (6.3 mg, 0.17 mmol) was added in one portion to a solution of aldehyde **27** (14.5 mg, 50.2 µmol) in methanol (0.5 mL) at −78 °C and the resulting mixture was slowly allowed to warm to room temperature. The mixture was concentrated under reduced pressure, the residue was diluted with *tert*-butyl methyl ether and the organic layer washed with water. The aqueous phase was extracted with *tert*-butyl methyl ether and the combined organic layers were dried over anhydrous magnesium sulfate. The drying agent was filtered off and the solvent was removed under reduced pressure. Purification of the crude material by flash chromatography (hexanes/EtOAc, 1:1) afforded the desired alcohol as a colorless oil (7.6 mg, 52% yield). $[\alpha]_D^{20}$ = –28.1 (*c* 0.62, $CHCl_3$). **^{1}H NMR (400 MHz, $CDCl_3$):** δ 6.96 (dd, *J* = 15.8, 8.2 Hz, 1H), 5.84 (dd, *J* = 15.7, 1.2 Hz, 1H), 4.63 (q, *J* = 6.8 Hz, 2H), 3.73 (s, 3H), 3.48 (dd, *J* = 10.6, 4.9 Hz, 1H), 3.42 – 3.37 (m, 4H), 3.26 (dd, *J* = 6.0, 4.6 Hz, 1H), 2.62 (dddd, *J* = 8.1, 7.1, 6.0, 1.3 Hz, 1H), 1.82 – 1.64 (m, 2H), 1.57 (ddd, *J* = 13.9, 8.6, 4.1 Hz, 1H), 1.09 (d, *J* = 6.7 Hz, 3H), 0.96 (m, *J* = 6.8, 2.2 Hz, 7H). **^{13}C{^{1}H} NMR (101 MHz, $CDCl_3$):** δ 167.2, 152.3, 120.2, 98.1, 86.5, 67.3, 56.1, 51.6, 39.3, 35.7, 33.7, 33.3, 18.3, 17.6, 15.2. **IR (film):** 3434, 2953, 2931, 2881, 2824, 1722, 1654, 1459, 1437, 1273, 1177, 1148, 1094, 1030, 988, 919 cm^{-1}. **HRMS-ESI *m/z*:** $[M+Na]^+$ calcd for $C_{15}H_{28}O_5Na$ 311.1829; found 311.1831.

Methyl (4*S*,5*S*,6*S*,8*S*,*E*)-9-hydroxy-5-(methoxymethoxy)-4,6,8-trimethylnon-2-enoate (S9).

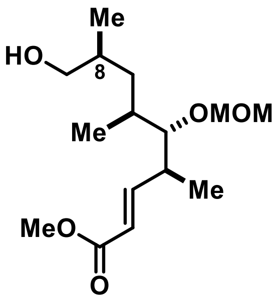

Prepared analogously from **8-*epi*-27** as a colorless oil (23 mg, 99% yield). $[\alpha]_D^{20}$ = –30.9 (*c* 1.0, $CHCl_3$). **^{1}H NMR (400 MHz, $CDCl_3$):** δ 6.95 (dd, *J* = 15.7, 8.2 Hz, 1H), 5.83 (dd, *J* = 15.8, 1.2 Hz, 1H), 4.66 – 4.61 (m, 2H), 3.73 (s, 3H), 3.47 – 3.41 (m, 2H), 3.38 (s, 3H), 3.26 (dd, *J* = 6.1, 4.5 Hz, 1H), 2.68 – 2.58 (m, 1H), 1.79 – 1.63 (m, 2H), 1.26 – 1.21 (m, 2H), 1.10 (d, *J* = 6.8 Hz, 3H), 0.93 (d, *J* = 6.8 Hz, 3H), 0.84 (d, *J* = 6.6 Hz, 3H). **^{13}C{^{1}H} NMR (101 MHz, $CDCl_3$):**

δ 167.2, 152.3, 120.4, 98.4, 87.2, 69.4, 56.2, 51.7, 39.6, 34.7, 33.4, 33.2, 17.1, 15.9, 15.3. **IR (film):** 3461, 2956, 2932, 2879, 2825, 1724, 1655, 1460, 1437, 1273, 1195, 1094, 1034, 989, 919 cm^{-1}. **HRMS-ESI *m/z*:** $[M+Na]^+$ calcd for $C_{15}H_{28}O_5Na^+$ 311.1829, found 311.1827.

Methyl-(*S*,*E*)-4-((2*S*,3*S*,5*R*)-3,5-dimethyl-6-oxotetrahydro-2H-pyran-2-yl)pent-2-enoate

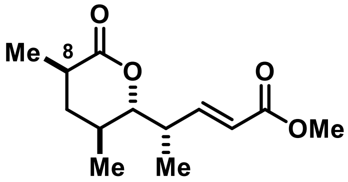

(S10). Concentrated aqueous HCl (0.1 mL) was added to a solution of ether **S8** (7.8 mg, 27 µmol) in acetonitrile (0.4 mL) and the resulting mixture was stirred for 16 h. The reaction was quenched with saturated aqueous sodium bicarbonate solution and the aqueous phase was extracted with dichloromethane. The combined organic layers were dried over anhydrous magnesium sulfate and the drying agent was filtered off. The solvent was removed under reduced pressure to give a colorless oil (6.4 mg).

In a 10-mL round-bottom flask, bis(acetoxy)iodobenzene (19 mg, 0.054 mmol) and TEMPO (0.8 mg, 0.005 mmol) were added to a solution of this material in acetonitrile/water (1:1, 0.25 mL) at 0 °C. After stirring for 24 h at room temperature, the reaction was quenched with saturated aqueous sodium bicarbonate solution. The aqueous phase was extracted with dichloromethane (3 ×1 mL) and the combined organic layers were dried over anhydrous magnesium sulfate. The drying agent was filtered off and the solvent was removed under reduced pressure. Flash chromatography (hexanes/EtOAc, 6:1 → 1:1) afforded the title compound as a colorless oil (1.8 mg, 28% yield). $[\alpha]_D^{20}$ = –98.3 (*c* 0.18, MeOH). **^{1}H NMR (400 MHz, $CDCl_3$):** δ 7.07 (dd, *J* = 15.8, 7.4 Hz, 1H), 5.88 (dd, *J* = 15.8, 1.4 Hz, 1H), 4.02 (dd, *J* = 10.0, 2.3 Hz, 1H), 3.73 (s, 3H), 2.68 – 2.59 (m, 1H), 2.56 – 2.44 (m, 1H), 1.98 – 1.89 (m, 2H), 1.38 (dt, *J* = 13.9, 12.4 Hz, 1H), 1.28 (d, *J* = 7.0 Hz, 3H), 1.10 (d, *J* = 6.9 Hz, 3H), 1.01 (d, *J* = 6.4 Hz, 3H). **^{13}C{^{1}H} NMR (101 MHz, $CDCl_3$):** δ 174.0, 166.8, 150.4, 121.4, 88.4, 51.5, 38.2, 37.5, 36.2, 31.0, 17.3, 11.7. **IR (film):** 2969, 2936, 2881, 1724, 1658, 1459, 1436, 1380, 1322, 1273, 1256, 1192, 1165, 1108, 1039, 994 cm^{-1}. **HRMS-ESI *m/z*:** $[M+Na]^+$ calcd for $C_{13}H_{20}O_4Na^+$ 263.1254, found 263.1259.

Methyl (*S*,*E*)-4-((2*S*,3*S*,5*S*)-3,5-dimethyl-6-oxotetrahydro-2H-pyran-2-yl)pent-2-enoate

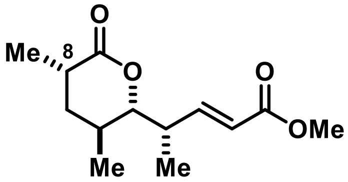

(S11). Prepared analogously from **S0** as a colorless oil (2 mg, 47% yield). $[\alpha]_D^{20}$ = +75.0 (*c* 0.13, MeOH). **^{1}H NMR (400 MHz, $CDCl_3$):** δ 7.02 (dd, *J* = 15.8, 7.6 Hz, 1H), 5.88 (dd, *J* = 15.8, 1.3 Hz, 1H), 3.98 (dd, *J* = 9.7, 2.9 Hz, 1H), 3.72 (s, 3H), 2.67 – 2.58 (m, 2H), 2.00 (dq, *J* = 9.7, 7.1 Hz, 1H), 1.70 (ddd, *J* = 8.2, 7.5, 5.7 Hz, 2H), 1.21 (d, *J* = 6.9 Hz, 3H), 1.13 (d, *J* = 6.9 Hz, 3H), 1.02 (d, *J* = 6.8 Hz, 3H).**^{13}C{^{1}H} NMR (101 MHz, $CDCl_3$):** δ 175.7, 166.8, 150.2, 121.4, 84.8, 51.5, 37.9, 34.9, 32.3, 28.6, 17.7, 16.4, 12.0. **IR (film):** 3287, 2952, 2840, 1647, 1450, 1407, 1197, 1113, 1014, 533, 447 cm^{-1}. **HRMS-ESI *m/z*:** $[M+Na]^+$ calcd for $C_{13}H_{20}O_4Na^+$ 263.1254, found 263.1254.

Fragment Coupling and Completion of the Total Synthesis of Mycinolide IV

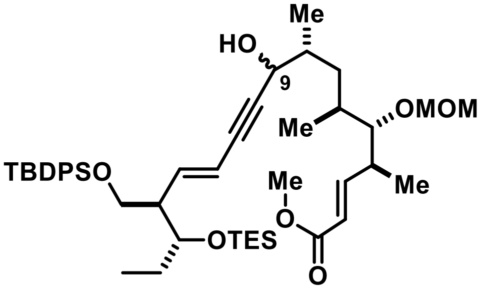

Propargyl Alcohol 28. *n*-BuLi (1.6 M in hexanes, 1.8 mL, 2.9 mmol) was added to a solution of alkyne **16** (1.44 g, 2.84 mmol) in THF (40 mL) at –78 °C, giving a pale yellow solution. After 1 h, a solution of aldehyde **27** (675 mg, 2.36 mmol) in THF (7 mL) was added, and the resulting mixture was stirred at –78 °C for 1.5 h. Pentane (40 mL) was added, the reaction was quenched with water (20 mL) and the mixture allowed to warm to room temperature. Saturated aqueous NaCl (10 mL) was introduced, the layers were separated and the aqueous phase was extracted with pentane (3 × 10 mL). The combined organic layers were washed with saturated aqueous NaCl, dried over anhydrous sodium sulfate and the drying agent was removed by filtration. The filtrate was evaporated to dryness, giving a pale yellow oil. Purification by automated column chromatography (for each half of the crude product using a Biotage® 50 g SNAP Ultra HP-Sphere™ 25μm cartridge, loading as a solution in hexanes, gradient of 4 − 50% EtOAc in hexanes over 15 column volumes) afforded the title compound as a colorless oil (1.04 g, 56% yield, 1.4:1.0 ratio of C-9 epimers). *Analytical and spectral data of the mixture of C-9 epimers (NMR integrals of overlapping signals given with respect to single epimer)*: **^{1}H NMR (400 MHz, $CDCl_3$):** δ 7.69 – 7.59 (m, 4H), 7.46 – 7.32 (m, 6H), 7.02 – 6.89 (m, 1H), 6.10 – 6.00 (m, 1H), 5.89 – 5.80 (m, 1H), 5.46 – 5.38 (m, 1H), 4.68 – 4.58 (m, 2H), 4.43 – 4.39 (m, 1H, *single epimer*), 4.36 – 4.31 (m, 1H, *single epimer*), 3.85 – 3.79 (m, 1H), 3.78 – 3.72 (m, 1H), 3.72 (s, 3H, *single epimer*), 3.70 (s, 3H, *single epimer*), 3.55 – 3.48 (m, 1H), 3.38 (s, 3H, *single epimer*), 3.37 (s, 3H, *single epimer*), 3.29 – 3.23 (m, 1H), 2.70 – 2.55 (m, 1H), 2.32 – 2.23 (m, 1H), 1.90 – 1.75 (m, 3H), 1.74 – 1.64 (m, 1H), 1.47 – 1.36 (m, 2H), 1.09 (d, *J* = 6.7 Hz, 3H, *single epimer*), 1.08 (d, *J* = 6.7 Hz, 3H, *single epimer*), 1.05 (s, 9H), 1.02 (d, *J* = 6.7 Hz, 3H, *single epimer*), 1.01 (d, *J* = 6.4 Hz, 3H, *single epimer*), 0.97 (d, *J* = 6.8 Hz, 3H, *single epimer*), 0.96 (d, *J* = 6.7 Hz, *single epimer*), 0.91 (t, *J* = 7.9 Hz, 9H), 0.772 (t, *J* = 7.5 Hz, 3H, *single epimer*), 0.769 (t, *J* = 7.4 Hz, 1H, *single epimer*), 0.61 – 0.50 (m, 6H). **$^{13}C\{^1H\}$ NMR (101 MHz, $CDCl_3$):** δ 167.2, 167.1, 152.32, 152.25, 142.2, 142.0, 135.7, 135.6, 133.8, 133.7, 129.63, 129.56, 127.6, 120.34, 120.29, 111.9, 111.8, 98.01, 98.00, 88.2, 87.2, 86.4, 86.2, 84.8, 84.2, 72.5, 66.8, 65.7, 64.4, 56.04, 56.01, 51.5, 51.4, 50.2, 39.3, 39.2, 37.12, 37.10, 35.6, 34.5, 33.8, 33.7, 28.3, 26.9, 19.2, 17.83, 17.79, 16.1, 16.0, 15.3, 15.1, 9.9, 6.9, 5.1. **IR (film):** 2957, 2933, 2875, 1724, 1655, 1461, 1428, 1378, 1337, 1243, 1148, 1084, 1031, 962, 920, 822, 737, 701, 612, 503, 488 cm^{-1}. **HRMS-ESI *m/z*:** $[M+Na]^+$ calcd for $C_{46}H_{72}O_7Si_2Na$ 815.4709; found 815.4700.

Macrocyclization Precursor 29. Camphor-10-sulfonic acid (12 mg, 52 µmol) was added to a pre-cooled solution of alcohol **28** (818 mg, 1.03 mmol) in MeOH/CH_2Cl_2 (23 mL each) at –20 °C (internal temperature). After 2 h, *tert*-butyl methyl ether (40 mL) and half-concentrated aqueous sodium bicarbonate solution (20 mL) were added and the mixture was warmed to room temperature. The layers were separated and the aqueous phase was extracted with *tert*-butyl methyl ether (3 × 10 mL). The combined organic layers were washed with brine (10 mL), dried over anhydrous sodium sulfate, and the drying agent was removed by filtration. The filtrate was evaporated to dryness, affording a pale yellow oil. Purification of the crude product by automated column chromatography (Biotage® 50 g SNAP Ultra HP-Sphere™ 25µm cartridge, loading as a solution in CH_2Cl_2/hexanes = 1:1, gradient of 4 – 50% EtOAc in hexanes over 20 column volumes) yielded the title compound as a colorless oil (601 mg, 86% yield, dr = 1.4:1). *Analytical data for mixture of C-9 epimers (NMR integrals of overlapping signals given with respect to single epimer)*: **^{1}H NMR (400 MHz, $CDCl_3$):** δ 7.69 – 7.62 (m, 4H), 7.49–7.34 (m, 6H), 7.02 – 6.89 (m, 1H), 6.27 – 6.15 (m, 1H), 5.89 – 5.80 (m, 1H), 5.58 – 5.48 (m, 1H), 4.68 – 4.58 (m, 2H), 4.44 – 4.39 (m, 1H, *single epimer*), 4.35 (dd, *J* = 5.1, 1.8 Hz, 1H, *single epimer*), 3.85 – 3.74 (m, 3H), 3.72 (s, 3H, *single epimer*), 3.70 (s, 3H, *single epimer*), 3.379 (s, 3H, *single epimer*), 3.376 (s, 3H, *single epimer*), 3.30 – 3.23 (m, 1H), 2.69 – 2.56 (m, 1H), 2.37 – 2.26 (m, 1H), 1.91 – 1.73 (m, 3H), 1.72 – 1.62 (m, 1H), 1.55 – 1.34 (m, 2H), 1.09 (d, *J* = 6.7 Hz, 3H, *single epimer*), 1.08 (d, *J* = 6.8 Hz, 3H, *single epimer*), 1.06 (s, 9H), 1.021 (d, *J* = 6.7 Hz, 3H, *single epimer*), 1.016 (d, *J* = 6.4 Hz, 3H, *single epimer*), 0.98 (d, *J* = 6.9 Hz, 3H, *single epimer*), 0.97 (d, *J* = 6.7 Hz, 3H, *single epimer*), 0.922 (t, *J* = 7.4 Hz, 3H, *single epimer*), 0.920 (t, *J* = 7.4 Hz, 3H, *single epimer*). **$^{13}C\{^1H\}$ NMR (101 MHz, $CDCl_3$)** δ 167.24, 167.15, 152.3, 152.2, 141.2, 140.9, 135.6, 135.5, 133.01, 132.97, 132.85, 132.80, 129.9, 127.8, 120.4, 120.3, 112.44, 112.38, 98.02, 97.98, 88.8, 87.7, 86.3, 86.2, 84.4, 83.8, 73.9, 73.8, 66.7, 66.3, 66.1, 65.7, 56.1, 56.0, 51.53, 51.50, 49.7, 49.6, 39.3, 39.2, 37.1, 37.0, 35.4, 34.5, 33.8, 33.6, 27.8, 26.9, 19.2, 17.8, 17.7, 16.2, 16.0, 15.5, 15.1, 10.3. **IR (film):** 3454, 2959, 2931, 1721, 1654, 1461, 1428, 1380, 1278, 1147, 1106, 1030, 961, 914, 864, 823, 801, 736, 702, 613, 504, 433 cm^{-1}. **HRMS-ESI *m/z*:** $[M+Na]^+$ calcd for $C_{40}H_{58}O_7SiNa$ 701.3844; found 701.3840.

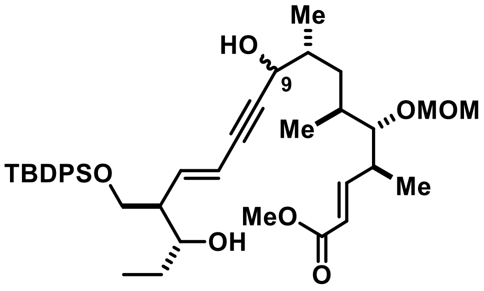

Macrolactone 30. In a 1-L two-necked flask equipped with a reflux-condenser and an argon bridge, compound **29** (600 mg, 0.884 mmol) and distannoxane **25b** (1.96 g, 1.77 mmol) were dissolved in chlorobenzene (600 mL) and the resulting solution was stirred at reflux temperature for 89 h. The solvent was then removed under reduced pressure to afford a yellow solid. Purification by flash chromatography on silica gel (15 − 40 µm particle size; *tert*-butyl methyl ether/hexanes, 1:4 to 1:3) afforded macrolide **30** as a colourless foam (184 mg, 32% yield, dr = 2.2:1).

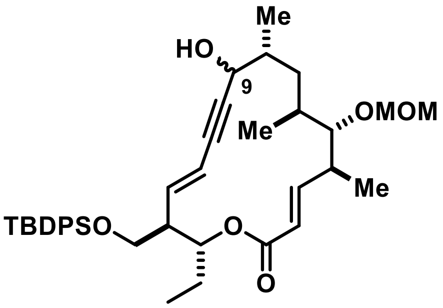

For analytical purposes, an aliquot was re-subjected to flash chromatography to give a pure sample of one of the C-9 epimers. The signals of the second isomer had to be assigned from the NMR spectrum of the mixture containing both C-9 epimers.

Spectral data of epimer 1: **^{1}H NMR (400 MHz, $CDCl_3$):** δ 7.68 – 7.59 (m, 4H), 7.49 – 7.34 (m, 6H), 6.77 (dd, *J* = 15.5, 9.7 Hz, 1H), 6.07 (dd, *J* = 15.8, 10.0 Hz, 1H), 5.81 (dd, *J* = 15.5, 0.7 Hz, 1H), 5.46 (dd, *J* = 15.8, 2.0 Hz, 1H), 5.02 (td, *J* = 9.6, 2.6 Hz, 1H), 4.72 – 4.65 (m, 2H), 4.58 (dd, *J* = 4.1, 2.3 Hz, 1H), 3.74 – 3.62 (m, 2H), 3.42 (s, 3H), 3.21 (dd, *J* = 9.9, 2.2 Hz, 1H), 2.66 – 2.50 (m, 1H), 2.29 (tt, *J* = 9.8, 4.5 Hz, 1H), 2.00 – 1.88 (m, 1H), 1.76 – 1.57 (m, 2H), 1.45 – 1.35 (m, 1H), 1.34 – 1.28 (m, 2H), 1.12 (d, *J* = 6.8 Hz, 3H), 1.07 (s, 9H), 1.01 (d, *J* = 6.9 Hz, 3H), 1.00 (d, *J* = 6.5 Hz, 3H), 0.90 – 0.84 (m, 3H). **$^{13}C\{^1H\}$ NMR (101 MHz, $CDCl_3$):** δ 166.7, 150.6, 143.6, 135.64, 135.59, 133.2, 133.1, 129.8, 129.7, 127.7, 121.7, 112.5, 99.4, 88.6, 86.3, 84.2, 73.9, 64.5, 63.2, 56.3, 52.1, 40.3, 35.1, 32.9, 31.1, 26.8, 25.3, 19.8, 19.2, 16.9, 15.2, 9.8.

Spectral data of epimer 2: **^{1}H NMR (400 MHz, $CDCl_3$):** δ 7.68 – 7.60 (m, 4H), 7.47 – 7.35 (m, 6H), 6.87 (dd, *J* = 15.6, 9.2 Hz, 1H), 6.02 (dd, *J* = 15.8, 9.8 Hz, 1H), 5.81 (d, *J* = 15.5 Hz, 1H), 5.46 (dd, *J* = 15.8, 2.1 Hz, 1H), 5.07 – 4.96 (m, 1H), 4.72 – 4.65 (m, 2H), 4.14 (dd, *J* = 7.0, 1.8 Hz, 1H), 3.75 – 3.62 (m, 2H), 3.41 (s, 3H), 3.25 (dd, *J* = 8.5, 3.0 Hz, 1H), 2.70 – 2.53 (m, 1H), 2.38 – 2.23 (m, 1H), 2.01 – 1.86 (m, 1H), 1.75 – 1.58 (m, 2H), 1.46 – 1.34 (m, 1H), 1.34 – 1.26 (m, 2H), 1.13 (d, *J* = 6.8 Hz, 3H), 1.07 (s, 9H), 1.04 – 0.98 (m, 6H), 0.89 – 0.83 (m, 3H). **$^{13}C\{^1H\}$ NMR (101 MHz, $CDCl_3$):** δ 166.7, 151.4, 142.2, 135.64, 135.59, 133.20, 133.17, 133.13, 129.8, 129.7, 127.7, 121.2, 112.1, 97.9, 88.4, 86.3, 84.5, 74.1, 68.8, 63.5, 56.1, 51.7, 39.5, 35.0, 32.9, 31.1, 26.8, 25.5, 19.8, 19.0, 16.9, 15.2, 9.7. **IR (film):** 3468, 3071, 2959, 2929, 2873, 2857, 1717, 1651, 1461, 1428, 1240, 1222, 1176, 1147, 1104, 1030, 988, 858, 823, 792, 740, 702, 607, 504, 432 cm^{-1}. **HRMS-ESI *m/z*:** $[M+Na]^+$ calcd for $C_{39}H_{54}O_6SiNa$ 669.3582; found: 669.3583.

Dienone 31. A solution of $PhPCy_2$ (52 mg, 0.19 mmol) in degassed THF (7 mL) was added to a stirred suspension of $[CpRu(MeCN)_3]BF_4$ (71 mg, 0.19 mmol) in degassed THF (20 mL) at room temperature. The resulting slightly turbid solution was stirred for 5 min before it was added to a solution of macrolide **30** (249 mg, 0.385 mmol) in degassed THF (23 mL). The resulting mixture was stirred at reflux temperature for 2.5 h. After cooling, the mixture was diluted with hexanes (50 mL) and filtered through a silica pad, which was rinsed with EtOAc/hexanes (1:1, 200 mL). The combined filtrates were evaporated to dryness, giving a yellow oil. Purification by automated column chromatography (Biotage® 50 g SNAP Ultra HP-Sphere™ 25μm cartridge, loading as a solution in hexanes, gradient of 2 – 50% EtOAc in hexanes over 20 column volumes) yielded dienone **31** as an off-white foam (162 mg, 65% yield). **mp** = 58 – 62 °C. $[\alpha]_D^{25}$ = +45.2 (*c* 0.80, $CHCl_3$). **^{1}H NMR (400 MHz, $CDCl_3$):** δ 7.68 – 7.60 (m, 4H), 7.47 – 7.34 (m, 6H), 7.19 – 7.09 (m, 1H), 6.60 (dd, *J* = 15.5, 9.9 Hz, 1H), 6.22 (d, *J* = 15.1 Hz, 1H), 6.17 – 6.03 (m, 2H), 5.76 (dd, *J* = 15.5, 0.7 Hz, 1H), 4.70 – 4.62 (m, 2H), 4.64 (d, *J* = 6.5 Hz, 1H), 3.79 – 3.69 (m, 2H), 3.41 (s, 3H), 3.15 (dd, *J* = 10.3, 1.4 Hz, 1H), 2.65 – 2.52 (m, 2H), 2.39 – 2.29 (m, 1H), 1.72 – 1.58 (m, 1H), 1.54 – 1.47 (m, 2H), 1.47 – 1.36 (m, 1H), 1.35 – 1.27 (m, 1H), 1.18 (d, *J* = 6.9 Hz, 3H), 1.10 (d, *J* = 6.8 Hz, 3H), 1.06 (s, 9H), 0.99 (d, *J* = 6.8 Hz, 3H), 0.91 – 0.83 (m, 3H). **^{13}C{^{1}H} NMR (101 MHz, $CDCl_3$):** δ 203.4, 166.1, 151.1, 142.3, 141.7, 135.62, 135.58, 133.2, 133.1, 132.8, 129.83, 129.80, 127.7, 122.9, 121.3, 99.3, 88.7, 73.8, 62.7, 56.3, 51.1, 44.7, 40.4, 34.1, 32.5, 26.8, 25.0, 19.5, 19.3, 17.7, 17.6, 9.7. **IR (film):** 2962, 2931, 2880, 2858, 1714, 1681, 1652, 1594, 1461, 1428, 1352, 1327, 1233, 1177, 1147, 1106, 1037, 983, 919, 823, 791, 735, 702, 648, 609, 504 cm^{-1}. **HRMS-ESI *m/z*:** $[M+Na]^+$ calcd for $C_{39}H_{54}O_6SiNa$ 669.3582; found: 669.3590.

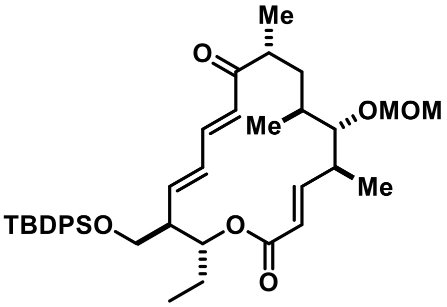

Mycinolide IV (2). Aqueous HCl (3.0 M, 0.71 mL, 2.1 mmol) was added to a solution of dienone **31** (14.3 mg, 22.1 μmol) in methanol (2.3 mL) and the resulting cloudy solution was stirred at 40 °C for 4.5 h. After reaching ambient temperature, the by then clear colourless solution was diluted with water (10 mL) and ethyl acetate (10 mL), and the resulting mixture was vigorously stirred for 2 min. The layers were separated and the aqueous phase was extracted with ethyl acetate (4 × 4 mL). The combined organic layers were washed with saturated aqueous sodium chloride solution (5 mL) and filtered through a pad of anhydrous sodium sulfate. The solvent was removed under reduced pressure to give a colourless solid, which was purified by automated column chromatography (Biotage® 10 g SNAP Ultra HP-Sphere™ 25μm

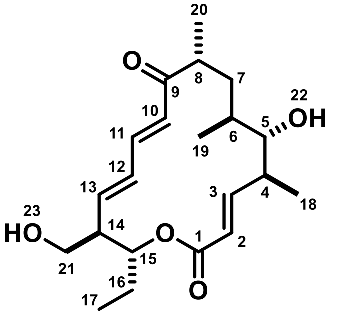

cartridge, loading immobilized on silica, gradient of 16 – 100% EtOAc in hexanes over 15 column volumes) to afford mycinolide IV (**2**) as a colourless solid (6.0 mg, 74% yield). Trace impurities were removed by prepartive HPLC for analytical purposes: Agilent 1260 Infinity pump, 150 mm length × 10 mm diameter YMC Triart C18 5 μm column, methanol/water (60:40 v/v, 4.7 mL/min, 12.6 MPa, 299 K) eluent, UV-detection at 220 nm. **mp** 220 – 222 °C (lit.:[11] 222 – 223 °C). $[\boldsymbol{\alpha}]_{\mathbf{D}}^{\mathbf{25}}$ = +26.5 (*c* 0.28, MeOH) (lit.:[12] $[\boldsymbol{\alpha}]_{\mathbf{D}}^{\mathbf{27}}$ = +24.3 (*c* 0.50, MeOH)). **^{1}H NMR (600 MHz, $CDCl_3$):** δ 7.12 (dd, *J* = 15.0, 11.0 Hz, 1H, 11-H), 6.61 (dd, *J* = 15.5, 9.9 Hz, 1H, 3-H), 6.25 (d, *J* = 15.0 Hz, 1H, 10-H), 6.18 (dd, *J* = 15.2, 11.0 Hz, 1H, 12-H), 5.93 (dd, *J* = 15.2, 9.4 Hz, 1H, 13-H), 5.78 (d, *J* = 15.5 Hz, 1H, 2-H), 4.87 (ddd, *J* = 9.7, 9.0, 2.9 Hz, 1H, 15-H), 3.80 (dd, *J* = 10.8, 4.0 Hz, 1H, 21-H^A), 3.74 (dd, *J* = 10.8, 7.0 Hz, 1H, 21-H^B), 3.29 (dd, *J* = 10.3, 1.5 Hz, 1H, 5-H), 2.63 – 2.39 (m, 3H, 4-H, 8-H, 14-H), 1.83 (dqd, *J* = 14.7, 7.4, 2.9 Hz, 1H, 16-H^A), 1.58 (dquint, *J* = 14.7, 9.0, 7.4 Hz, 1H, 16-H^B), 1.52 – 1.42 (m, 2H, 7-$H^{A,B}$), 1.28 (dquintd, *J* = 11.0, 6.8, 6.0, 1.5 Hz, 1H, 6-H), 1.18 (d, *J* = 7.0 Hz, 3H, 20-H_3), 1.12 (d, *J* = 6.7 Hz, 3H, 18-H_3), 1.00 (d, *J* = 6.8 Hz, 3H, 19-H_3), 0.96 (t, *J* = 7.4 Hz, 3H, 17-H_3). **$^{13}C\{^1H\}$ NMR (151 MHz, $CDCl_3$):** δ 203.2 (C-9), 166.1 (C-1), 151.3 (C-3), 141.7 (C-11), 140.6 (C-13), 134.1 (C-12), 123.4 (C-10), 121.1 (C-2), 80.2 (C-5), 73.5 (C-15), 62.2 (C-21), 51.6 (C-14), 44.7 (C-8), 40.4 (C-4), 33.8 (C-6), 31.6 (C-7), 25.4 (C-16), 19.3 (C-18), 17.7 (C-20), 17.3 (C-19), 9.7 (C-17). **^{1}H NMR (600 MHz, [D_6]-acetone):** δ 7.01 (ddd, *J* = 15.0, 11.1, 0.7 Hz, 1H, 11-H), 6.55 (dd, *J* = 15.5, 10.1 Hz, 1H, 3-H), 6.48 (dt, *J* = 15.0, 0.7 Hz, 1H, 10-H), 6.24 (ddt, *J* = 15.3, 11.1, 0.7 Hz, 1H, 12-H), 6.03 (ddt, *J* = 15.3, 9.4, 0.8 Hz, 1H, 13-H), 5.91 (dd, *J* = 15.5, 0.7 Hz, 1H, 2-H), 4.93 (td, *J* = 10.2, 9.0, 2.8 Hz, 1H, 15-H), 3.90 (t, *J* = 5.3 Hz, 1H, 23-H), 3.81 (d, *J* = 6.0 Hz, 1H, 22-H), 3.76 (dt, *J* = 10.9, 5.3 Hz, 1H, 21-H^A), 3.72 (ddd, *J* = 10.9, 5.3, 3.6 Hz, 1H, 21-H^B), 3.26 (ddd, *J* = 10.4, 6.0, 1.5 Hz, 1H, 5-H), 2.57 (tqd, *J* = 10.1, 6.7, 0.7 Hz, 1H, 4-H), 2.45 (dqd, *J* = 12.2, 6.9, 4.4 Hz, 1H, 8-H), 2.38 (ddddd, *J* = 10.0, 9.4, 5.3, 3.6, 0.8 Hz, 1H, 14-H), 1.90 (dqd, *J* = 14.5, 7.4, 2.8 Hz, 1H, 16-H^A), 1.65 (ddd, *J* = 13.9, 12.2, 3.4 Hz, 1H, 7-H^A), 1.57 (ddq, *J* = 14.5, 9.0, 7.4 Hz, 1H, 16-H^B), 1.49 (ddd, *J* = 13.9, 12.4, 4.4 Hz, 1H, 7-H^B), 1.18 (dqdd, *J* = 12.4, 6.8, 3.4, 1.5 Hz, 1H, 6-H), 1.15 (d, *J* = 6.9 Hz, 3H, 20-H_3), 1.08 (d, *J* = 6.7 Hz, 3H, 18-H_3), 0.95 (d, *J* = 6.8 Hz, 3H, 19-H_3), 0.91 (t, *J* = 7.3 Hz, 3H, 17-H_3). **$^{13}C\{^1H\}$ NMR (151 MHz, [D_6]-acetone):** δ 201.1 (C-9), 164.7 (C-1), 150.6 (C-3), 140.3 (C-13), 140.1 (C-11), 132.1 (C-12), 122.7 (C-10), 120.3 (C-2), 78.1 (C-5), 72.5 (C-15), 59.9 (C-21), 50.2 (C-14), 43.9 (C-8), 39.5 (C-4), 33.1 (C-6), 30.3 (C-7), 23.8 (C-16), 17.9 (C-18), 15.9 (C-20), 15.9 (C-19), 8.2 (C-17). **IR (film):** 3419, 2964, 292, 1704, 1670, 1650, 1588, 1348, 1279, 1228, 1174, 1152, 1057, 1005, 988, 847. **HRMS-ESI *m/z*:** $[M+Na]^+$ calcd for $C_{21}H_{32}O_5Na$ 387.2142; found: 387.2143.

*Comparison of the NMR spectra of mycinolide IV (**2**) prepared by total synthesis with those of the authentic sample obtained by hydrolysis of mycinamicin IV* [11,12]

Figure S1 shows a visual comparison of the recorded ^{13}C NMR spectrum ($CDCl_3$) of synthetic **2** (*in black*) and a simulated spectrum created in MestReNova based on the ^{13}C NMR shifts of authentic mycinolide IV in $CDCl_3$ reported in the literature (*in red*). The comparison of the numeric ^{13}C NMR shifts confirms the excellent agreement ($\Delta\delta \leq 0.1$ ppm).

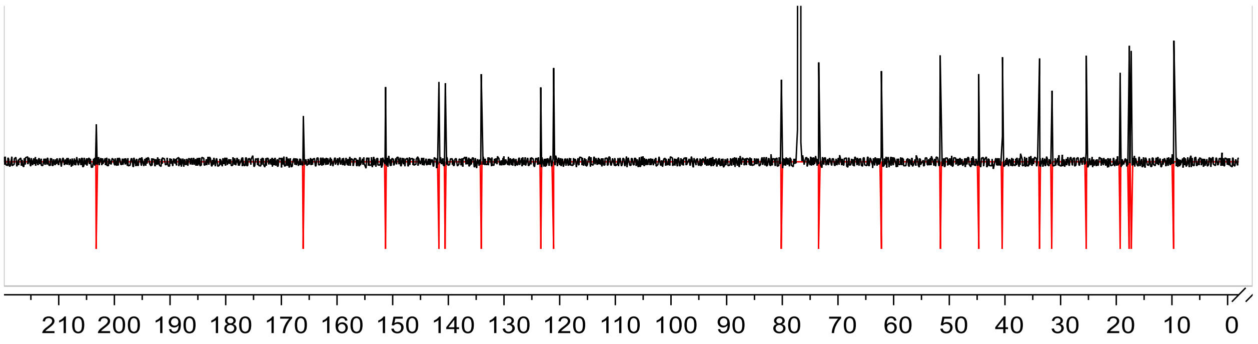

Figure S1. ^{13}C NMR shifts ($CDCl_3$, ppm) of synthetic **2** (*in black*) and authentic mycinolide IV (*in red*).[12]

Figure S2 shows a visual comparison of a "virtual" 100 MHz ^{1}H NMR spectrum of synthetic mycinolide IV (2) (simulated on the basis of the experimental NMR data recorded at 600 MHz in [D_6]-acetone) (*in red*) with the ^{1}H NMR spectrum (in [D_6]-acetone/D_2O) reported in the literature of authentic mycinolide IV obtained from isolated mycinamicin IV by acid-mediated glycoside hydrolysis (*in black*).[12] Since the latter spectrum was only graphically depicted, and no tabulation of chemical shifts and coupling constants was reported, the comparison is limited to this purely graphical and qualitative assessment.

The frequency of the spectrum reported in the literature was not specified; it is assumed to be 100 MHz based on spectra of several mycinamicins reported by the same groups two years later.[13] Since no NMR spectrometer was available to us to record a ^{1}H NMR spectrum of the synthesized material at 100 MHz, the spectrum was recorded at 600 MHz and all proton chemical shifts were assigned and coupling constants were extracted. This information was then used for a simulation of a 100 MHz ^{1}H NMR spectrum, using the DAISY module in TOPSPIN. Additionally, –OH signals of the recorded spectrum and their couplings to neighbouring protons were removed to account for their invisibility in the literature spectrum due to the presence of an undefined amount of D_2O.

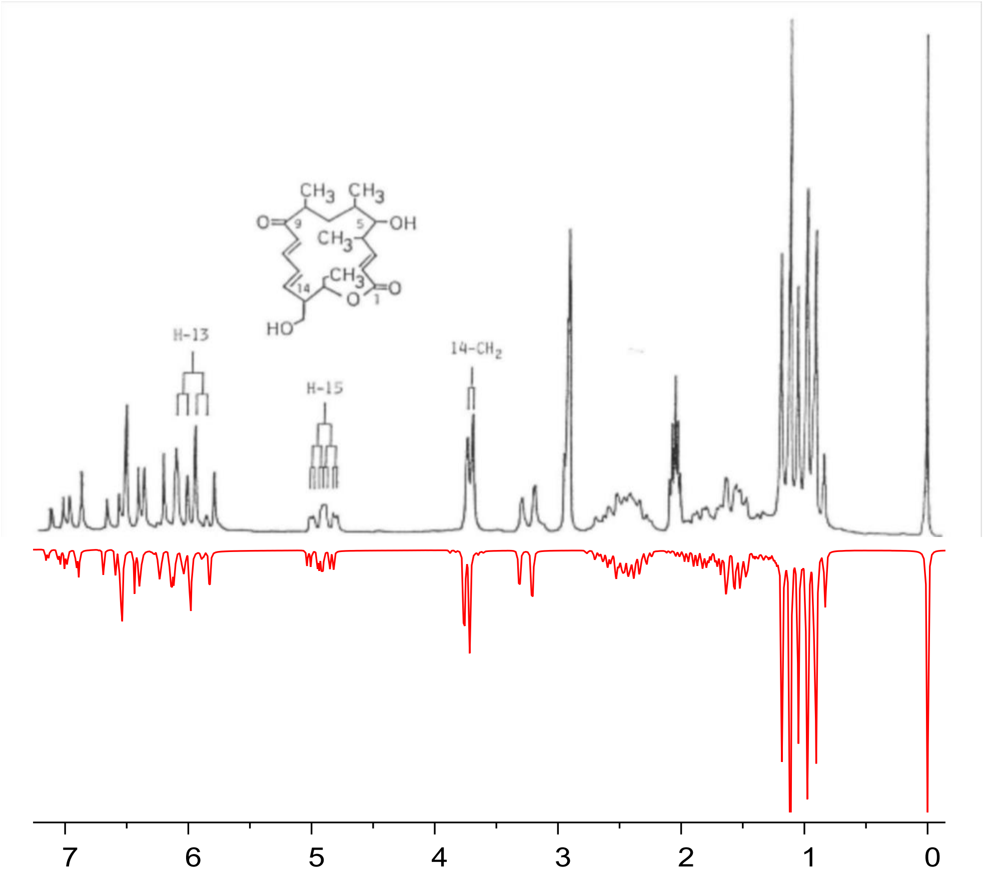

Figure S2. ^{1}H NMR shifts (ppm) of synthetic **2** (*in red*) and authentic mycinolide IV obtained from the hydrolysis of mycinamicin IV as reported in the literature (*in black*).[12]

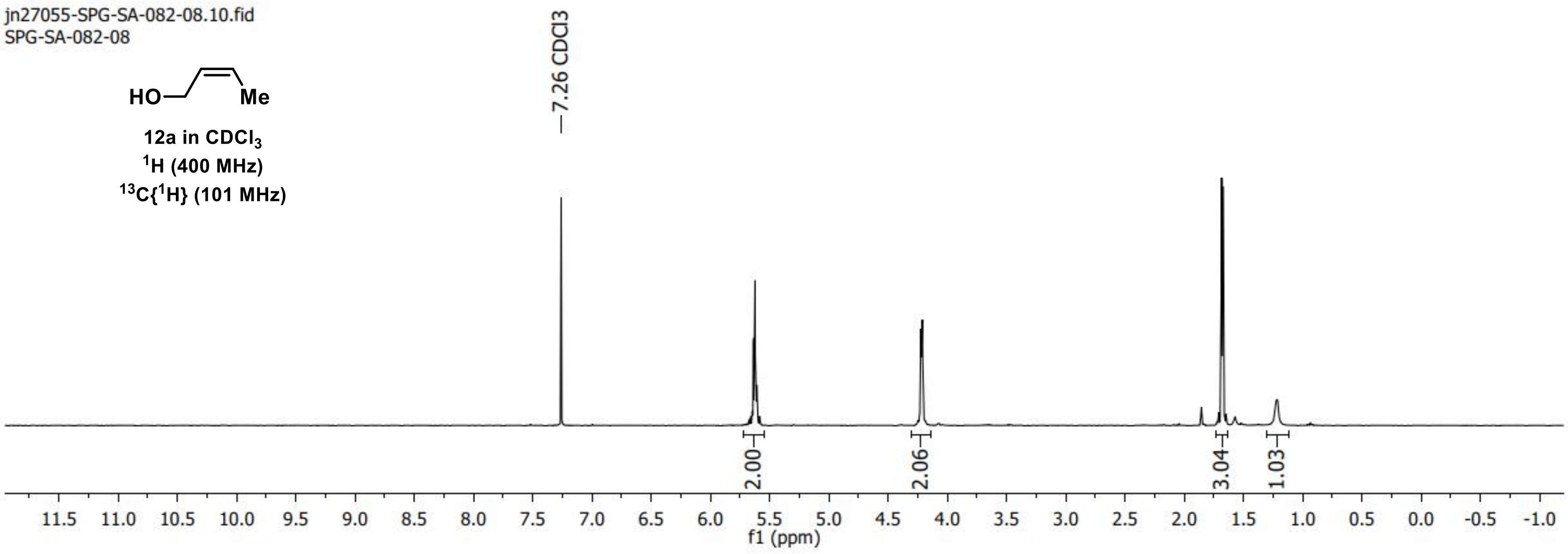
jn27055-SPG-SA-082-08.10.fid
SPG-SA-082-08
HO
Me
12a in CDCl3
1H (400 MHz)
13C{1H} (101 MHz)
7.26 CDCl3
2.00
2.06
3.04
1.03
11.5 11.0 10.5 10.0 9.5 9.0 8.5 8.0 7.5 7.0 6.5 6.0 5.5 5.0 4.5 4.0 3.5 3.0 2.5 2.0 1.5 1.0 0.5 0.0 -0.5 -1.0
f1 (ppm)

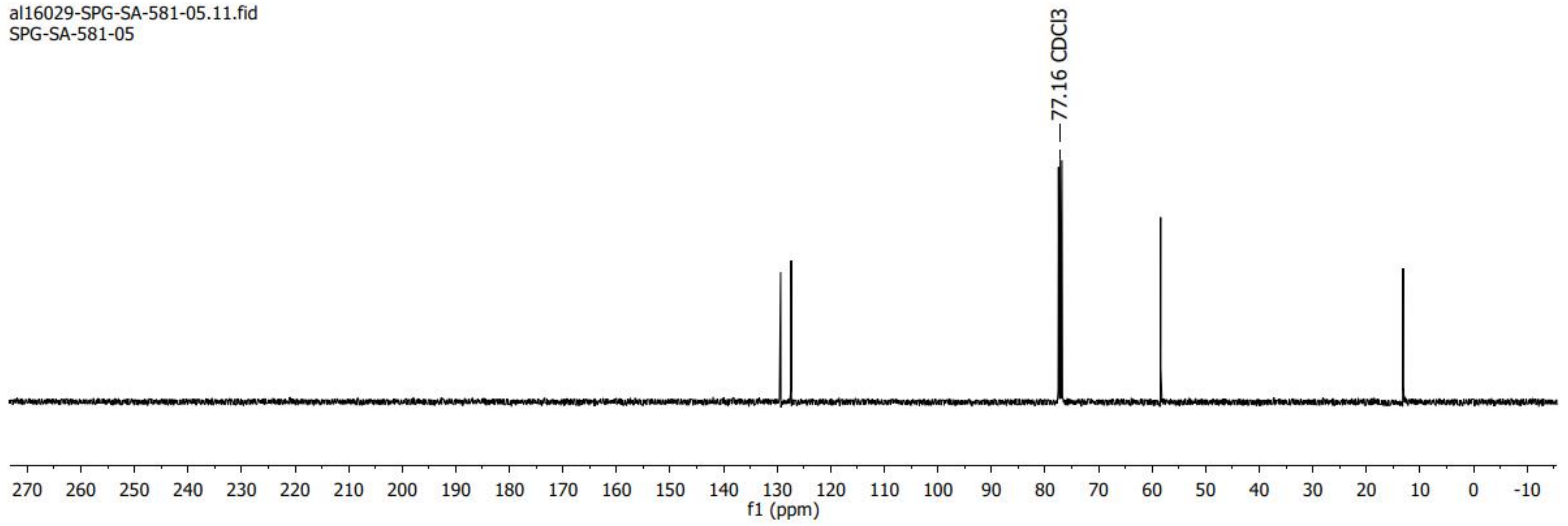
al16029-SPG-SA-581-05.11.fid
SPG-SA-581-05
77.16 CDCl3
270 260 250 240 230 220 210 200 190 180 170 160 150 140 130 120 110 100 90 80 70 60 50 40 30 20 10 0 -10
f1 (ppm)

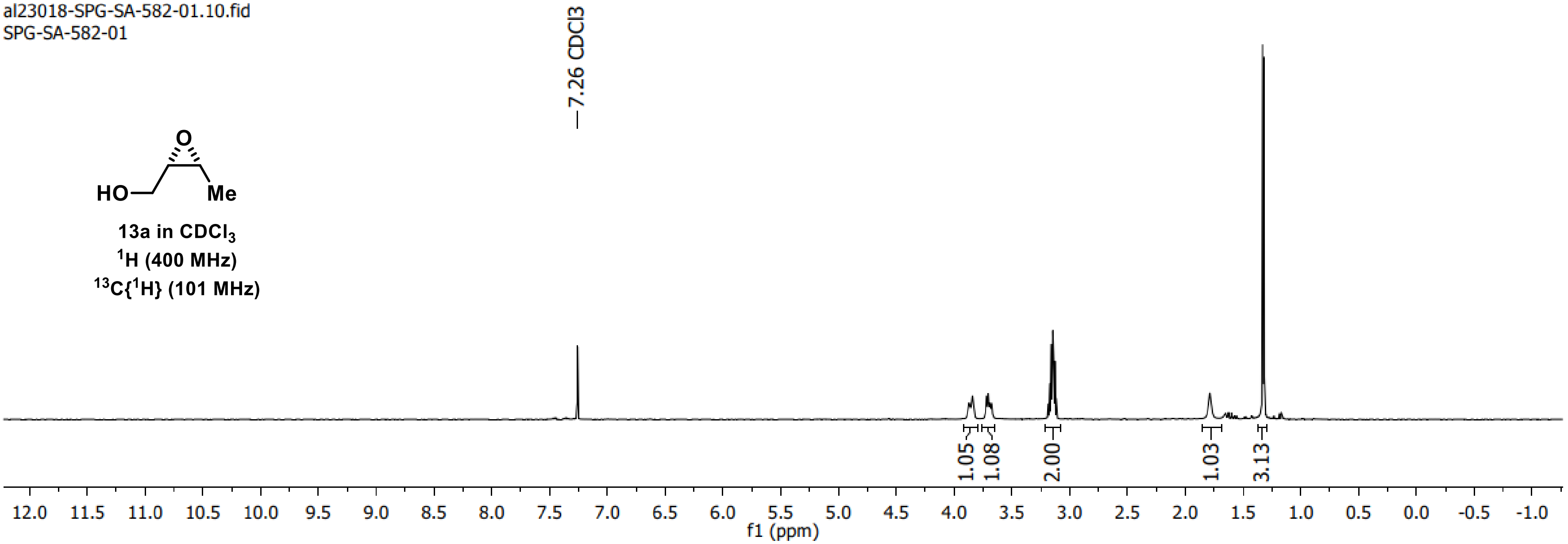

al23018-SPG-SA-582-01.10.fid
SPG-SA-582-01
HO
O
Me
13a in CDCl3
1H (400 MHz)
13C{1H} (101 MHz)
7.26 CDCl3
1.05
1.08
2.00
1.03
3.13
12.0 11.5 11.0 10.5 10.0 9.5 9.0 8.5 8.0 7.5 7.0 6.5 6.0 5.5 5.0 4.5 4.0 3.5 3.0 2.5 2.0 1.5 1.0 0.5 0.0 -0.5 -1.0
f1 (ppm)

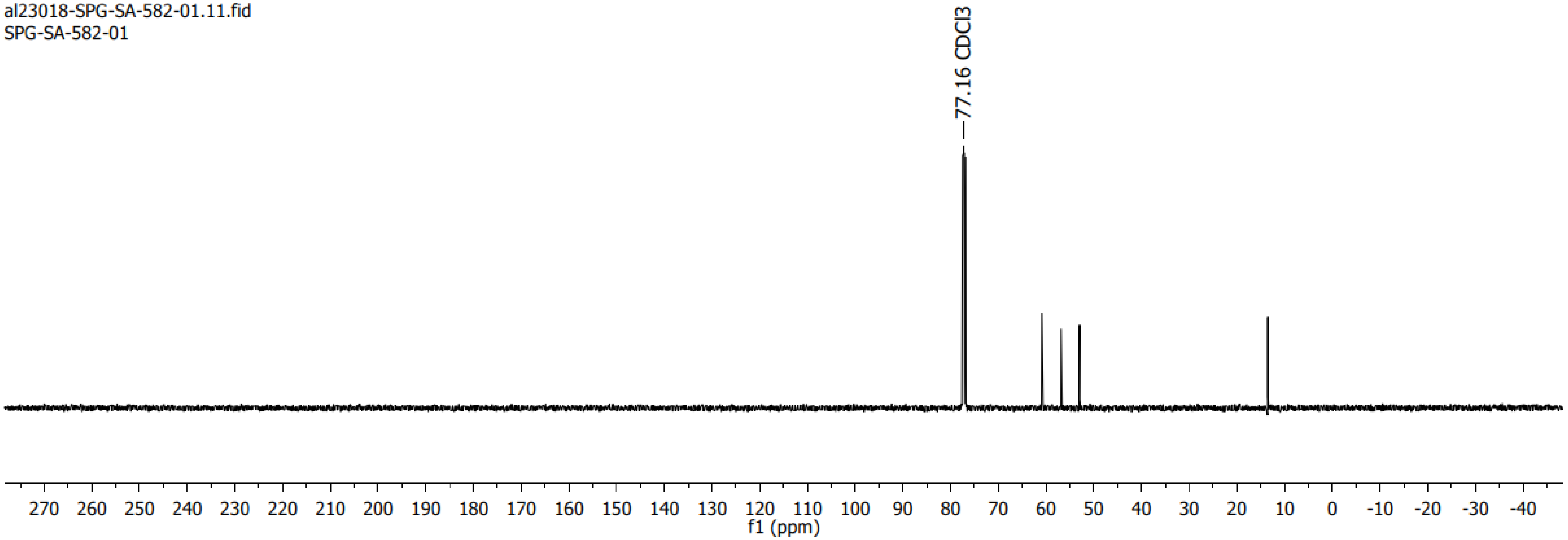

al23018-SPG-SA-582-01.11.fid
SPG-SA-582-01
77.16 CDCl3
270 260 250 240 230 220 210 200 190 180 170 160 150 140 130 120 110 100 90 80 70 60 50 40 30 20 10 0 -10 -20 -30 -40
f1 (ppm)

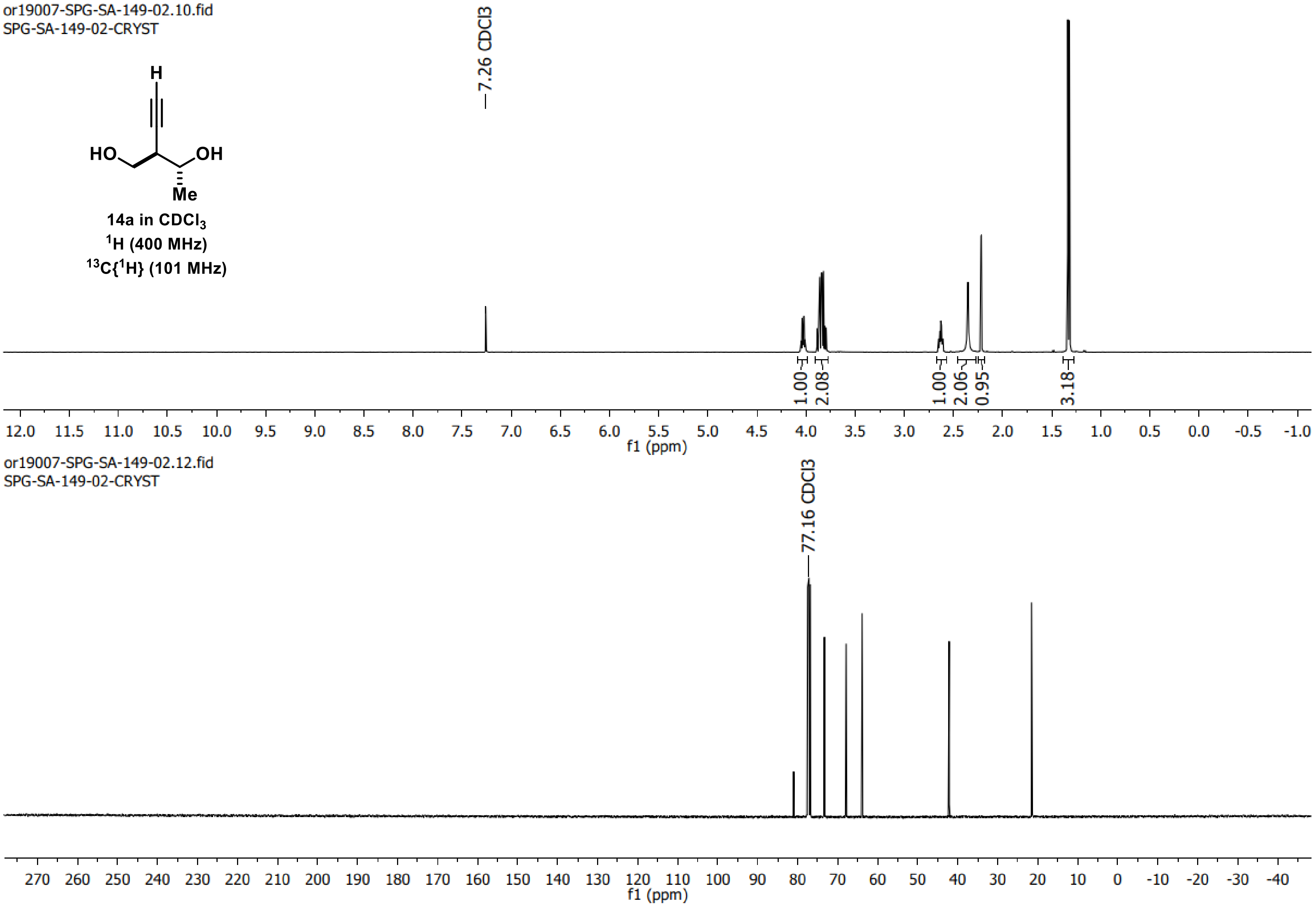
or19007-SPG-SA-149-02.10.fid
SPG-SA-149-02-CRYST
H
HO
OH
Me
14a in CDCl3
1H (400 MHz)
13C{1H} (101 MHz)
7.26 CDCl3
1.00
2.08
1.00
2.06
0.95
3.18
12.0 11.5 11.0 10.5 10.0 9.5 9.0 8.5 8.0 7.5 7.0 6.5 6.0 5.5 5.0 4.5 4.0 3.5 3.0 2.5 2.0 1.5 1.0 0.5 0.0 -0.5 -1.0
f1 (ppm)
or19007-SPG-SA-149-02.12.fid
SPG-SA-149-02-CRYST
77.16 CDCl3
270 260 250 240 230 220 210 200 190 180 170 160 150 140 130 120 110 100 90 80 70 60 50 40 30 20 10 0 -10 -20 -30 -40
f1 (ppm)

jy23032-SPG-SA-371-01.10.fid
SPG-SA-371-01

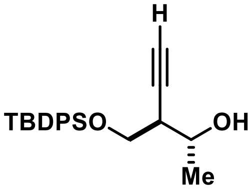

S1 in $CDCl_3$
^{1}H (400 MHz)
$^{13}C\{^{1}H\}$ (101 MHz)

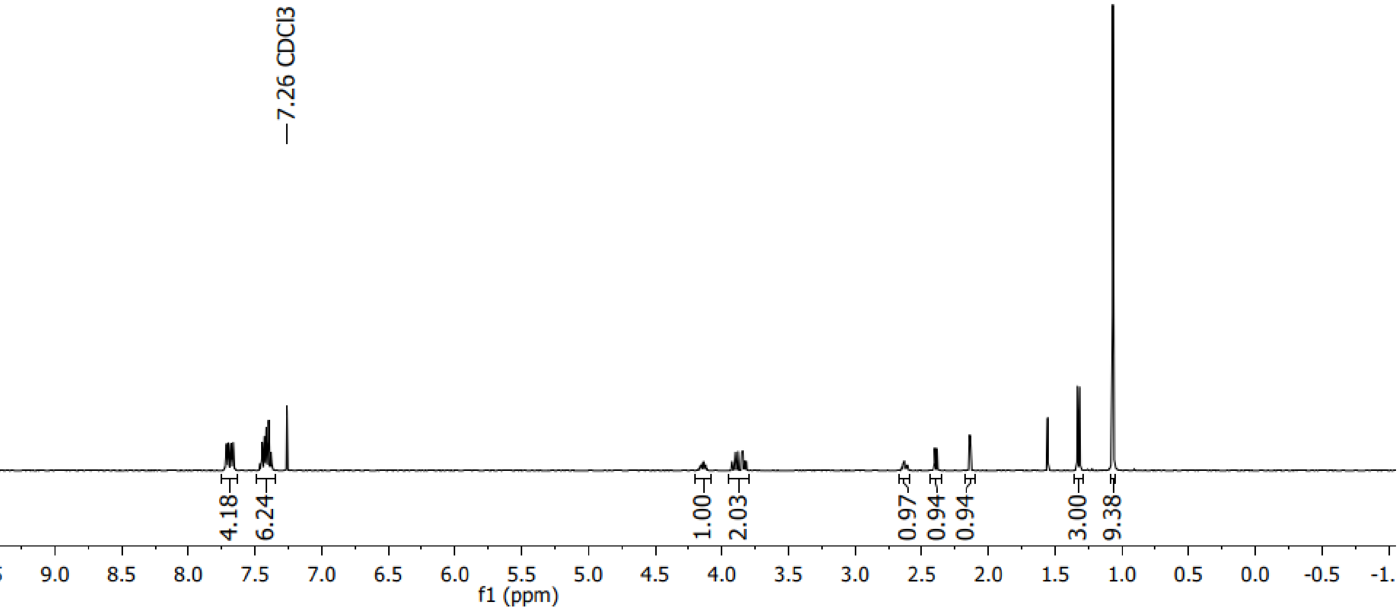

jy23032-SPG-SA-371-01.11.fid
SPG-SA-371-01

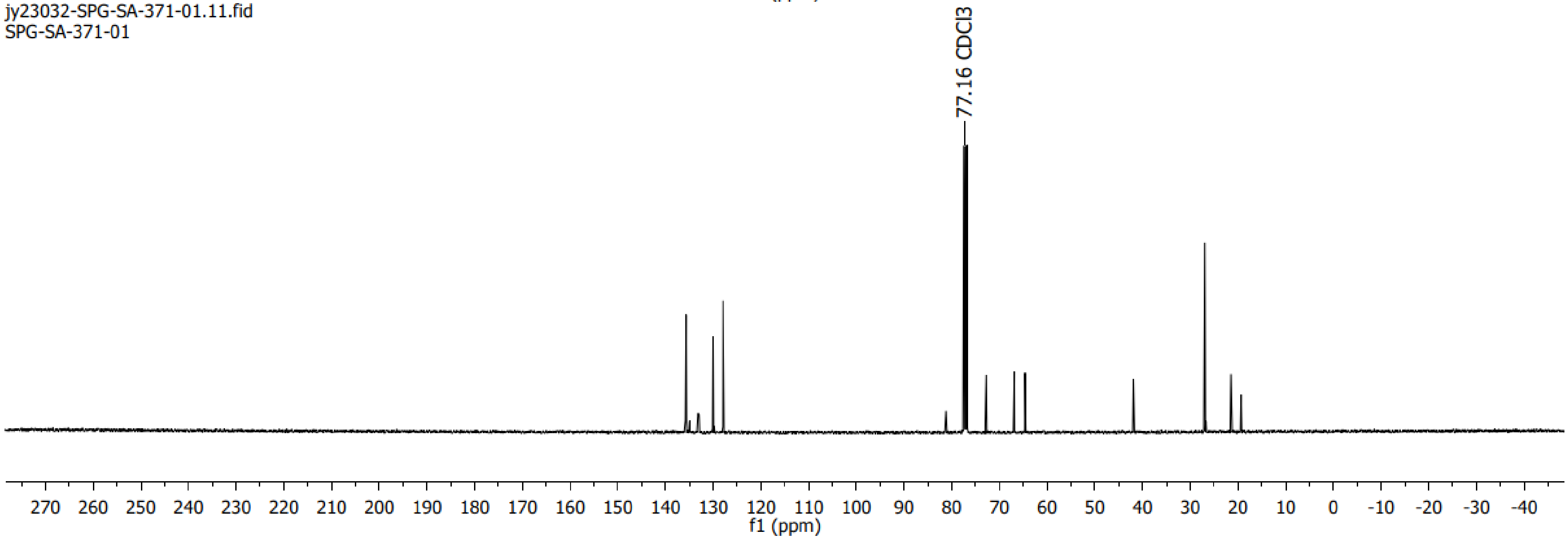

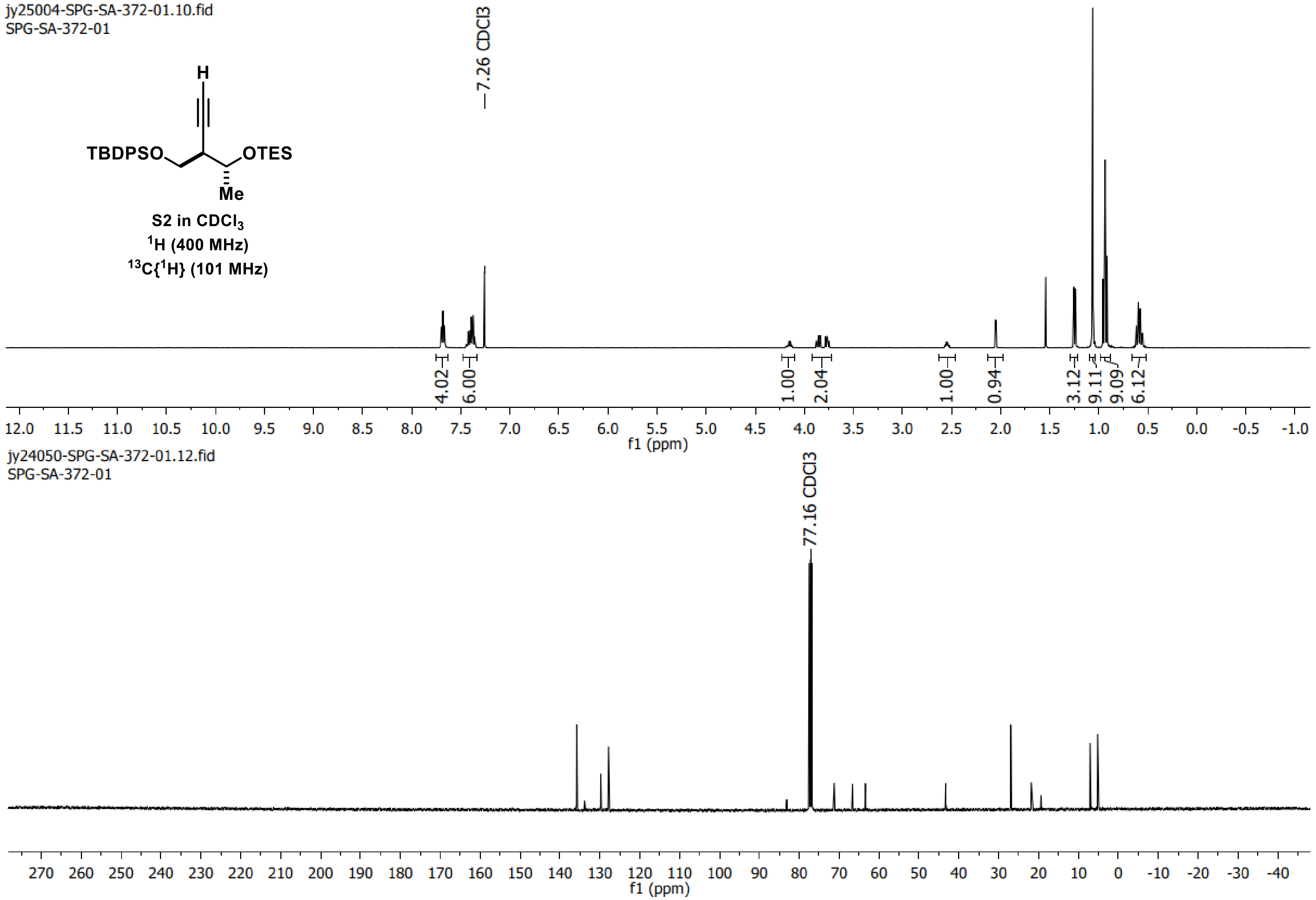
jy25004-SPG-SA-372-01.10.fid
SPG-SA-372-01
H
TBDPSO
OTES
Me
S2 in CDCl3
1H (400 MHz)
13C{1H} (101 MHz)
7.26 CDCl3
4.02
6.00
1.00
2.04
1.00
0.94
3.12
9.11
9.09
6.12
f1 (ppm)
jy24050-SPG-SA-372-01.12.fid
SPG-SA-372-01
77.16 CDCl3
f1 (ppm)

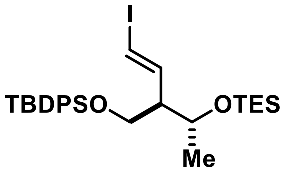

15a in $CDCl_3$
^1H (400 MHz)
$^{13}C\{^1H\}$ (101 MHz)

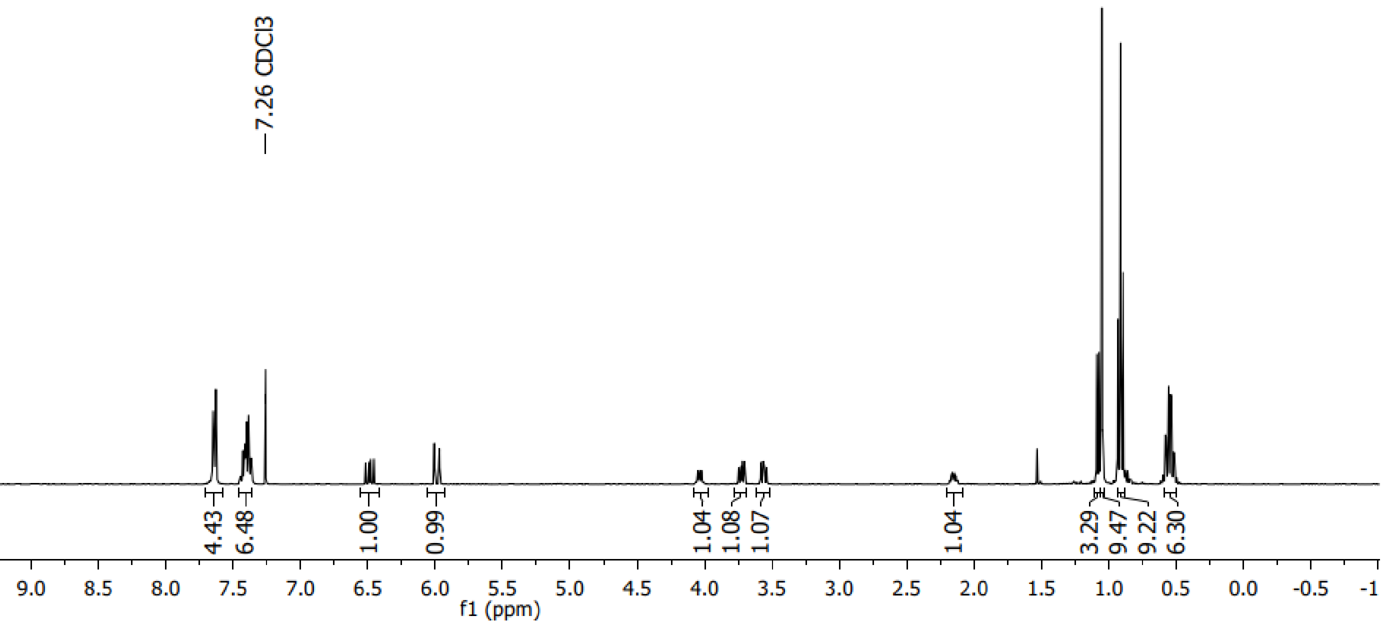

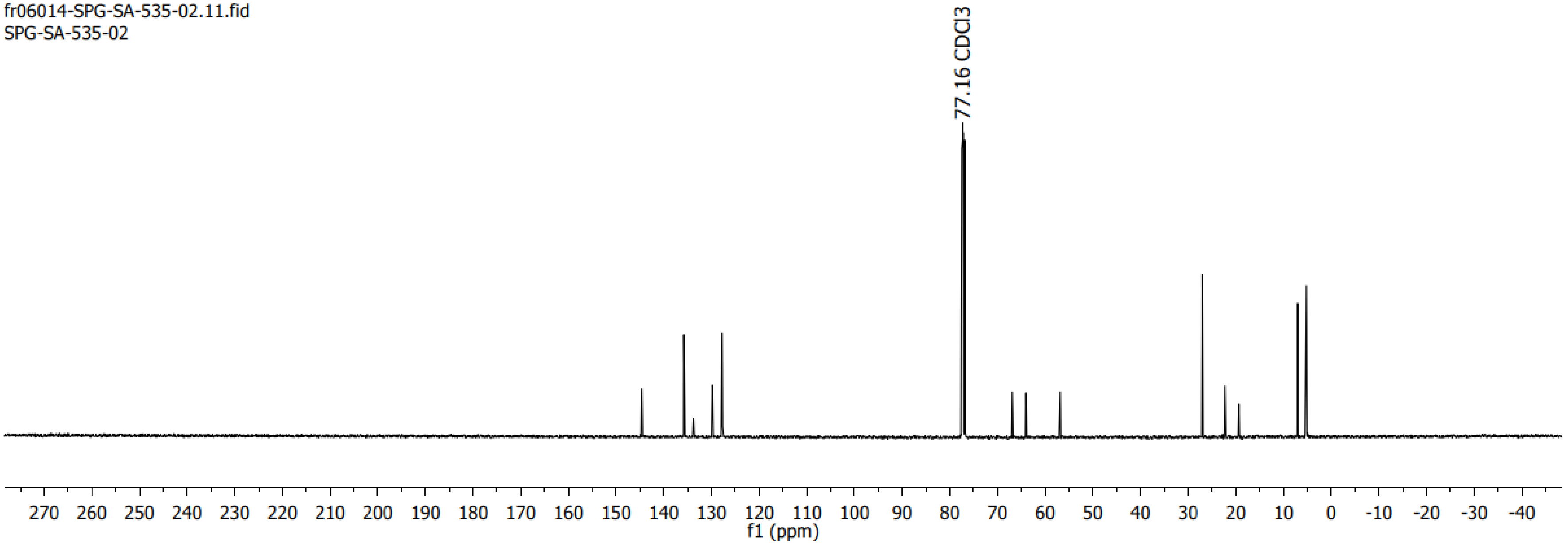

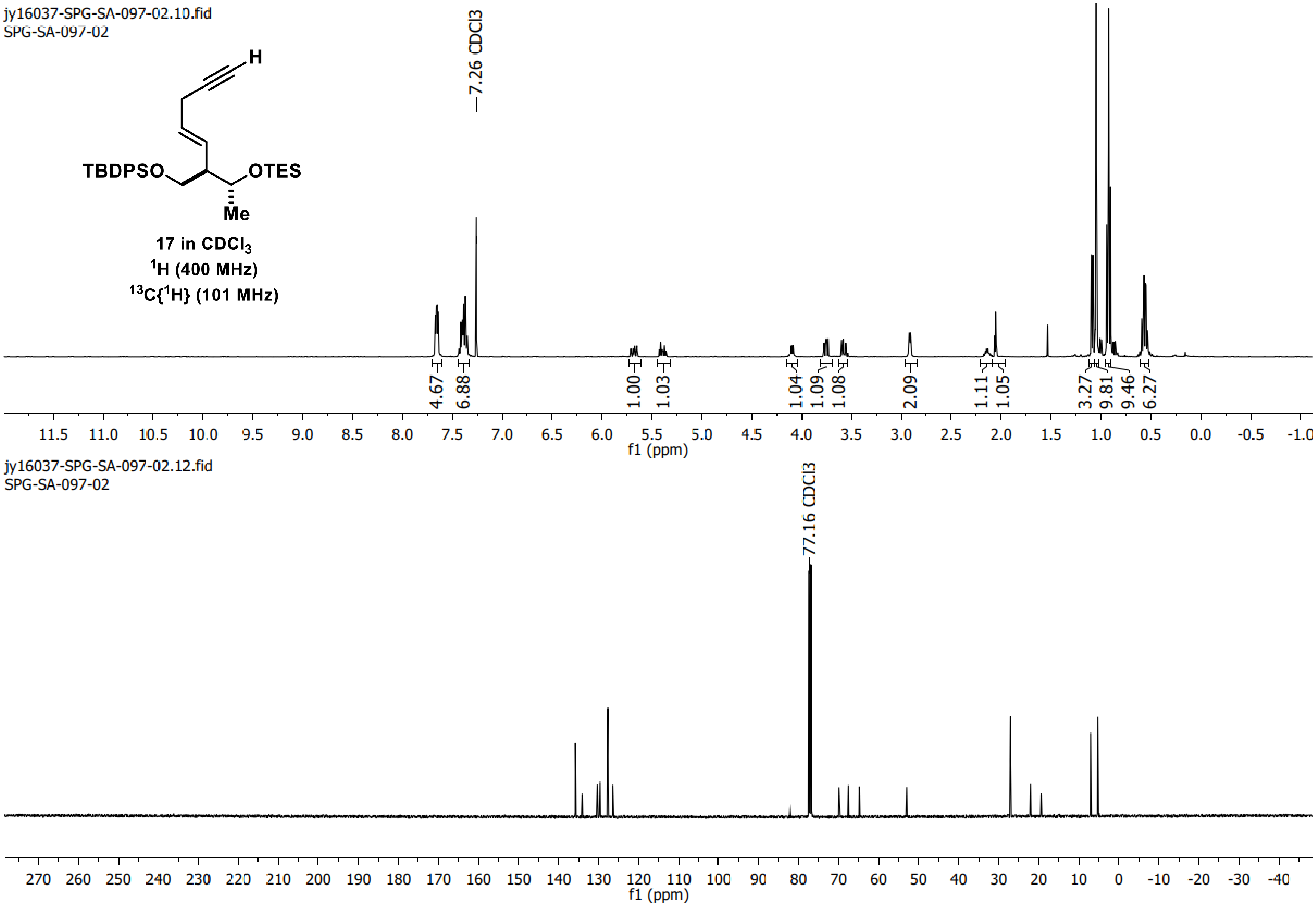
jy16037-SPG-SA-097-02.10.fid
SPG-SA-097-02
H
TBDPSO
OTES
Me
17 in $CDCl_3$
^{1}H (400 MHz)
$^{13}C\{^{1}H\}$ (101 MHz)
7.26 CDCl3
4.67
6.88
1.00
1.03
1.04
1.09
1.08
2.09
1.11
1.05
3.27
9.81
9.46
6.27
11.5 11.0 10.5 10.0 9.5 9.0 8.5 8.0 7.5 7.0 6.5 6.0 5.5 5.0 4.5 4.0 3.5 3.0 2.5 2.0 1.5 1.0 0.5 0.0 -0.5 -1.0
f1 (ppm)
jy16037-SPG-SA-097-02.12.fid
SPG-SA-097-02
77.16 CDCl3
270 260 250 240 230 220 210 200 190 180 170 160 150 140 130 120 110 100 90 80 70 60 50 40 30 20 10 0 -10 -20 -30 -40
f1 (ppm)

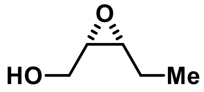

13b in $CDCl_3$
^1H (400 MHz)
$^{13}C\{^1H\}$ (101 MHz)

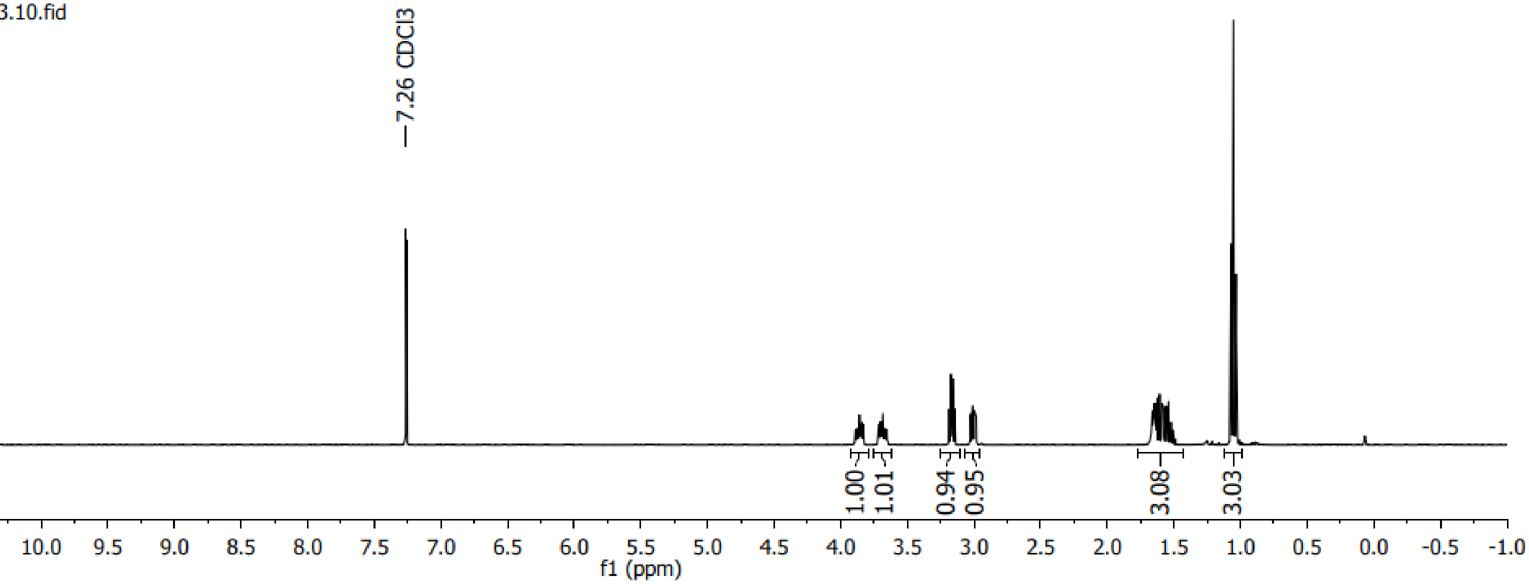

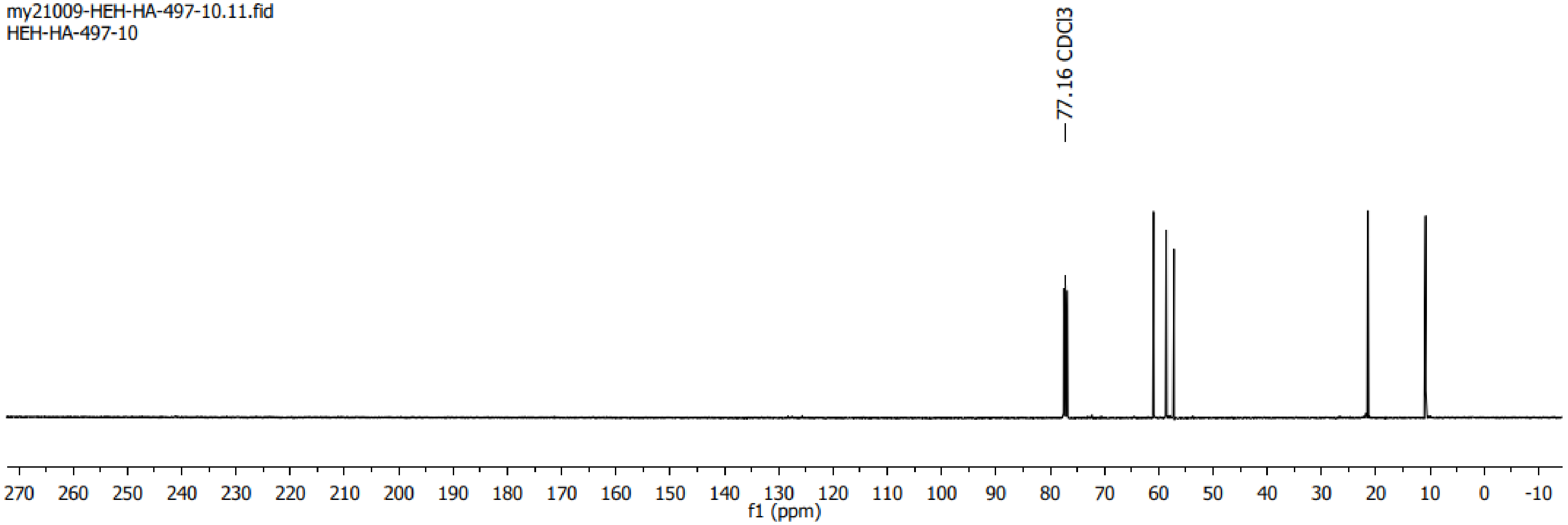

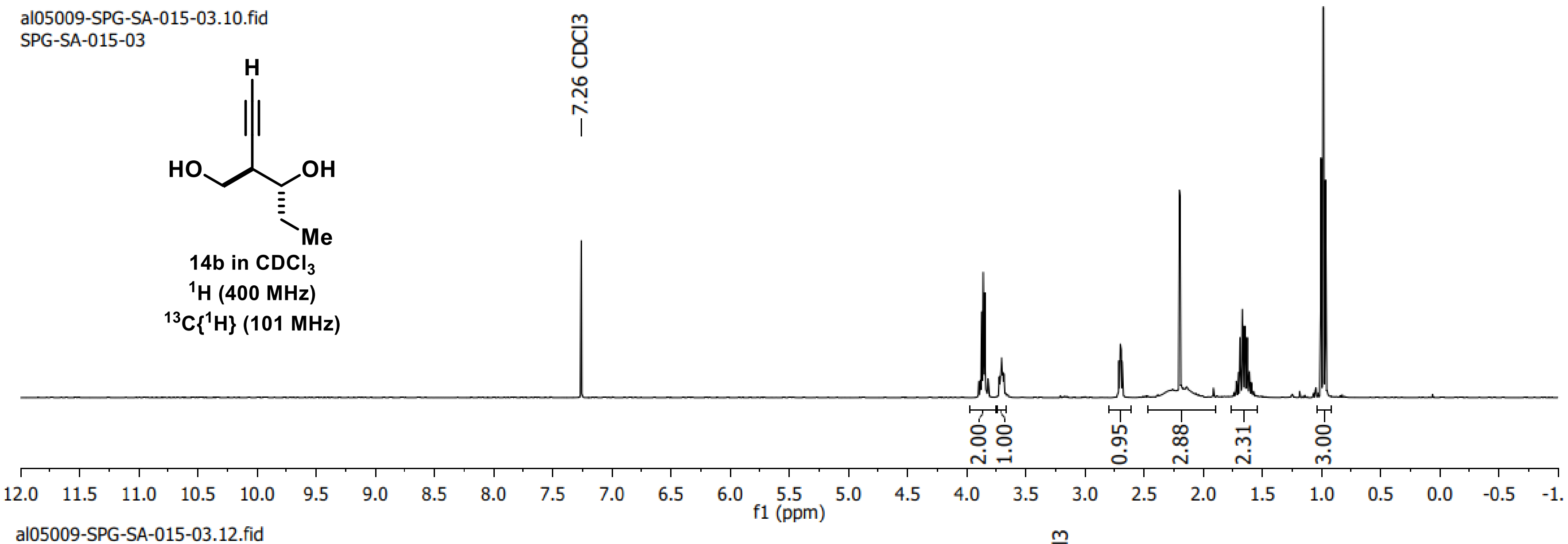
al05009-SPG-SA-015-03.10.fid
SPG-SA-015-03
H
HO
OH
Me
14b in CDCl3
1H (400 MHz)
13C{1H} (101 MHz)
7.26 CDCl3
2.00
1.00
0.95
2.88
2.31
3.00
12.0
11.5
11.0
10.5
10.0
9.5
9.0
8.5
8.0
7.5
7.0
6.5
6.0
5.5
5.0
4.5
4.0
3.5
3.0
2.5
2.0
1.5
1.0
0.5
0.0
-0.5
f1 (ppm)

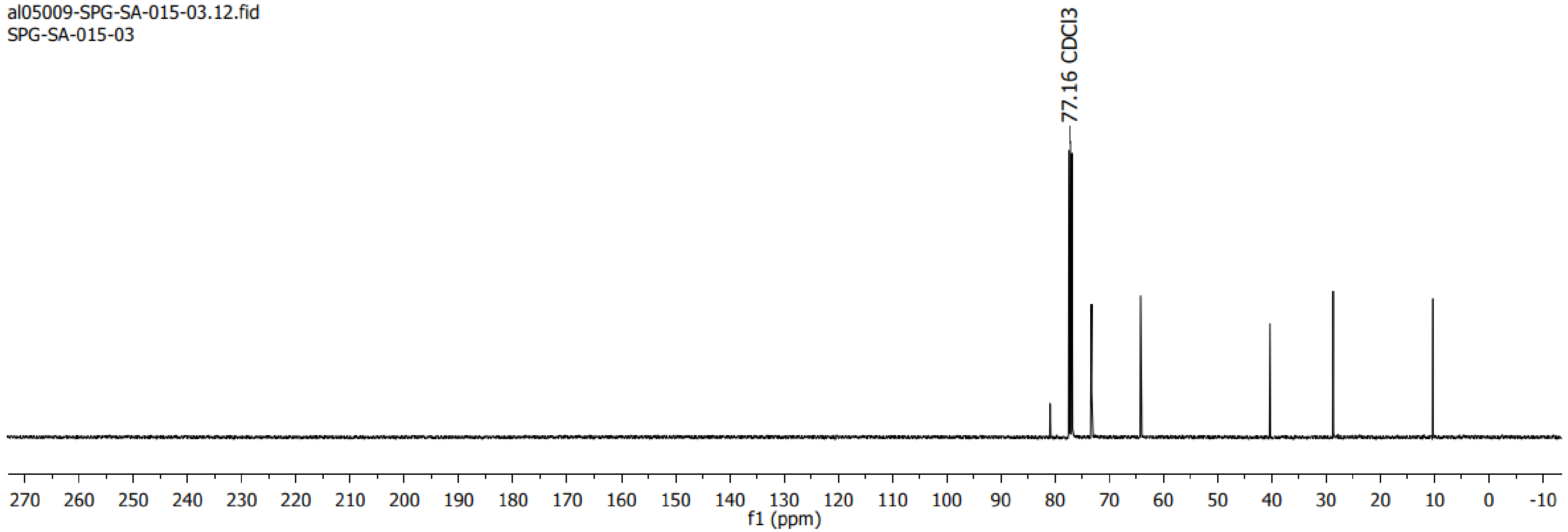
al05009-SPG-SA-015-03.12.fid
SPG-SA-015-03
77.16 CDCl3
270
260
250
240
230
220
210
200
190
180
170
160
150
140
130
120
110
100
90
80
70
60
50
40
30
20
10
0
-10
f1 (ppm)

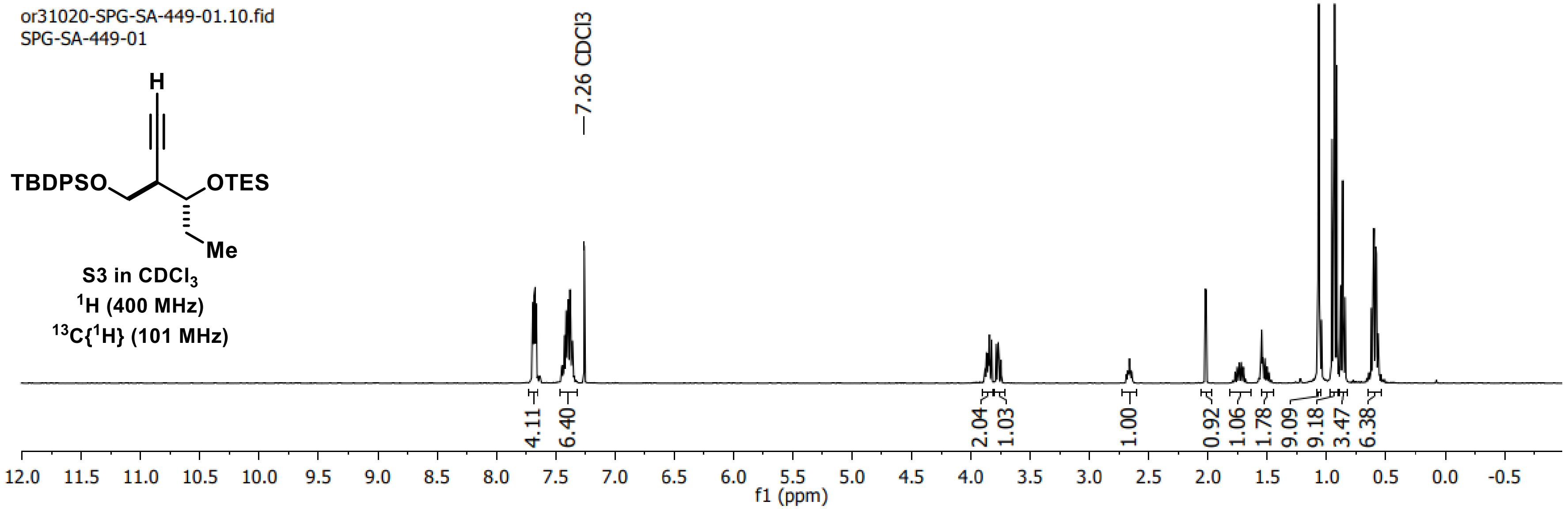

or31020-SPG-SA-449-01.10.fid
SPG-SA-449-01
H
TBDPSO
OTES
Me
S3 in CDCl3
1H (400 MHz)
13C{1H} (101 MHz)
7.26 CDCl3
4.11
6.40
2.04
1.03
1.00
0.92
1.06
1.78
9.09
9.18
3.47
6.38
f1 (ppm)

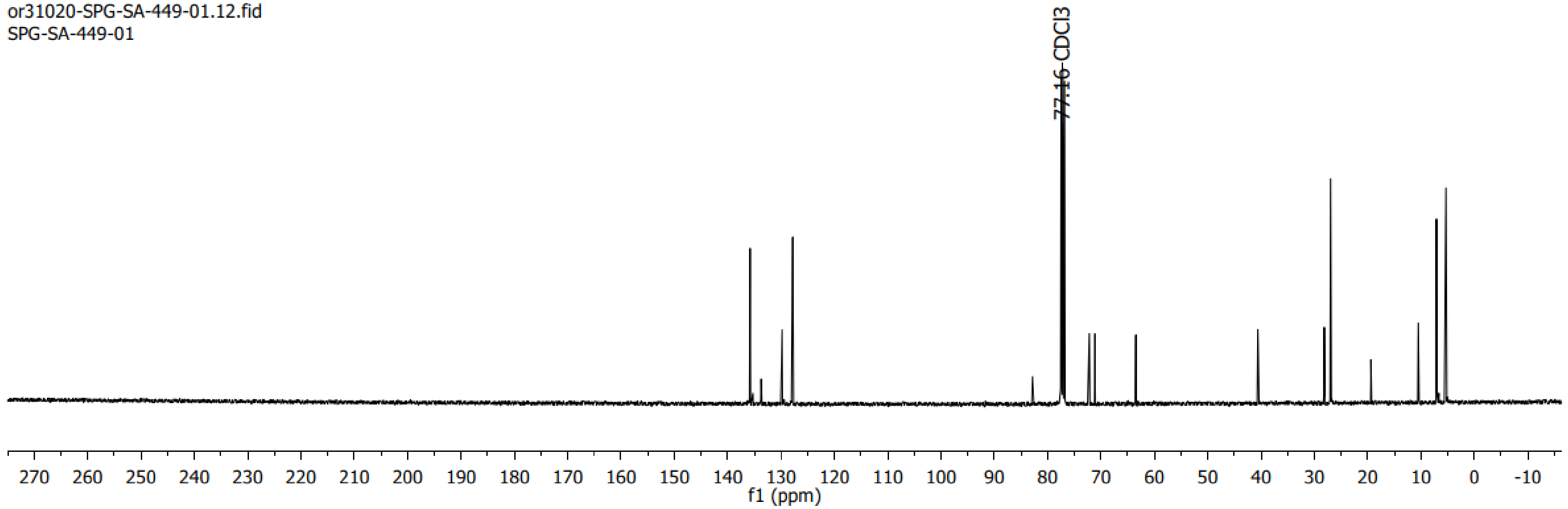

or31020-SPG-SA-449-01.12.fid
SPG-SA-449-01
77.16 CDCl3
f1 (ppm)

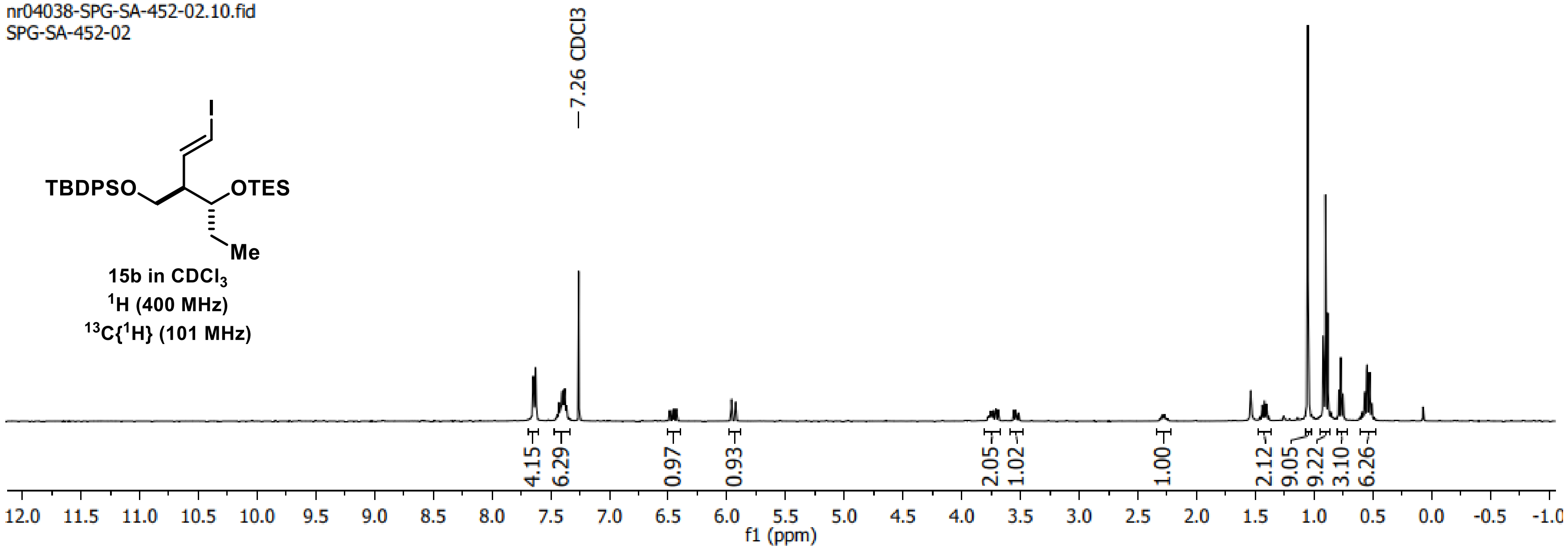

15b in $CDCl_3$
^1H (400 MHz)
$^{13}C\{^1H\}$ (101 MHz)

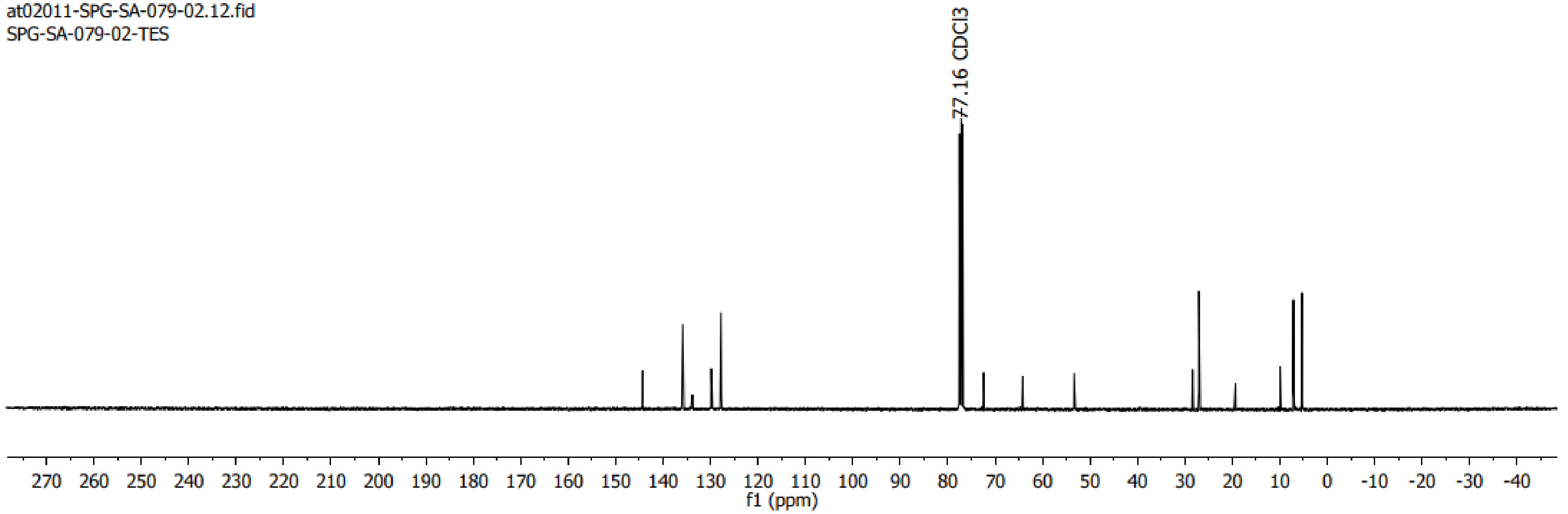

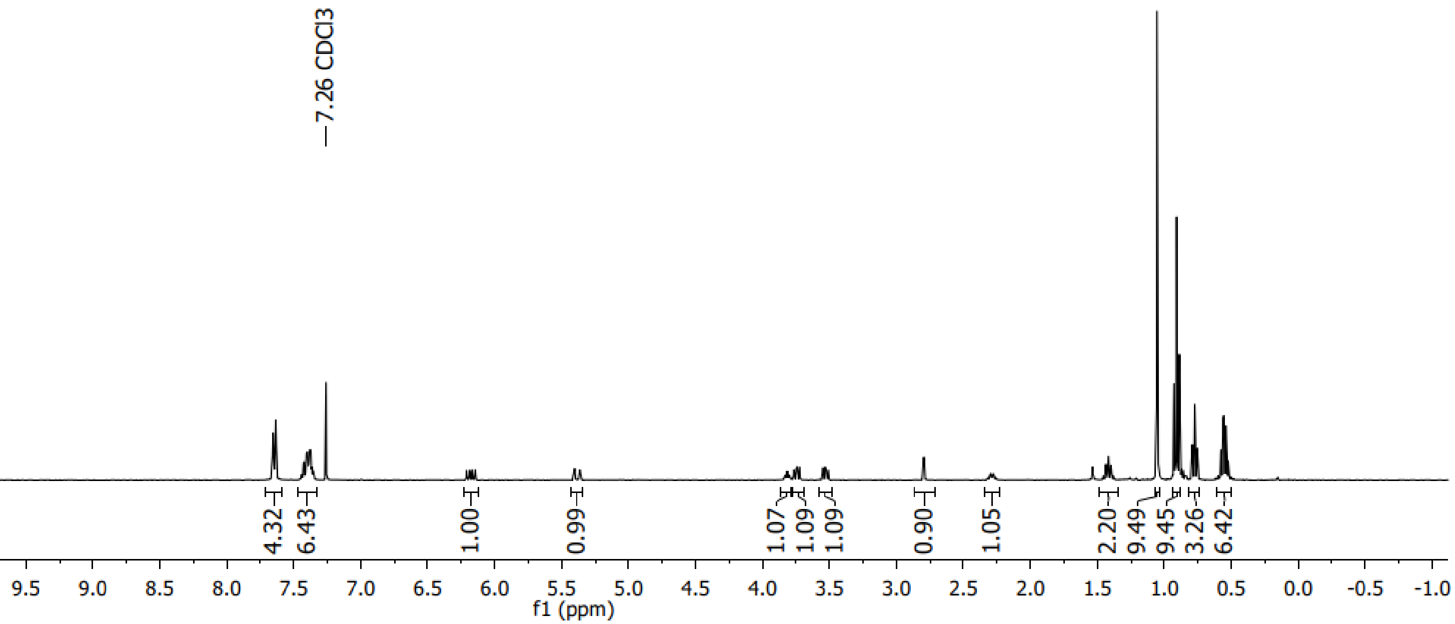

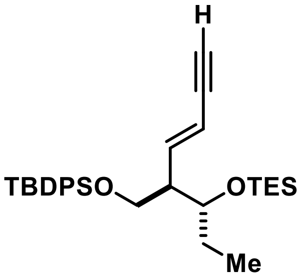

16 in $CDCl_3$
^1H (400 MHz)
$^{13}C\{^1H\}$ (101 MHz)

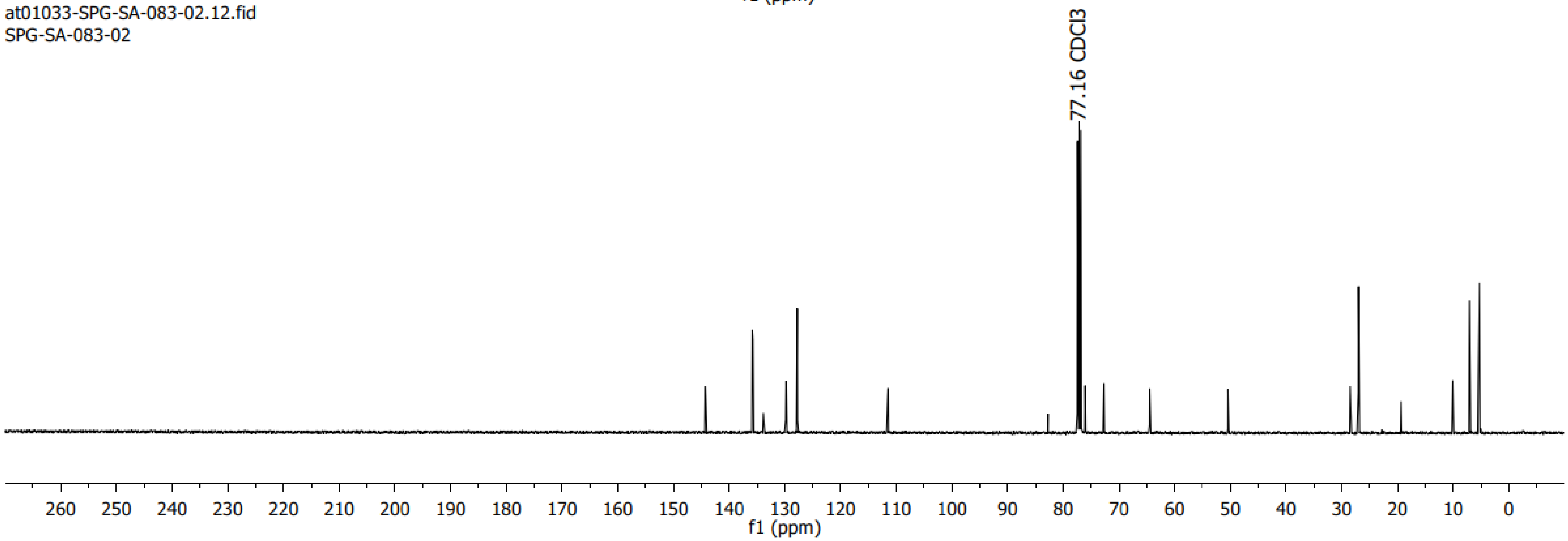

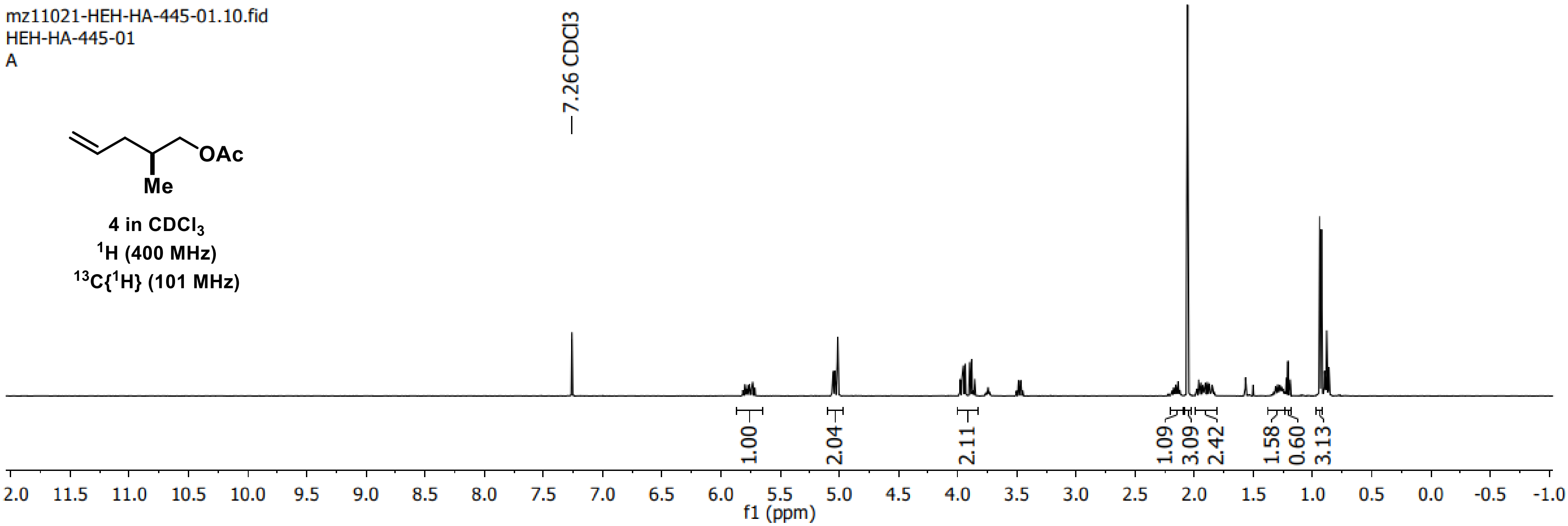
mz11021-HEH-HA-445-01.10.fid
HEH-HA-445-01
A
OAc
Me
4 in $CDCl_3$
^1H (400 MHz)
$^{13}C\{^1H\}$ (101 MHz)
7.26 CDCl3
1.00
2.04
2.11
1.09
3.09
2.42
1.58
0.60
3.13
f1 (ppm)

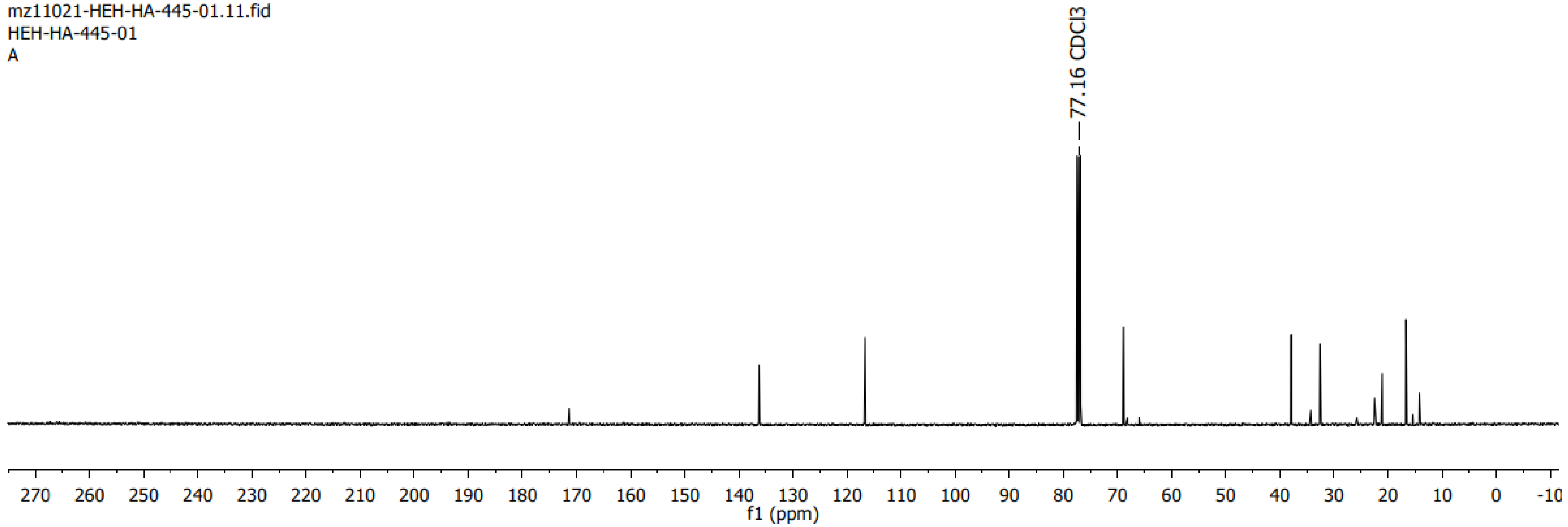
mz11021-HEH-HA-445-01.11.fid
HEH-HA-445-01
A
77.16 CDCl3
f1 (ppm)

mz29044-HEH-HA-470-01.10.fid
HEH-HA-470-01

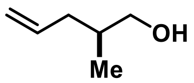

5 in $CDCl_3$
^1H (400 MHz)
$^{13}C\{^1H\}$ (101 MHz)

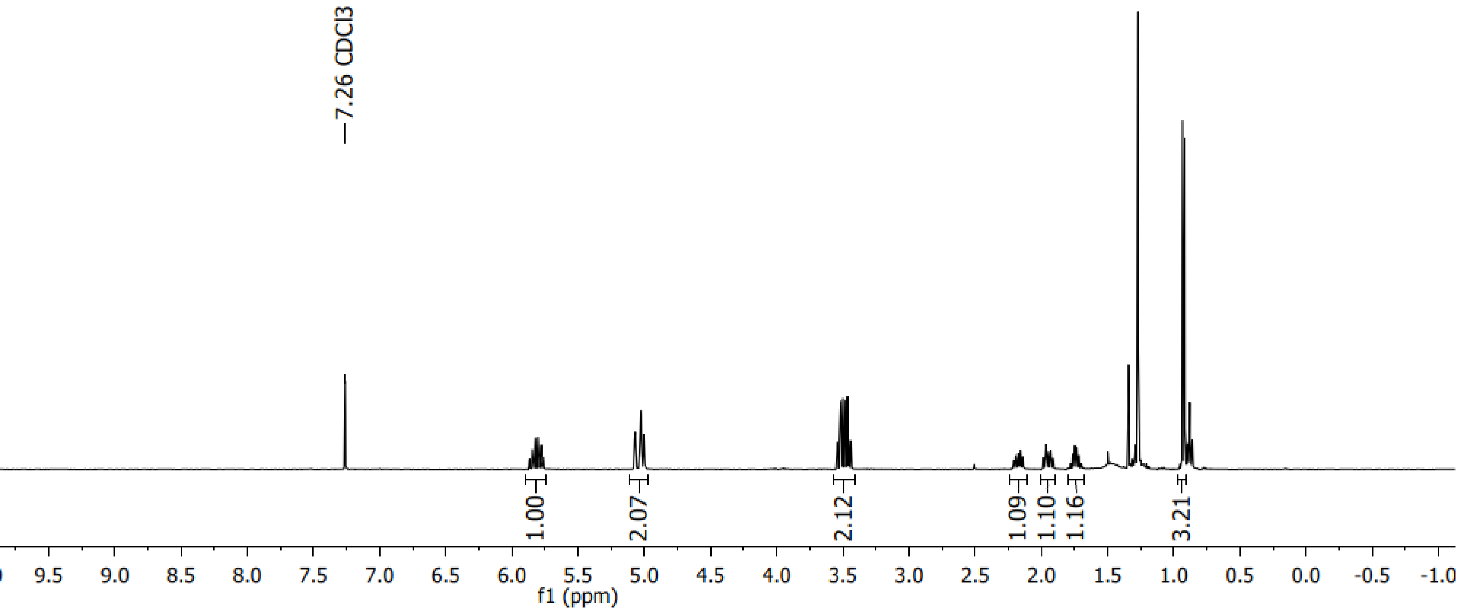

mz29044-HEH-HA-470-01.11.fid
HEH-HA-470-01

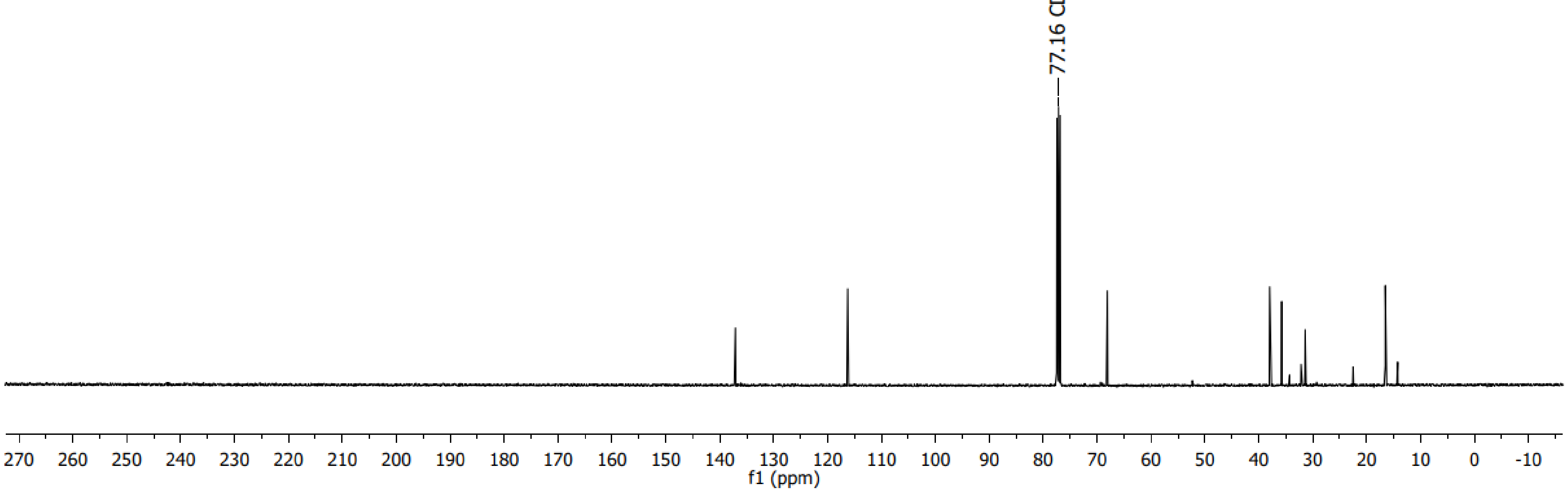

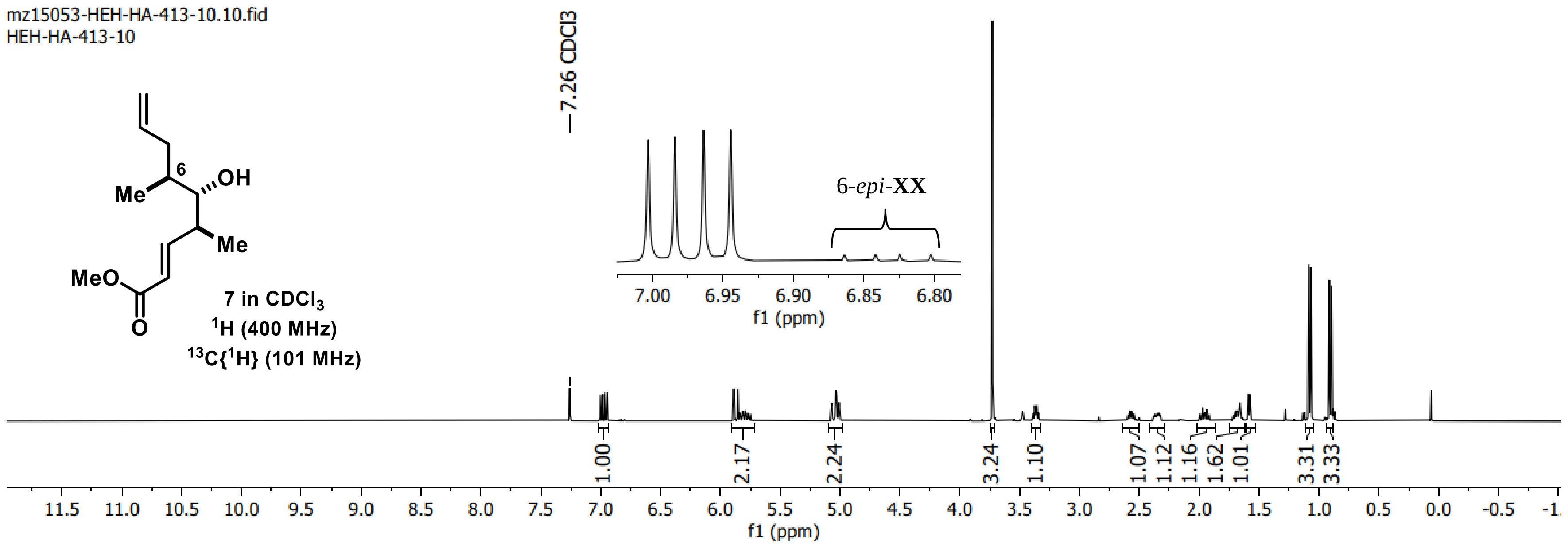
mz15053-HEH-HA-413-10.10.fid
HEH-HA-413-10
Me
6
OH
Me
MeO
O
7 in CDCl3
1H (400 MHz)
13C{1H} (101 MHz)
7.26 CDCl3
6-epi-XX
1.00
2.17
2.24
3.24
1.10
1.07
1.12
1.16
1.62
1.01
3.31
3.33
f1 (ppm)

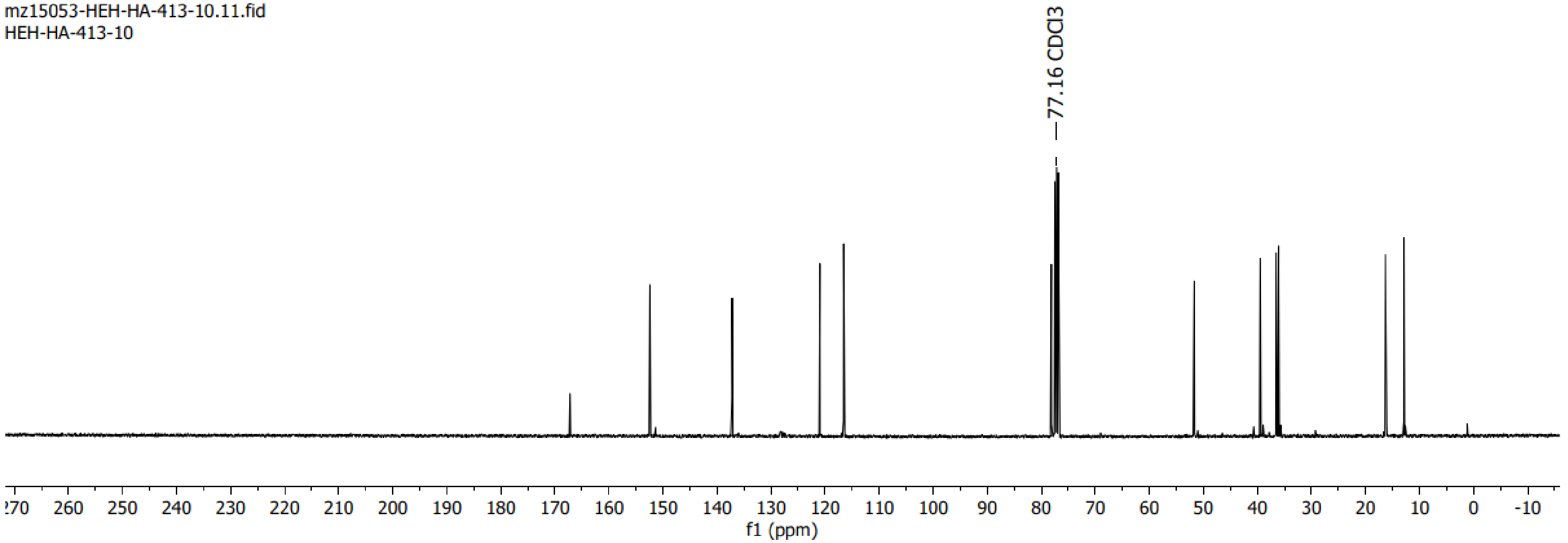
mz15053-HEH-HA-413-10.11.fid
HEH-HA-413-10
77.16 CDCl3
f1 (ppm)

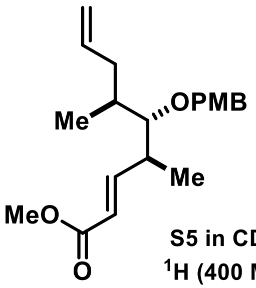

S5 in $CDCl_3$
^1H (400 MHz)
$^{13}C\{^1H\}$ (101 MHz)

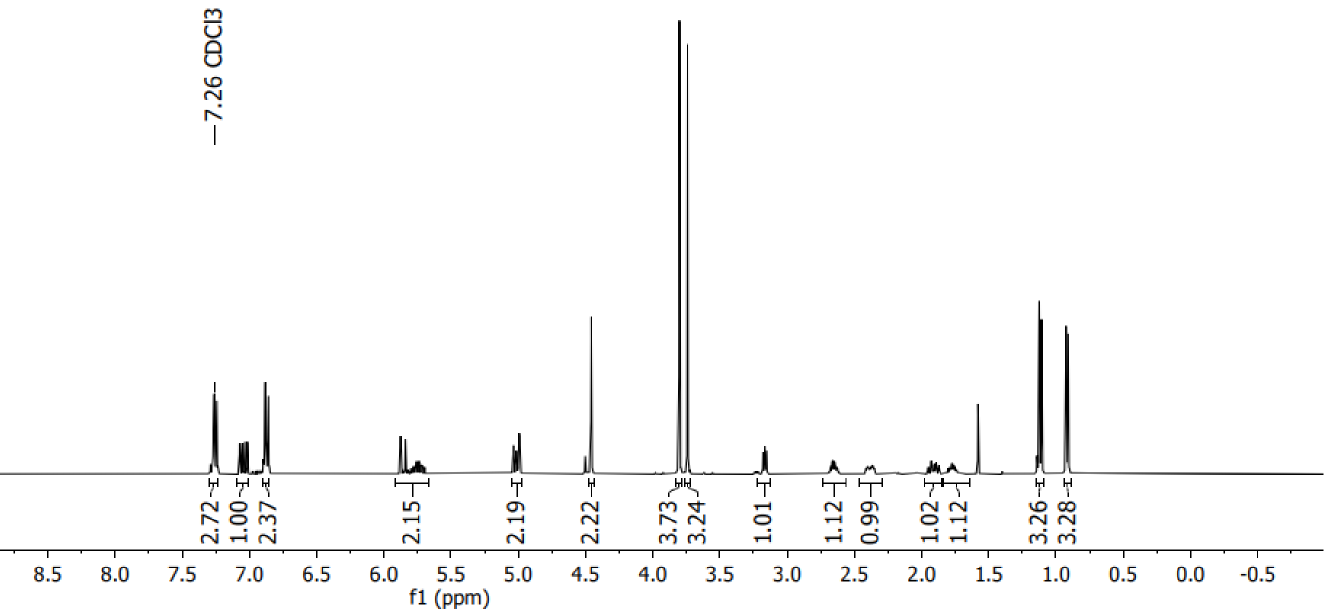

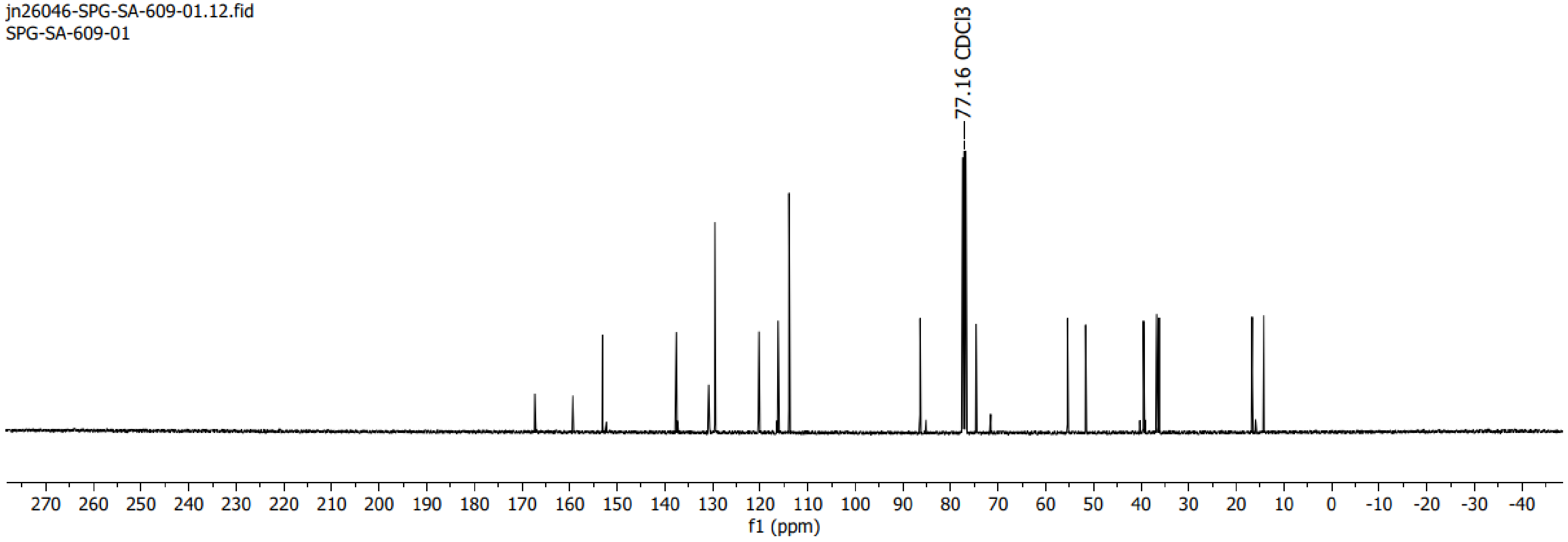

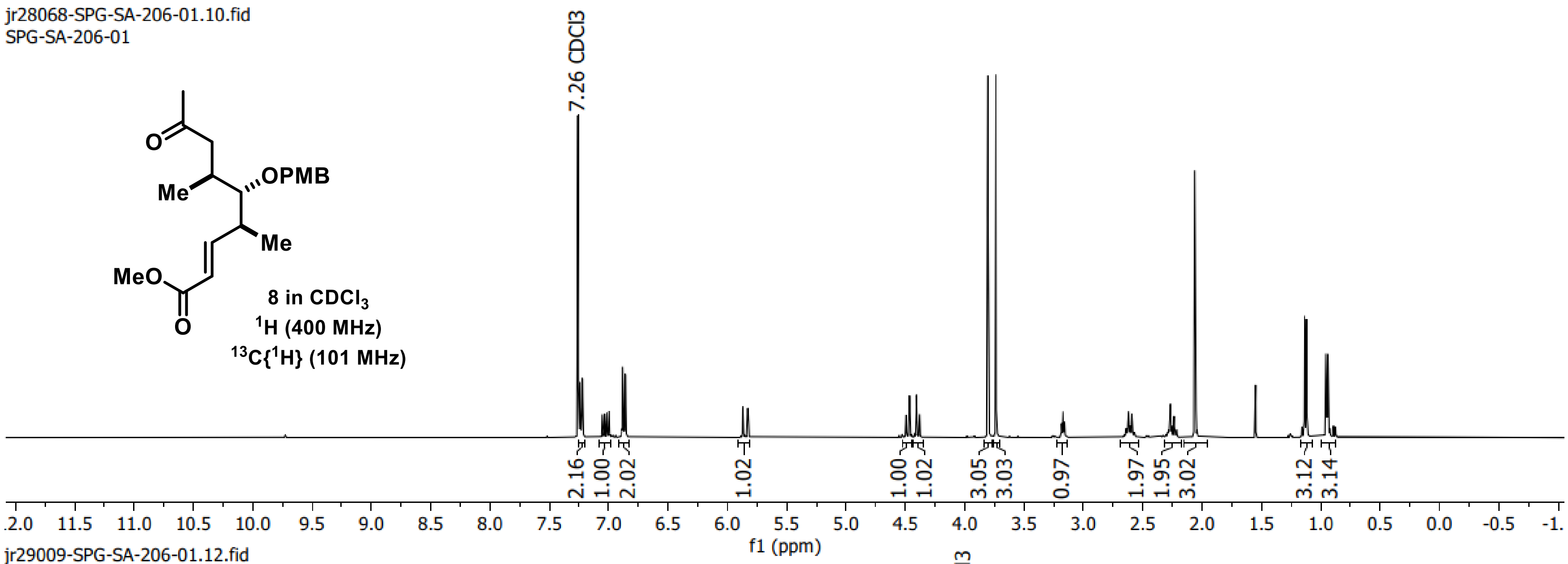
jr28068-SPG-SA-206-01.10.fid
SPG-SA-206-01
7.26 CDCl3
OPMB
Me
Me
MeO
O
O
8 in $CDCl_3$
^{1}H (400 MHz)
$^{13}C\{^{1}H\}$ (101 MHz)
2.16
1.00
2.02
1.02
1.00
1.02
3.05
3.03
0.97
1.97
1.95
3.02
3.12
3.14
f1 (ppm)

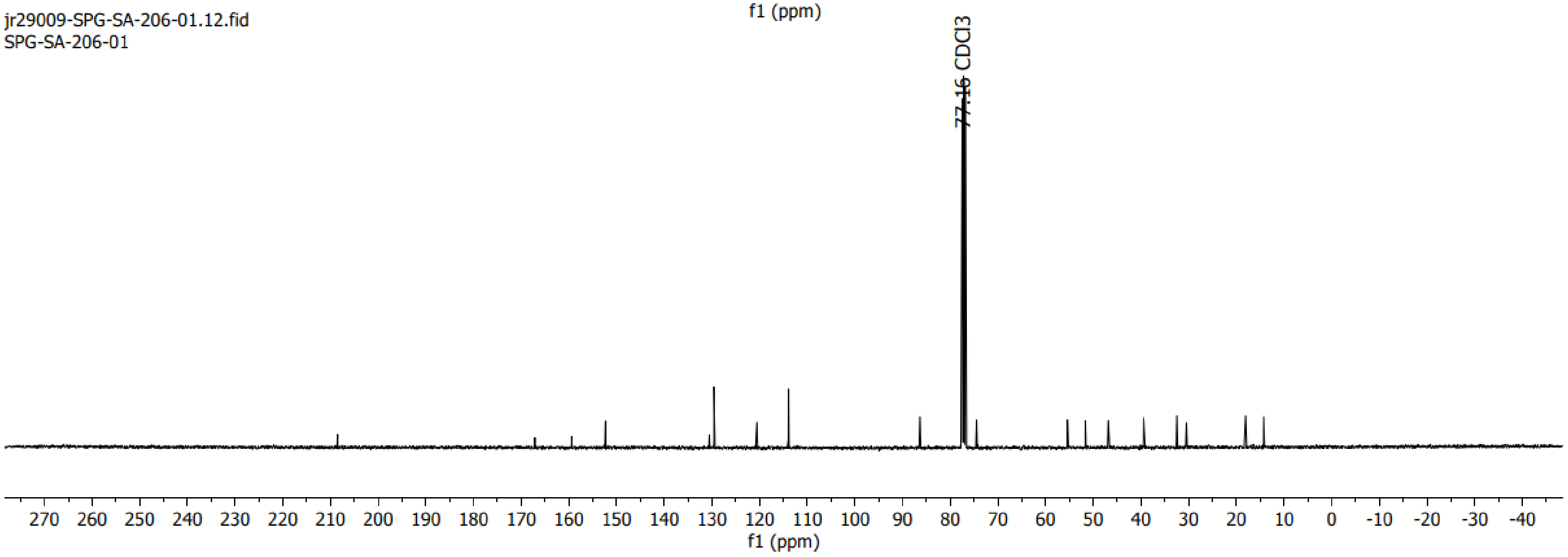
jr29009-SPG-SA-206-01.12.fid
SPG-SA-206-01
77.16 CDCl3
f1 (ppm)

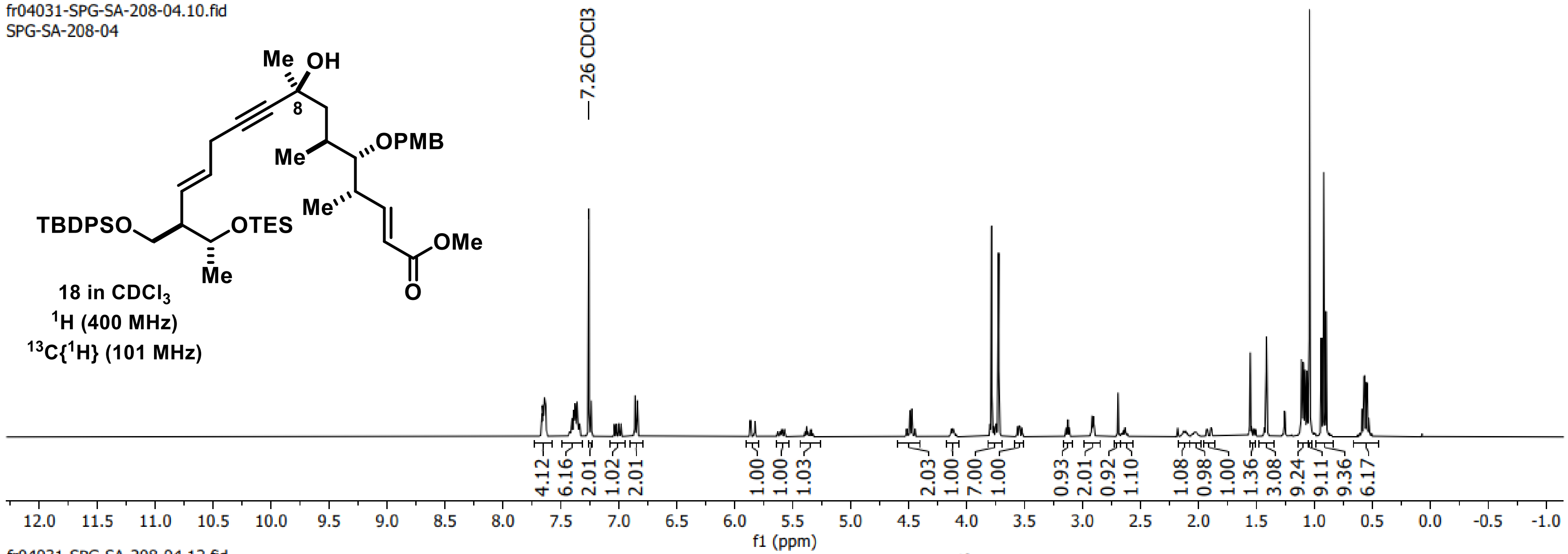
fr04031-SPG-SA-208-04.10.fid
SPG-SA-208-04
Me OH
8
OPMB
Me
Me
TBDPSO
OTES
OMe
Me
O
18 in $CDCl_3$
^1H (400 MHz)
$^{13}C\{^1H\}$ (101 MHz)
7.26 CDCl3
4.12 6.16 2.01 1.02 2.01 1.00 1.00 1.03 2.03 1.00 7.00 1.00 0.93 2.01 0.92 1.10 1.08 0.98 1.00 1.36 3.08 9.24 9.11 9.36 6.17
12.0 11.5 11.0 10.5 10.0 9.5 9.0 8.5 8.0 7.5 7.0 6.5 6.0 5.5 5.0 4.5 4.0 3.5 3.0 2.5 2.0 1.5 1.0 0.5 0.0 -0.5 -1.0
f1 (ppm)

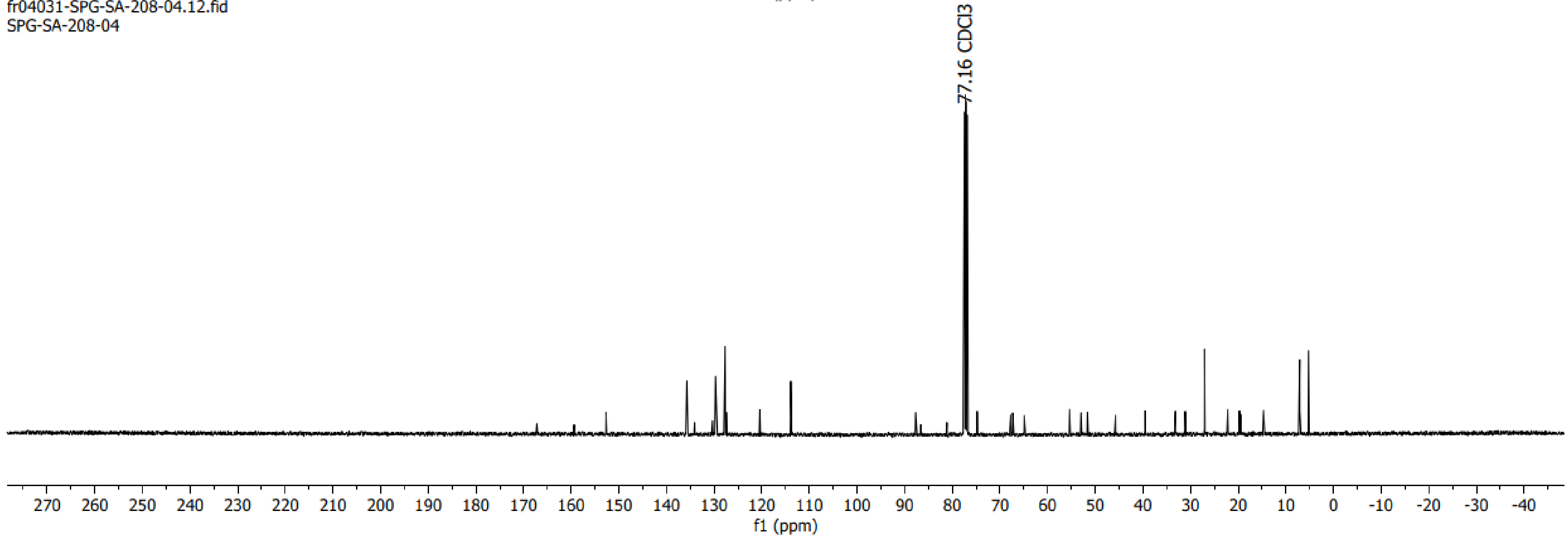
fr04031-SPG-SA-208-04.12.fid
SPG-SA-208-04
77.16 CDCl3
270 260 250 240 230 220 210 200 190 180 170 160 150 140 130 120 110 100 90 80 70 60 50 40 30 20 10 0 -10 -20 -30 -40
f1 (ppm)

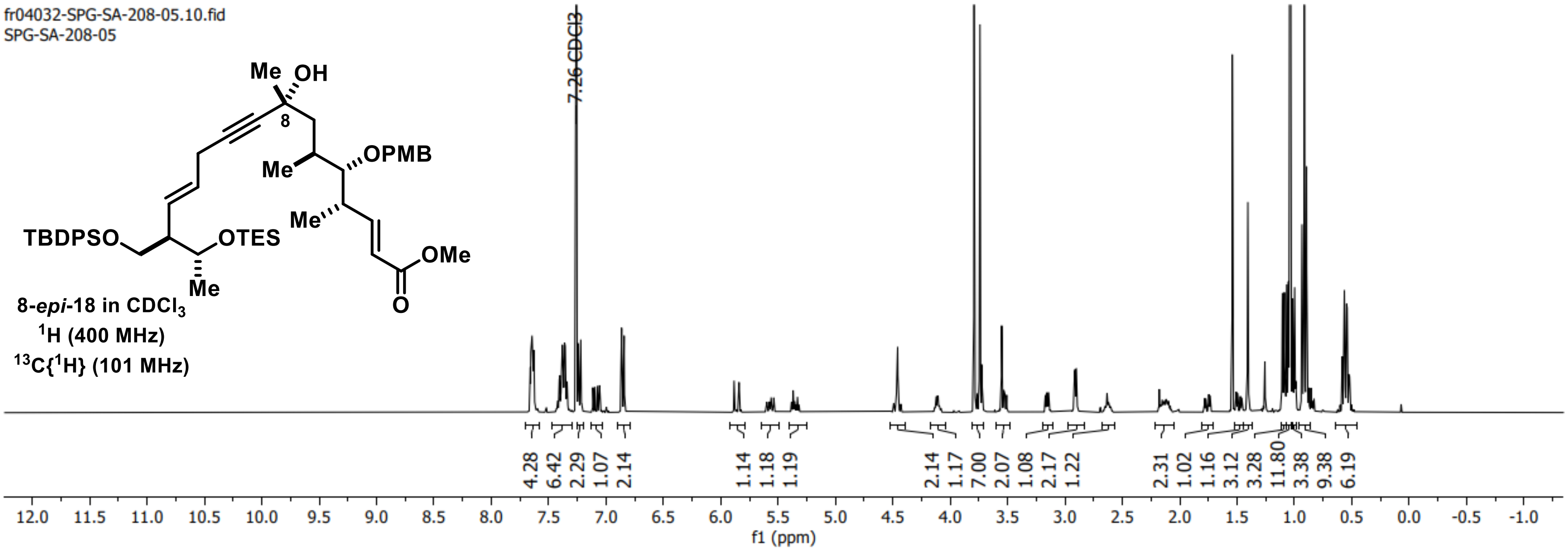
fr04032-SPG-SA-208-05.10.fid
SPG-SA-208-05
Me OH
8
OPMB
Me
Me
TBDPSO
OTES
OMe
Me
O
8-*epi*-18 in $CDCl_3$
^1H (400 MHz)
$^{13}C\{^1H\}$ (101 MHz)
7.26 CDCl3
4.28 6.42 2.29 1.07 2.14 1.14 1.18 1.19 2.14 1.17 7.00 2.07 1.08 2.17 1.22 2.31 1.02 1.16 3.12 3.28 11.80 3.38 9.38 6.19
f1 (ppm)

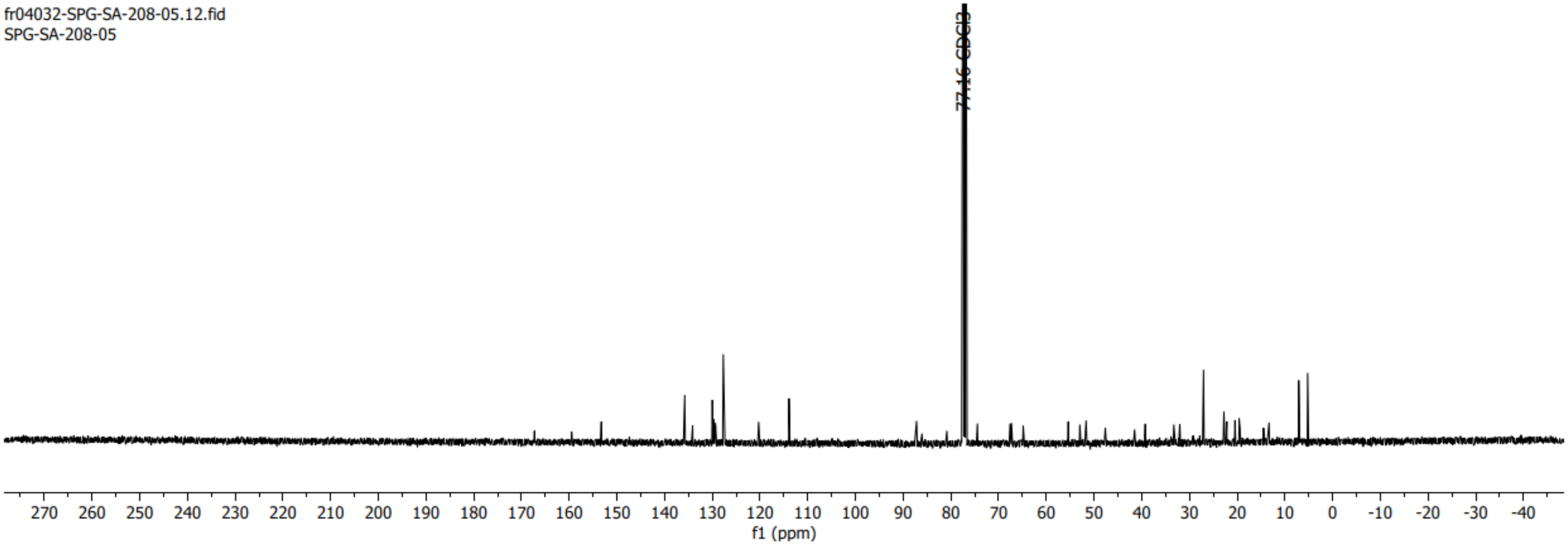
fr04032-SPG-SA-208-05.12.fid
SPG-SA-208-05
77.16 CDCl3
f1 (ppm)

fr07017-SPG-SA-212-01.10.fid
SPG-SA-212-01

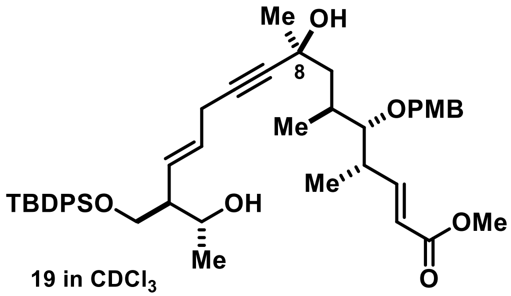

19 in $CDCl_3$
^{1}H (400 MHz)
$^{13}C\{^{1}H\}$ (101 MHz)

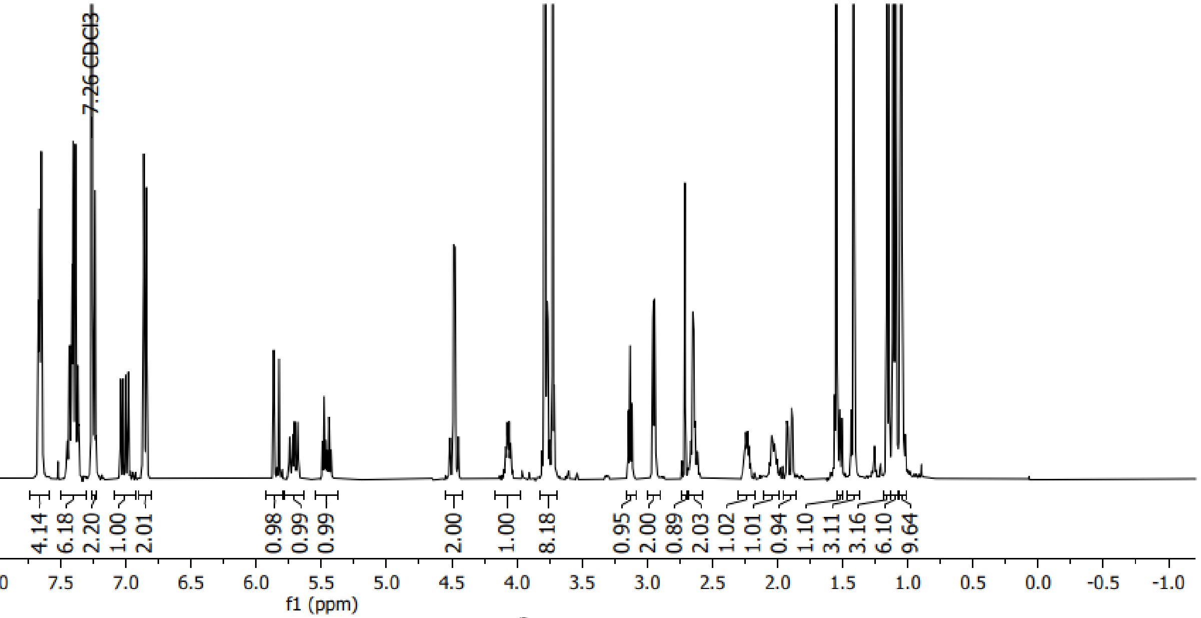

fr07017-SPG-SA-212-01.12.fid
SPG-SA-212-01

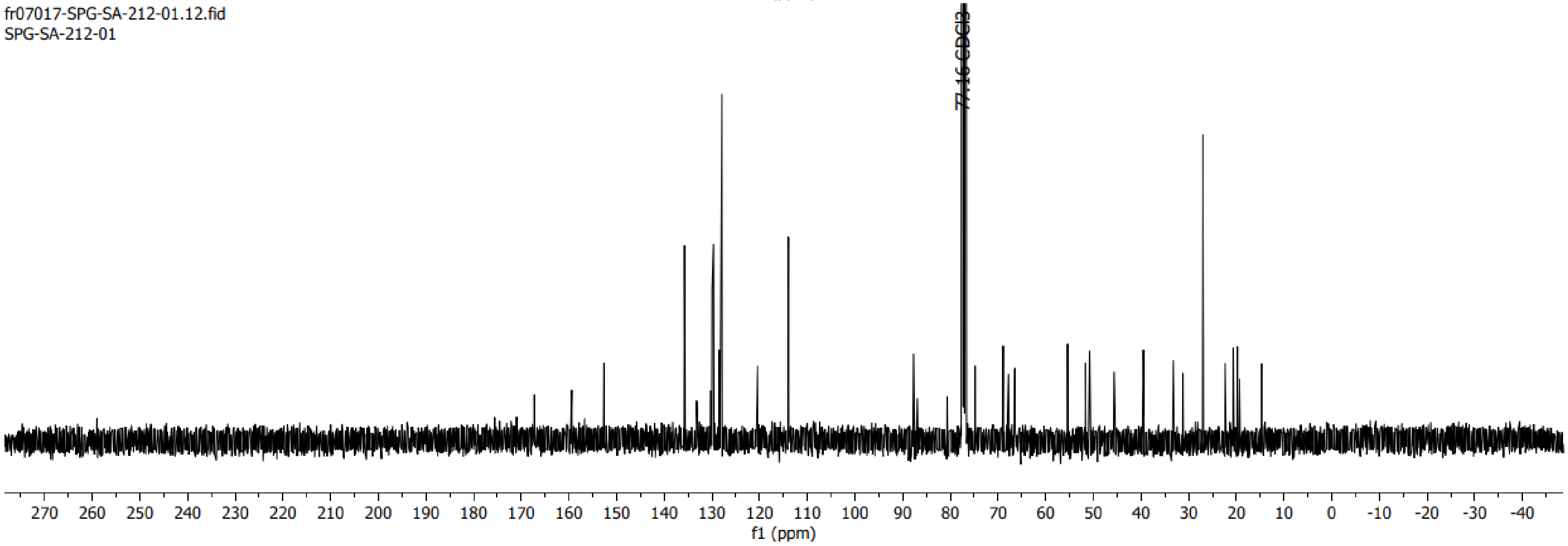

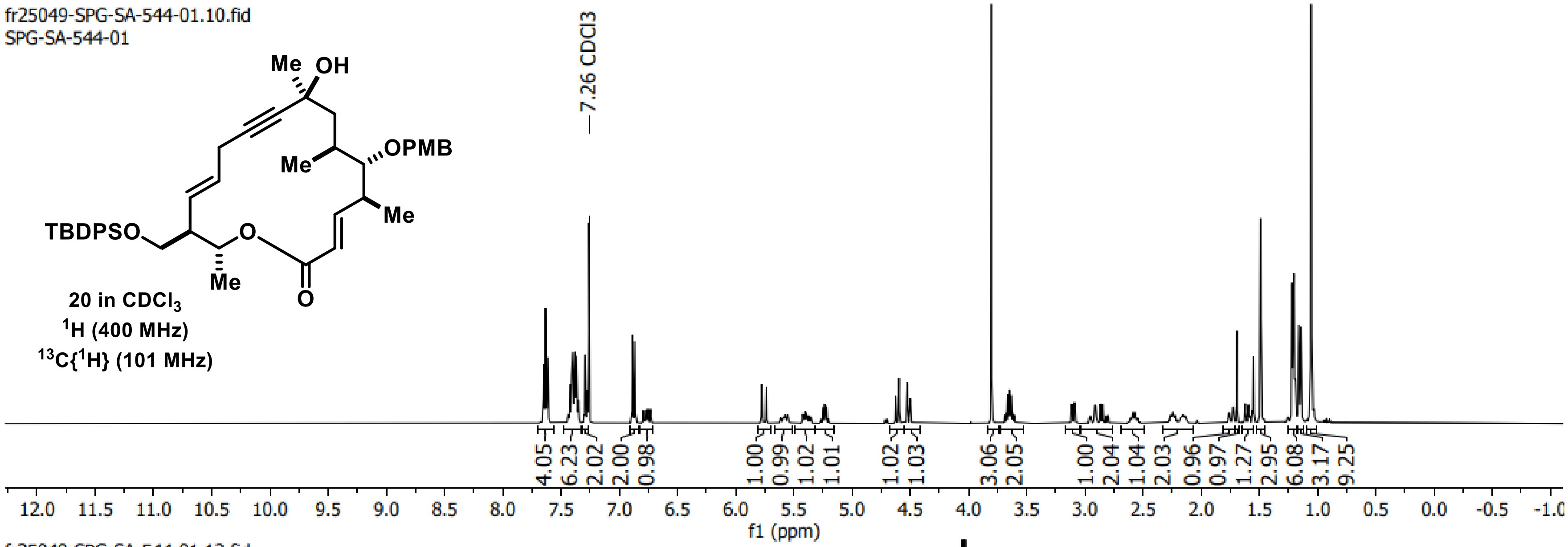

fr25049-SPG-SA-544-01.10.fid
SPG-SA-544-01
Me OH
OPMB
Me
Me
TBDPSO
O
Me
O
20 in $CDCl_3$
^{1}H (400 MHz)
$^{13}C\{^{1}H\}$ (101 MHz)
7.26 CDCl3
4.05 6.23 2.02 2.00 0.98 1.00 0.99 1.02 1.01 1.02 1.03 3.06 2.05 1.00 2.04 1.04 2.03 0.96 0.97 1.27 2.95 6.08 3.17 9.25
12.0 11.5 11.0 10.5 10.0 9.5 9.0 8.5 8.0 7.5 7.0 6.5 6.0 5.5 5.0 4.5 4.0 3.5 3.0 2.5 2.0 1.5 1.0 0.5 0.0 -0.5 -1.0
f1 (ppm)

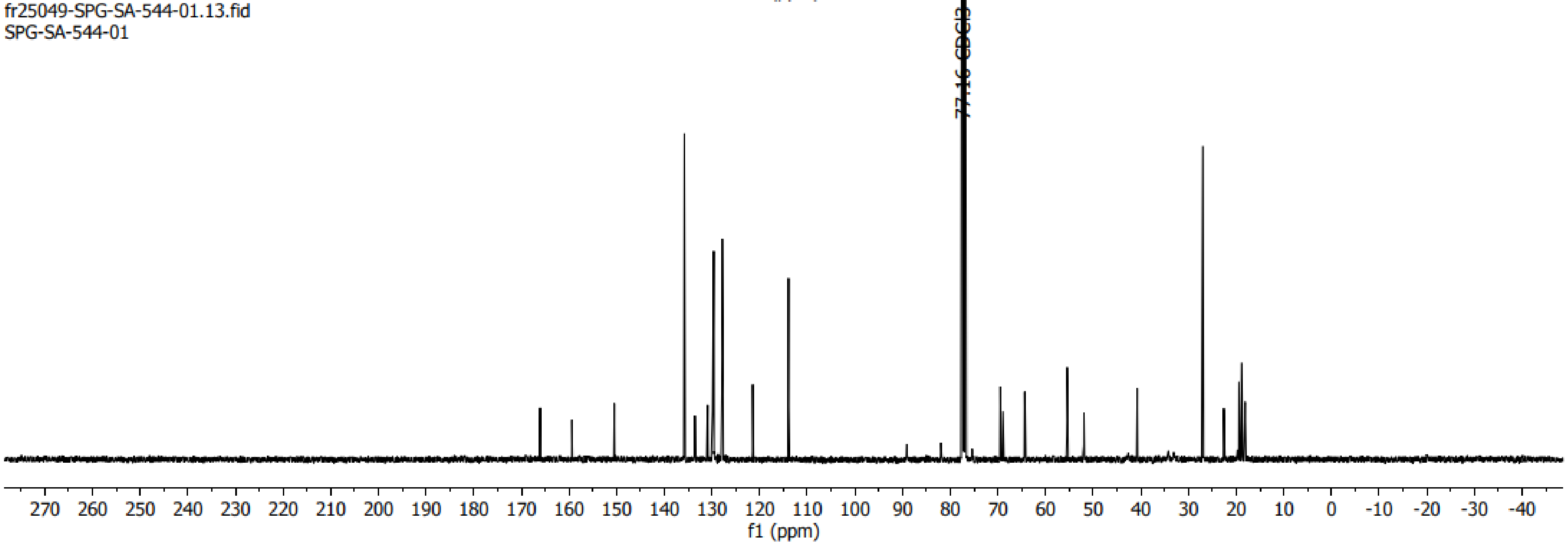

fr25049-SPG-SA-544-01.13.fid
SPG-SA-544-01
77.16 CDCl3
270 260 250 240 230 220 210 200 190 180 170 160 150 140 130 120 110 100 90 80 70 60 50 40 30 20 10 0 -10 -20 -30 -40
f1 (ppm)

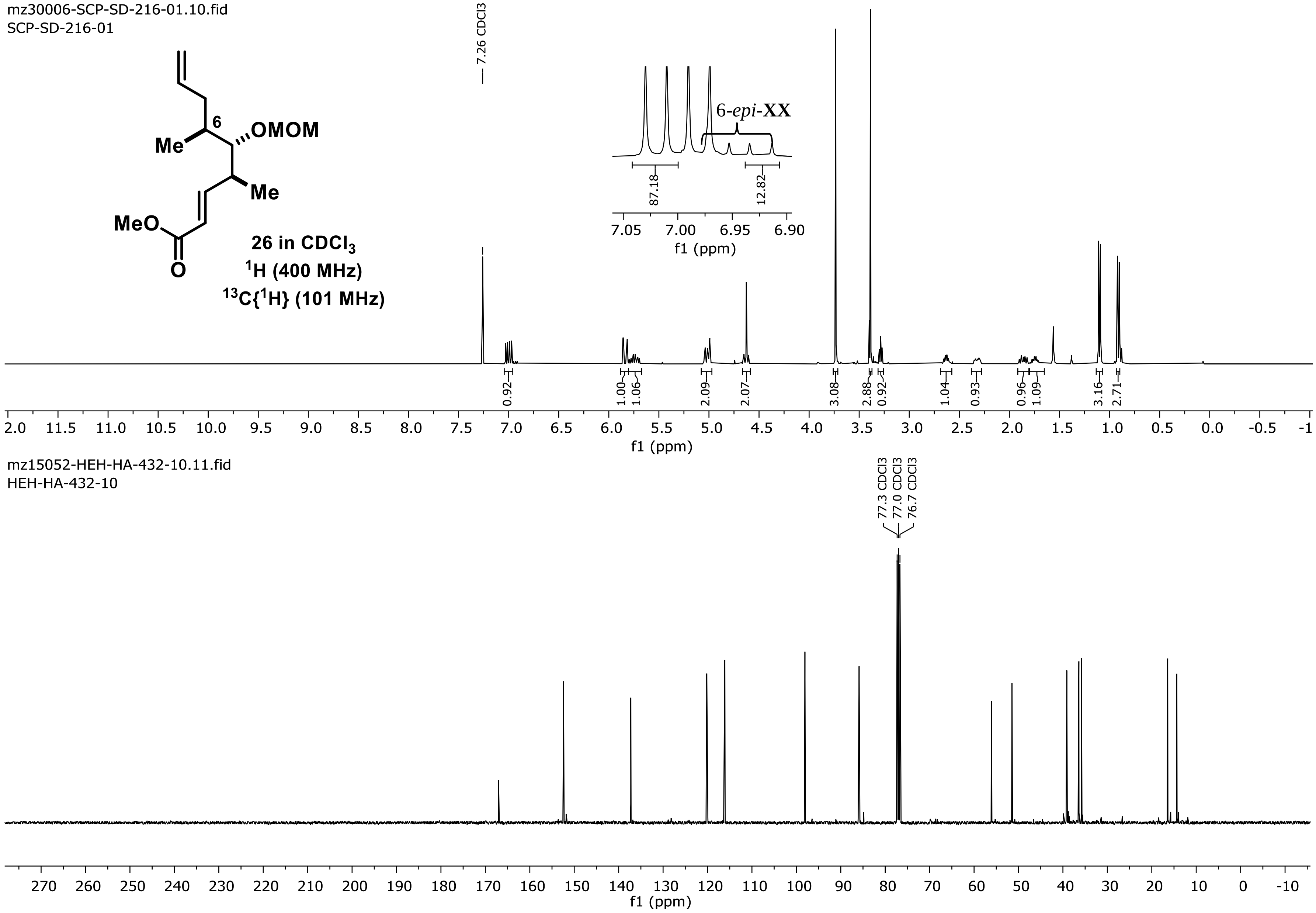
mz30006-SCP-SD-216-01.10.fid
SCP-SD-216-01
7.26 CDCl3
6
OMOM
Me
Me
MeO
O
26 in CDCl3
1H (400 MHz)
13C{1H} (101 MHz)
6-epi-XX
87.18
12.82
7.05 7.00 6.95 6.90
f1 (ppm)
0.92 1.00 1.06 2.09 2.07 3.08 2.88 0.92 1.04 0.93 0.96 1.09 3.16 2.71
2.0 11.5 11.0 10.5 10.0 9.5 9.0 8.5 8.0 7.5 7.0 6.5 6.0 5.5 5.0 4.5 4.0 3.5 3.0 2.5 2.0 1.5 1.0 0.5 0.0 -0.5 -1
f1 (ppm)
mz15052-HEH-HA-432-10.11.fid
HEH-HA-432-10
77.3 CDCl3
77.0 CDCl3
76.7 CDCl3
270 260 250 240 230 220 210 200 190 180 170 160 150 140 130 120 110 100 90 80 70 60 50 40 30 20 10 0 -10
f1 (ppm)

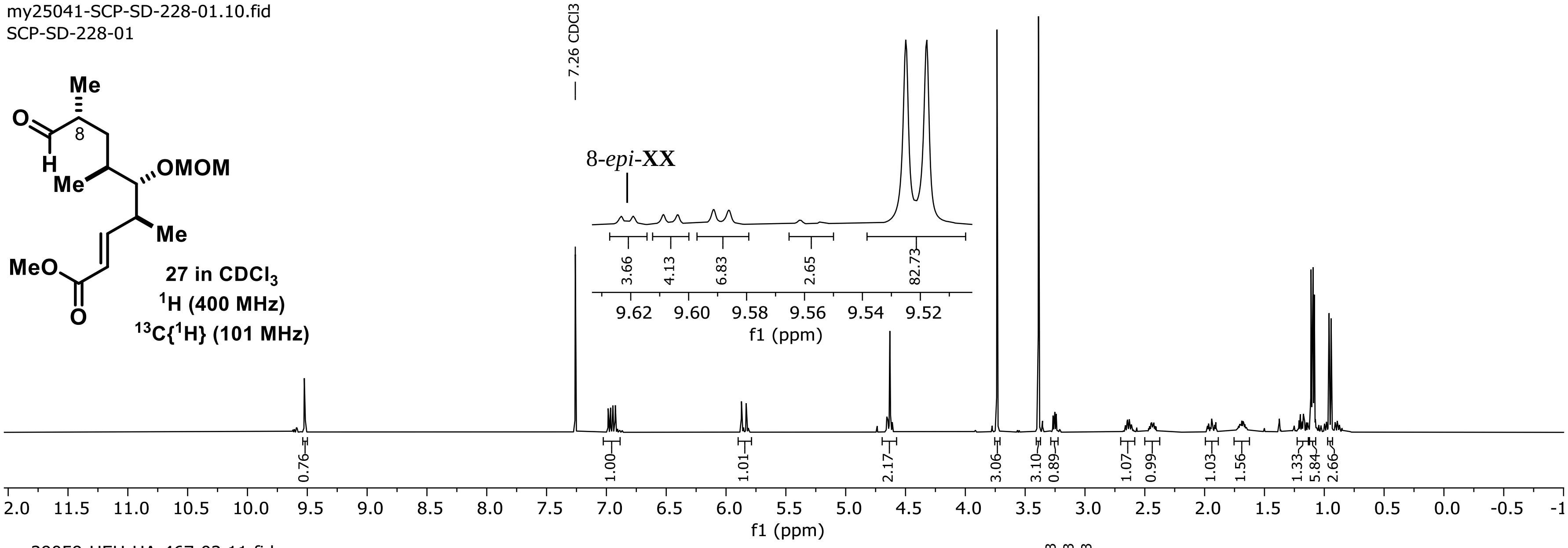
my25041-SCP-SD-228-01.10.fid
SCP-SD-228-01
Me
O
8
H
Me
OMOM
Me
MeO
O
27 in CDCl3
1H (400 MHz)
13C{1H} (101 MHz)
7.26 CDCl3
8-epi-XX
3.66
4.13
6.83
2.65
82.73
9.62 9.60 9.58 9.56 9.54 9.52
f1 (ppm)
0.76
1.00
1.01
2.17
3.06
3.10
0.89
1.07
0.99
1.03
1.56
1.33
5.84
2.66
2.0 11.5 11.0 10.5 10.0 9.5 9.0 8.5 8.0 7.5 7.0 6.5 6.0 5.5 5.0 4.5 4.0 3.5 3.0 2.5 2.0 1.5 1.0 0.5 0.0 -0.5 -1
f1 (ppm)

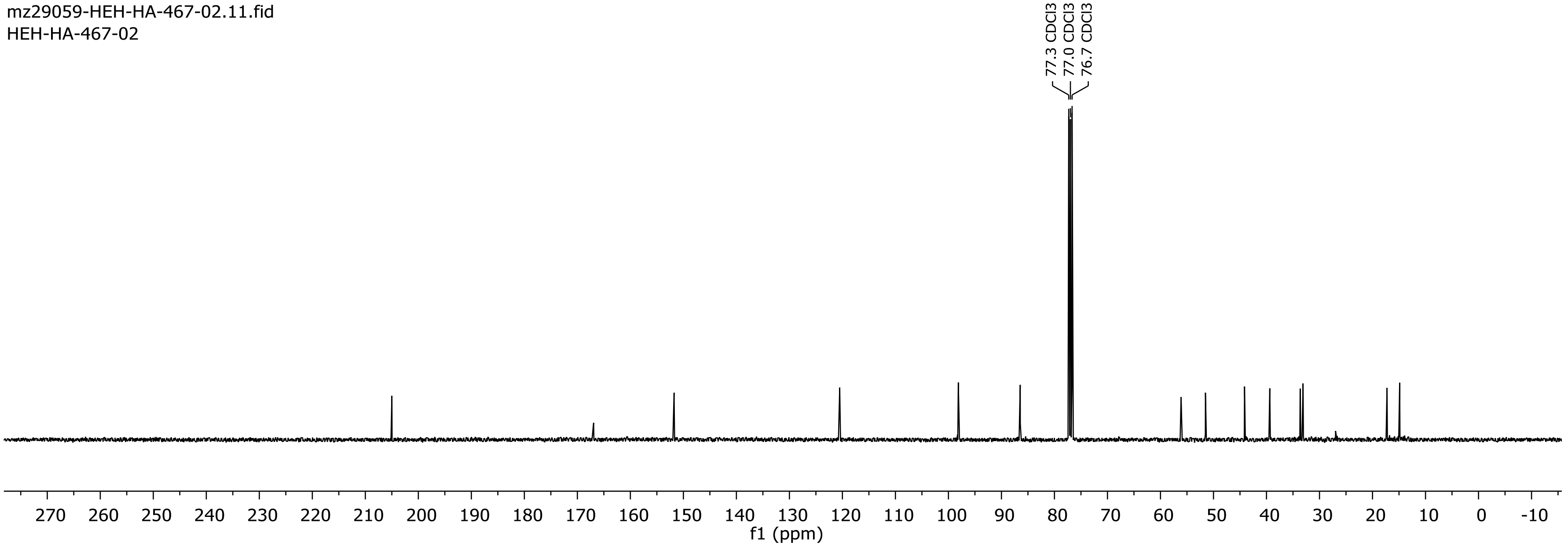
mz29059-HEH-HA-467-02.11.fid
HEH-HA-467-02
77.3 CDCl3
77.0 CDCl3
76.7 CDCl3
270 260 250 240 230 220 210 200 190 180 170 160 150 140 130 120 110 100 90 80 70 60 50 40 30 20 10 0 -10
f1 (ppm)

al04052-HEH-HA-474-04.10.fid
HEH-HA-474-04
F4

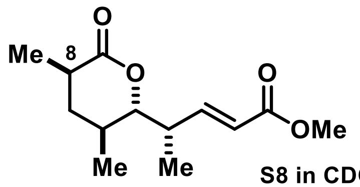

S8 in $CDCl_3$
^{1}H (400 MHz)
$^{13}C\{^{1}H\}$ (101 MHz)

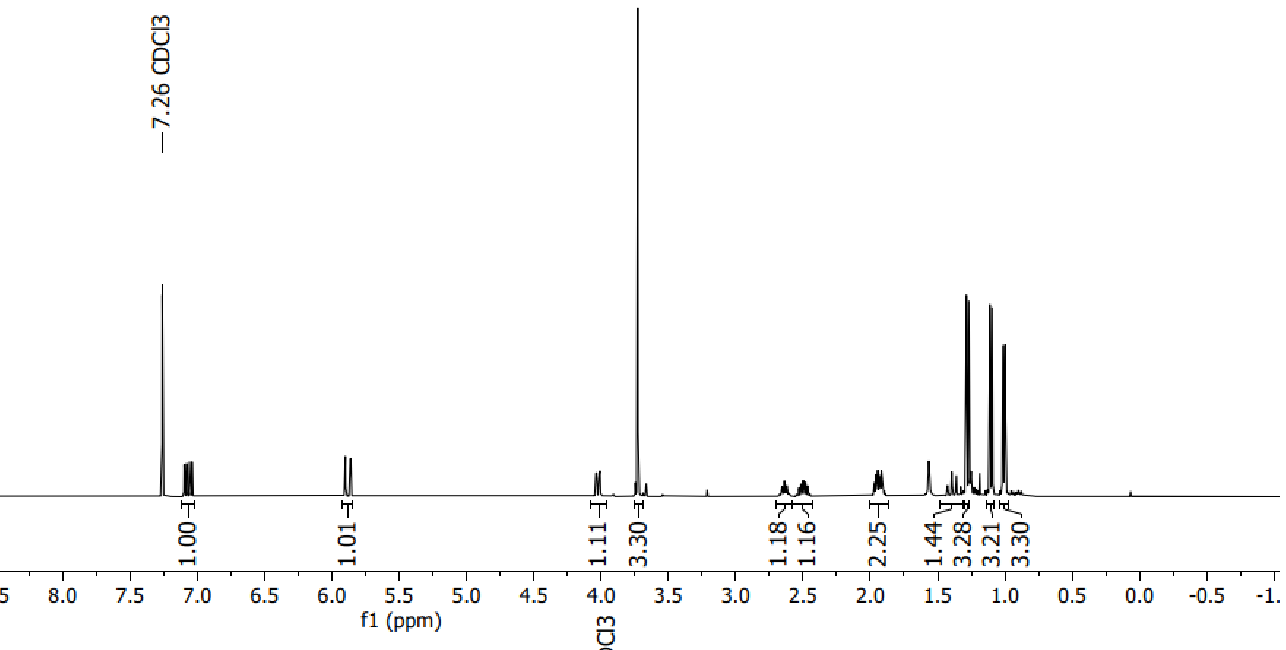

al04052-HEH-HA-474-04.11.fid
HEH-HA-474-04
F4

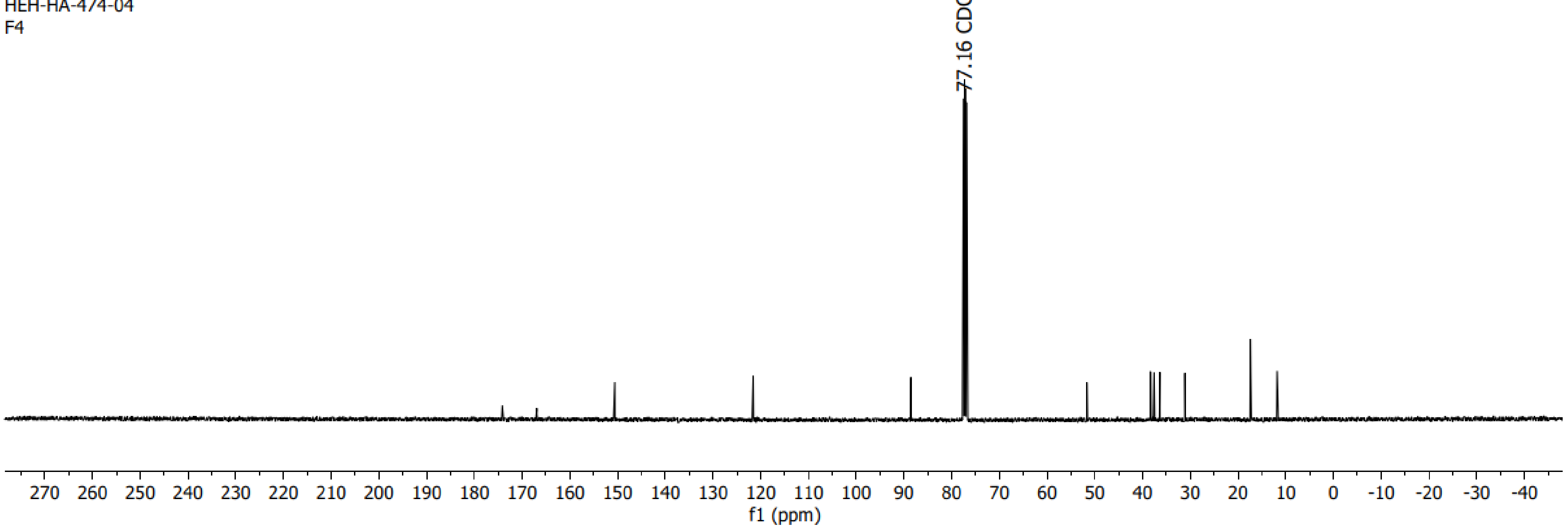

jy10004-HEH-HA-247-02.10.fid
HEH-HA-247-02

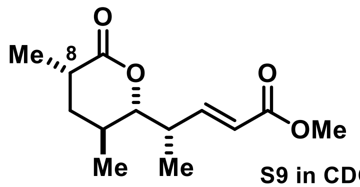

S9 in $CDCl_3$
^{1}H (400 MHz)
$^{13}C\{^{1}H\}$ (101 MHz)

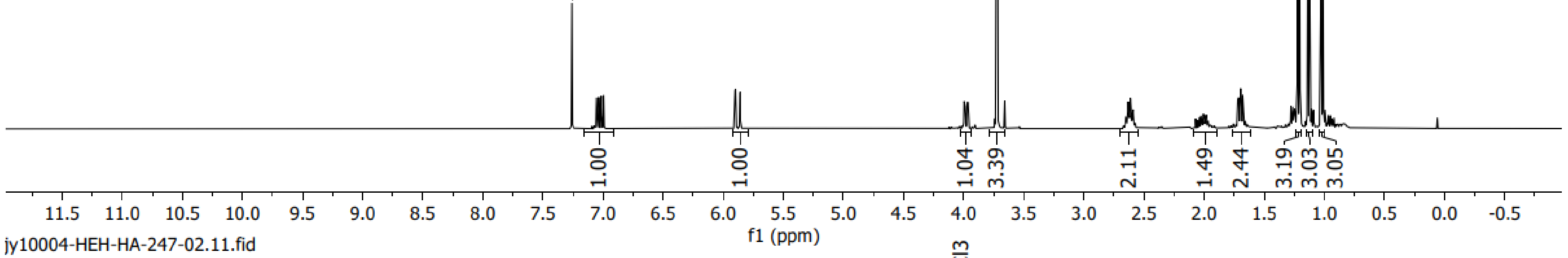

jy10004-HEH-HA-247-02.11.fid
HEH-HA-247-02

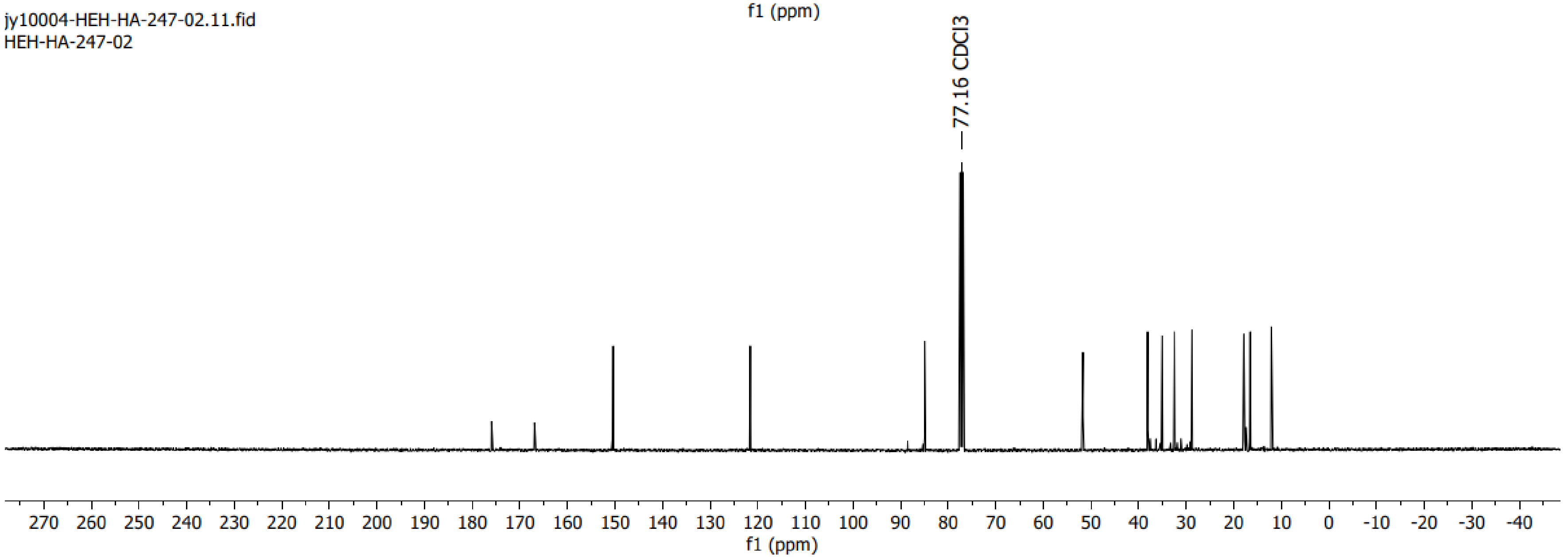

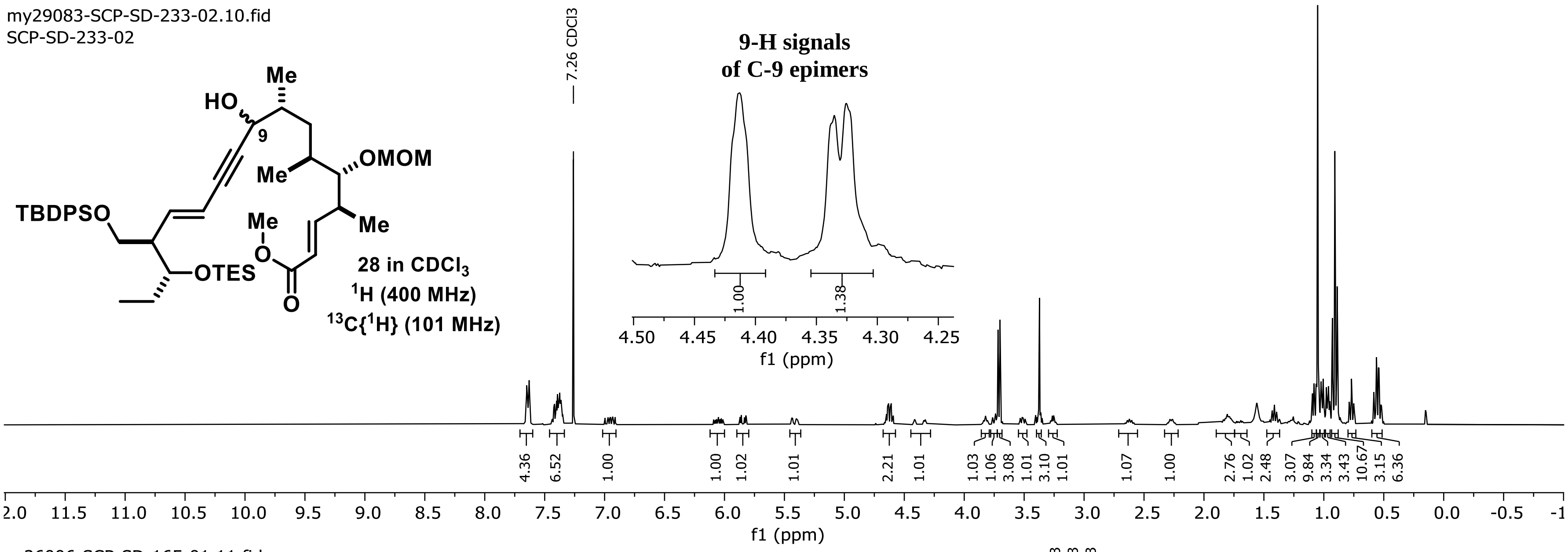

my29083-SCP-SD-233-02.10.fid
SCP-SD-233-02
7.26 CDCl3
9-H signals
of C-9 epimers
HO
9
Me
OMOM
TBDPSO
OTES
Me
O
28 in CDCl3
1H (400 MHz)
13C{1H} (101 MHz)
1.00
1.38
f1 (ppm)
4.36
6.52
1.00
1.00
1.02
1.01
2.21
1.01
1.03
1.06
3.08
1.01
3.10
1.01
1.07
1.00
2.76
1.02
2.48
3.07
9.84
3.34
3.43
10.67
3.15
6.36
f1 (ppm)

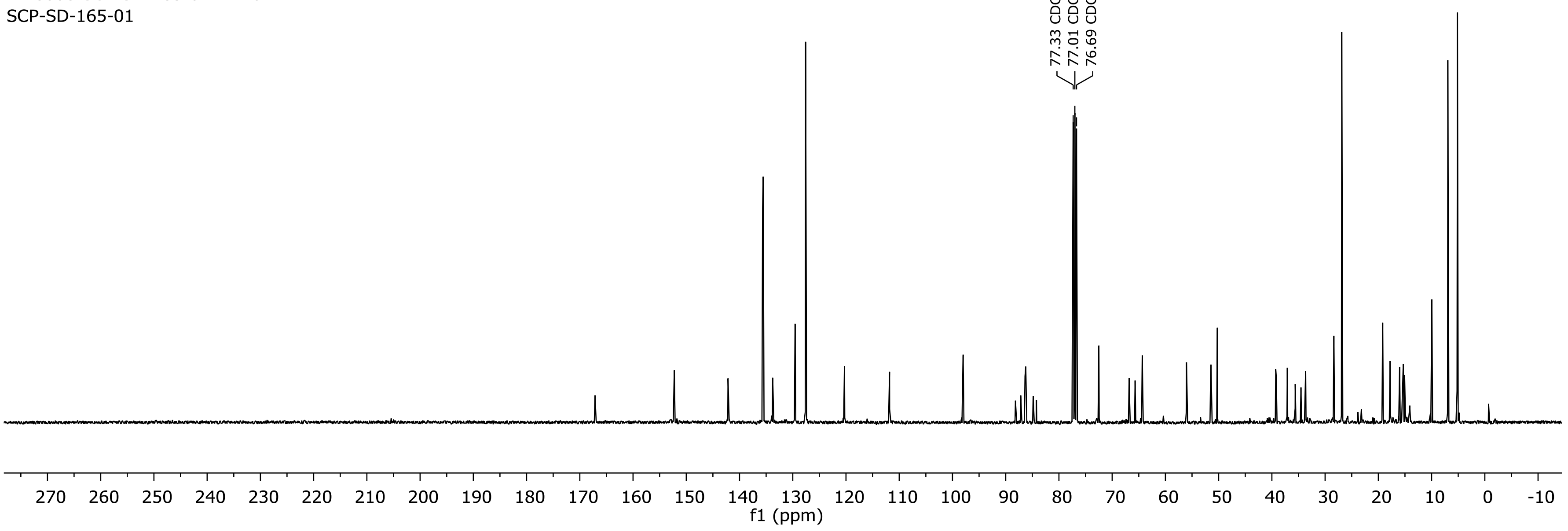

nr26006-SCP-SD-165-01.11.fid
SCP-SD-165-01
77.33 CDCl3
77.01 CDCl3
76.69 CDCl3
f1 (ppm)

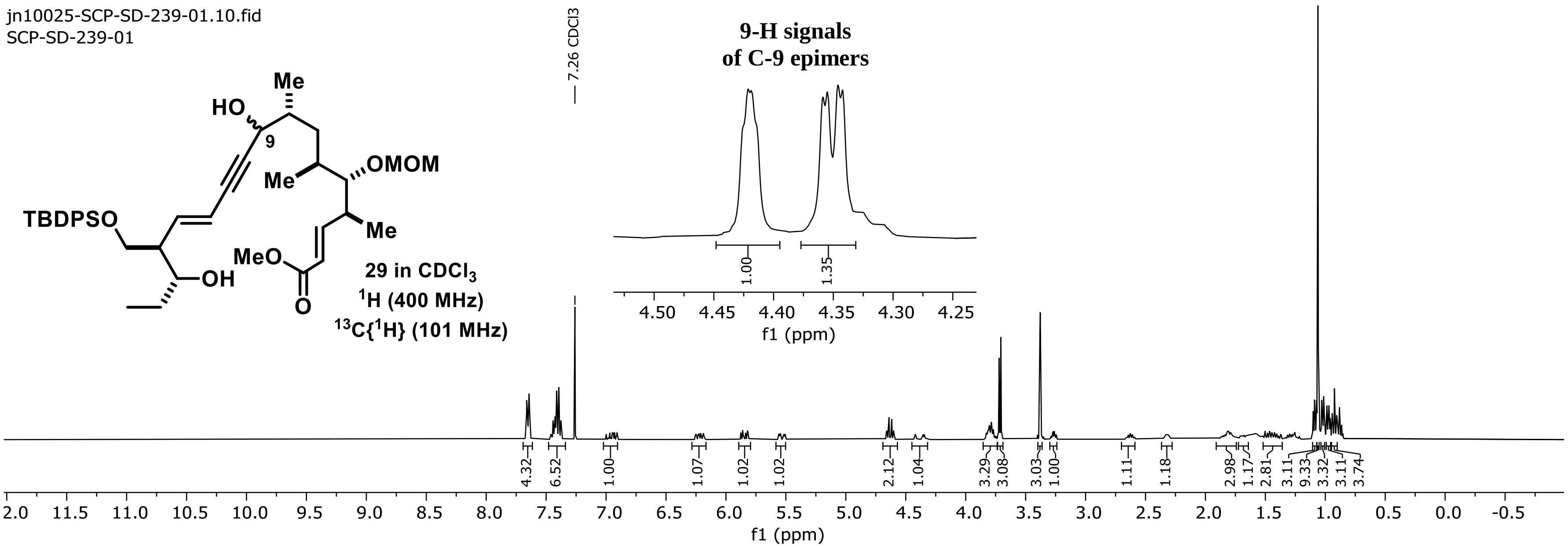
jn10025-SCP-SD-239-01.10.fid
SCP-SD-239-01
29 in CDCl3
1H (400 MHz)
13C{1H} (101 MHz)
7.26 CDCl3
9-H signals
of C-9 epimers
1.00
1.35
f1 (ppm)
4.32
6.52
1.00
1.07
1.02
1.02
2.12
1.04
3.29
3.08
3.03
1.00
1.11
1.18
2.98
1.17
2.81
3.11
9.33
3.32
3.11
3.74
f1 (ppm)

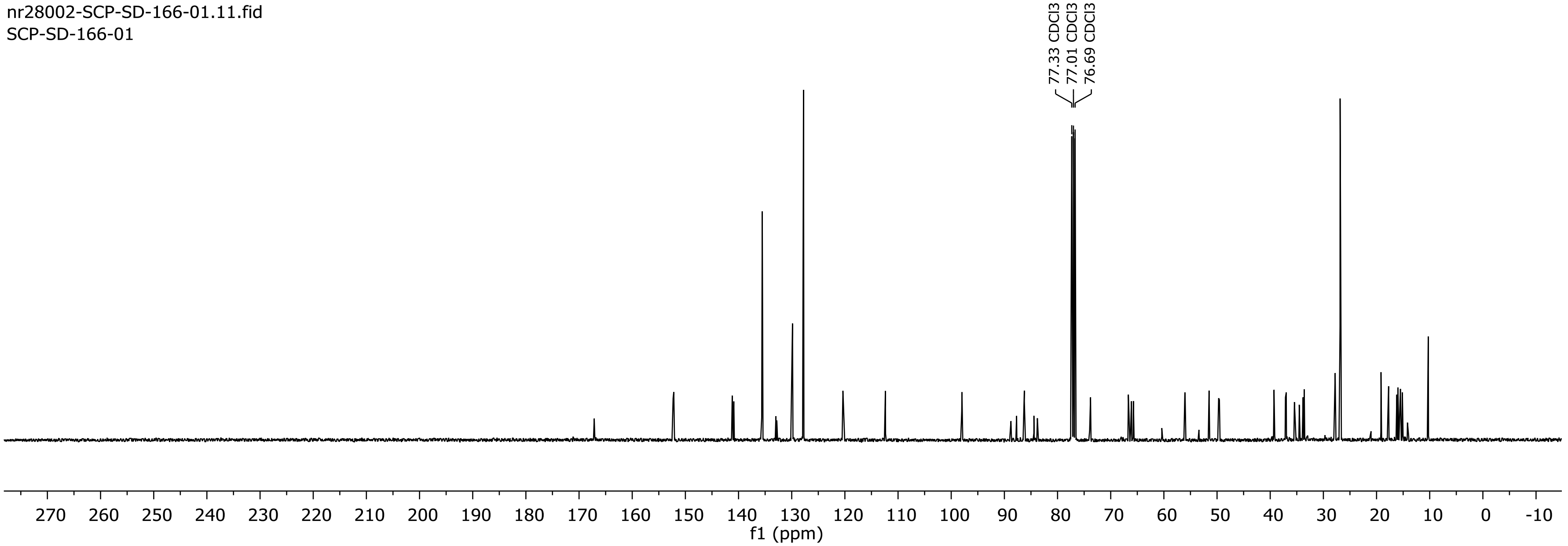
nr28002-SCP-SD-166-01.11.fid
SCP-SD-166-01
77.33 CDCl3
77.01 CDCl3
76.69 CDCl3
f1 (ppm)

jr21059-SCP-SD-185-01.10.fid
SCP-SD-185-01

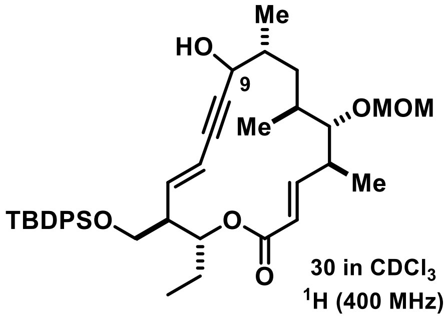

30 in CDCl$_3$
^{1}H (400 MHz)
^{13}C{1H} (101 MHz)

(single C-9 epimer)

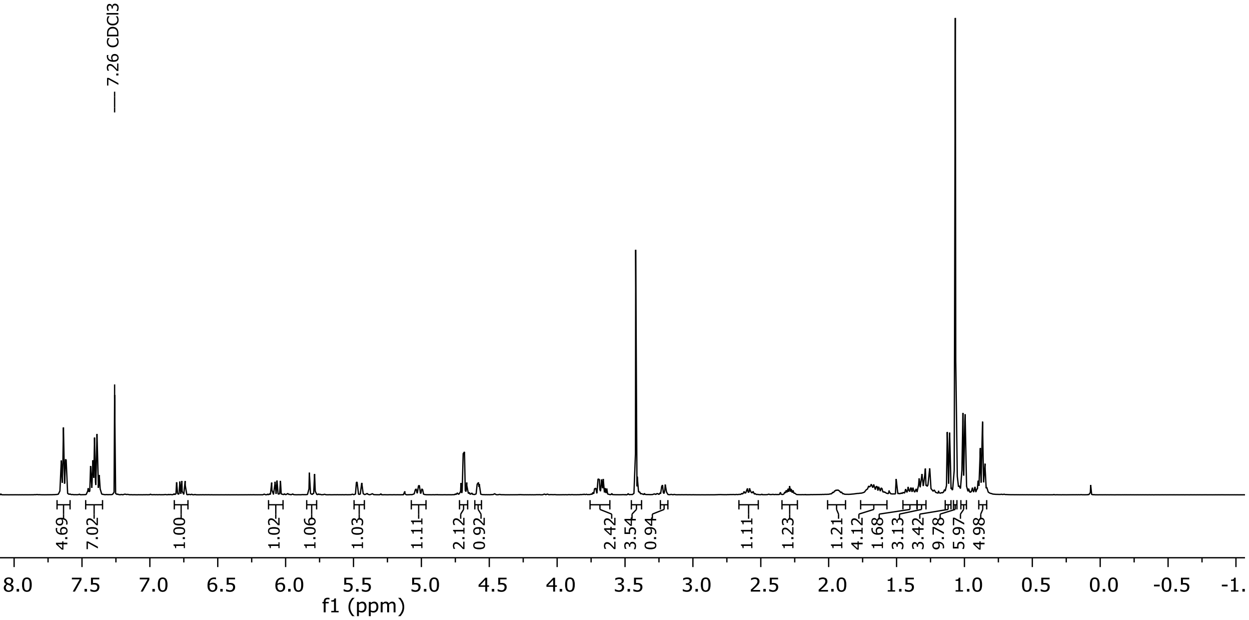

jr21059-SCP-SD-185-01.12.fid
SCP-SD-185-01

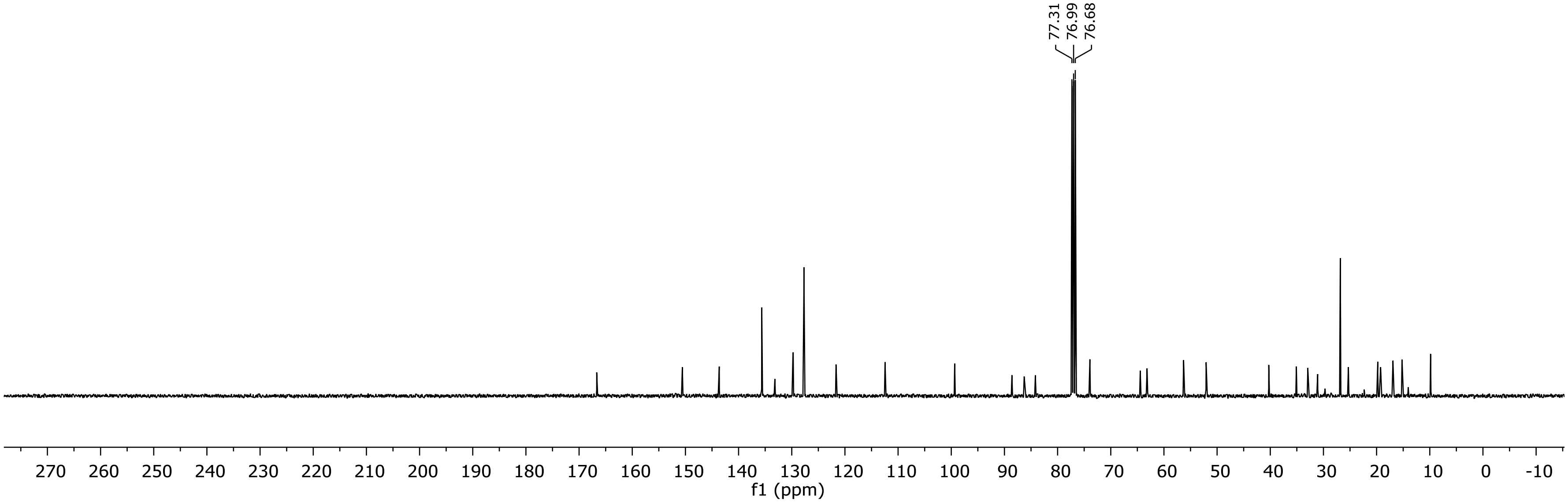

jy01010-SCP-SD-243-01.10.fid
SCP-SD-243-01

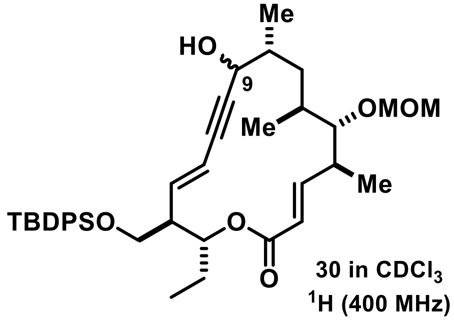

(mixture of C-9 epimers)

30 in $CDCl_3$
^1H (400 MHz)
$^{13}C\{1H\}$ (101 MHz)

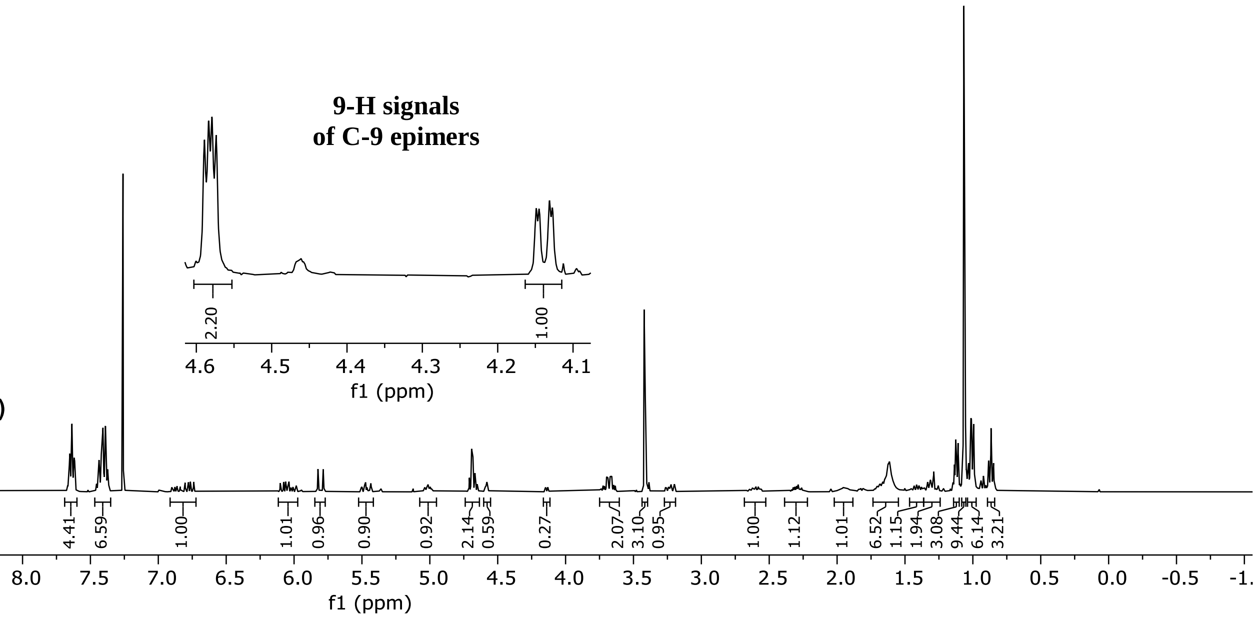

fr20013-SCP-SD-185-05.11.fid
SCP-SD-185-05

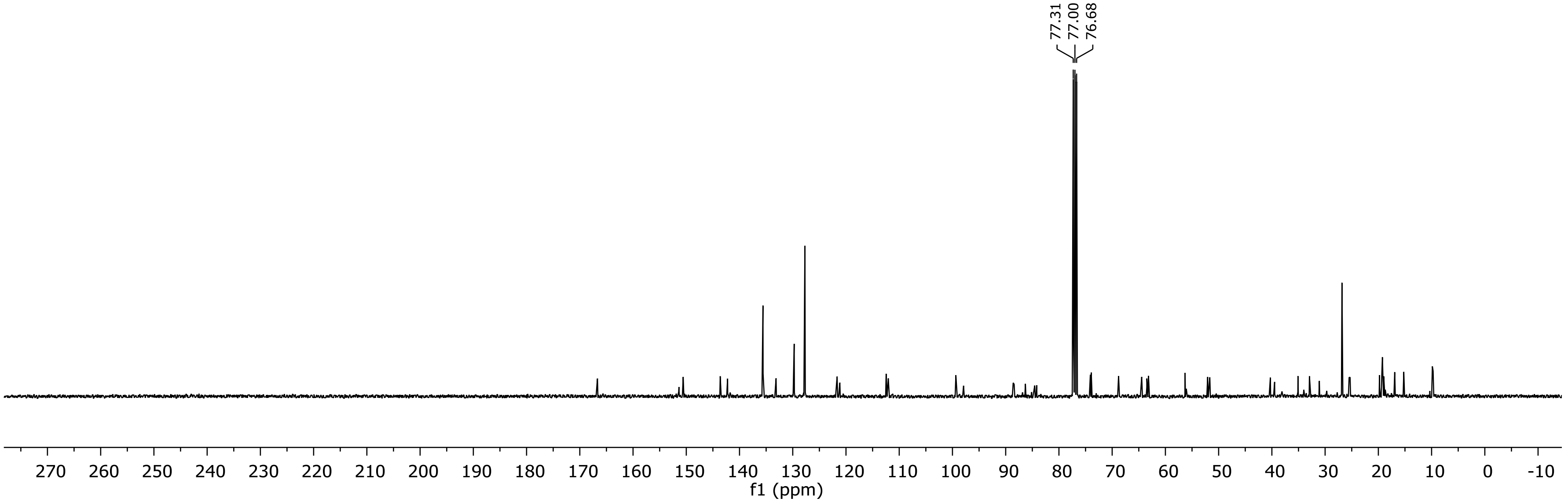

jy01055-SCP-SD-245-01.10.fid
SCP-SD-245-01

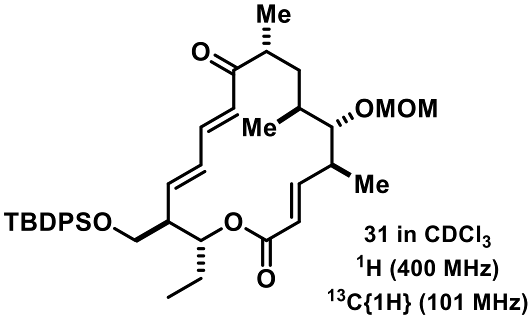

31 in CDCl$_3$
^{1}H (400 MHz)
^{13}C{1H} (101 MHz)

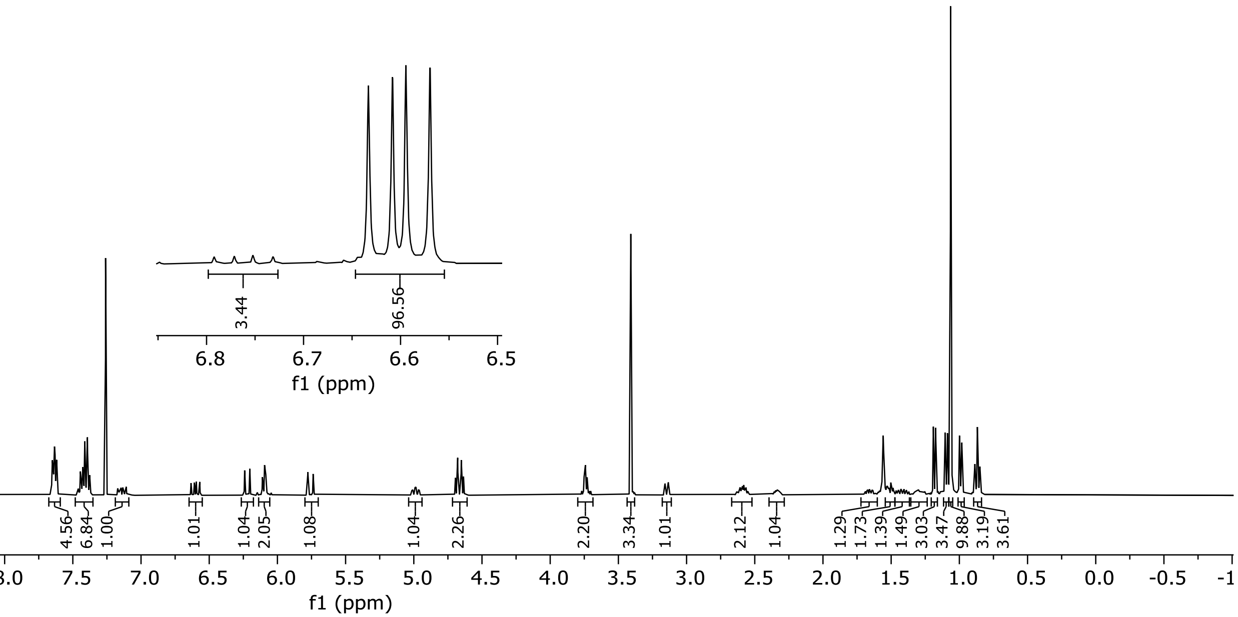

fr24038-SCP-SD-208-01.11.fid
SCP-SD-208-01

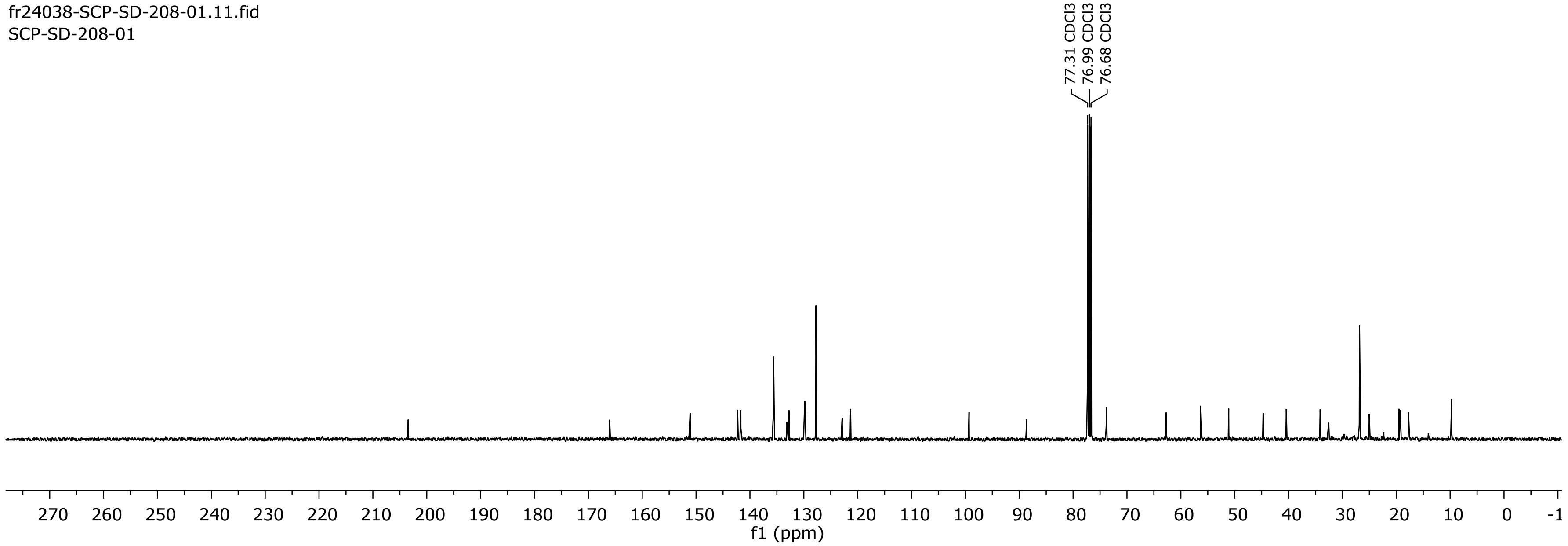

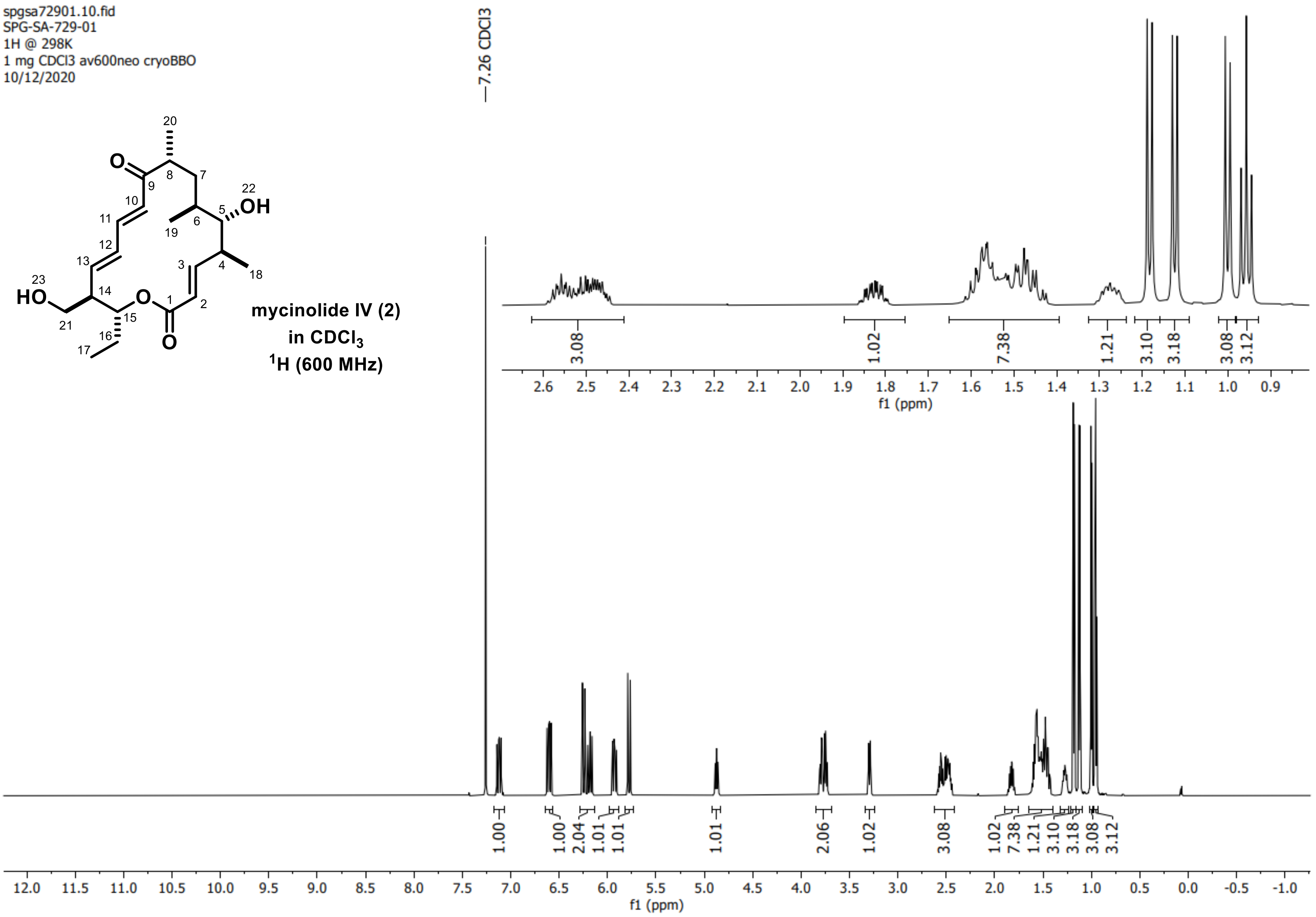
spgsa72901.10.fid
SPG-SA-729-01
1H @ 298K
1 mg CDCl3 av600neo cryoBBO
10/12/2020
mycinolide IV (2)
in CDCl$_3$
^{1}H (600 MHz)
—7.26 CDCl3
f1 (ppm)
f1 (ppm)

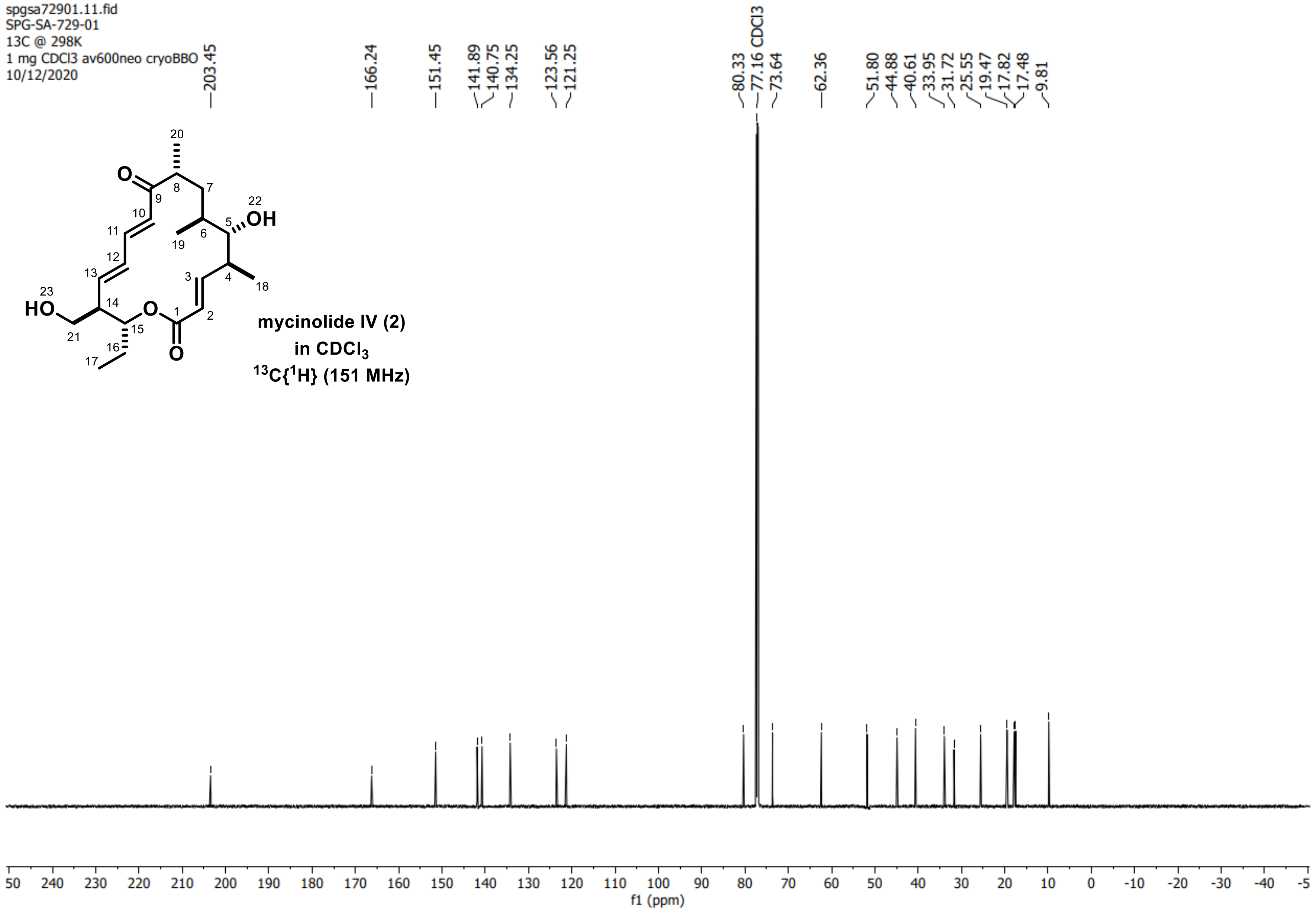
spgsa72901.11.fid
SPG-SA-729-01
13C @ 298K
1 mg CDCl3 av600neo cryoBBO
10/12/2020
203.45
166.24
151.45
141.89
140.75
134.25
123.56
121.25
80.33
77.16 CDCl3
73.64
62.36
51.80
44.88
40.61
33.95
31.72
25.55
19.47
17.82
17.48
9.81
mycinolide IV (2)
in CDCl3
13C{1H} (151 MHz)
f1 (ppm)

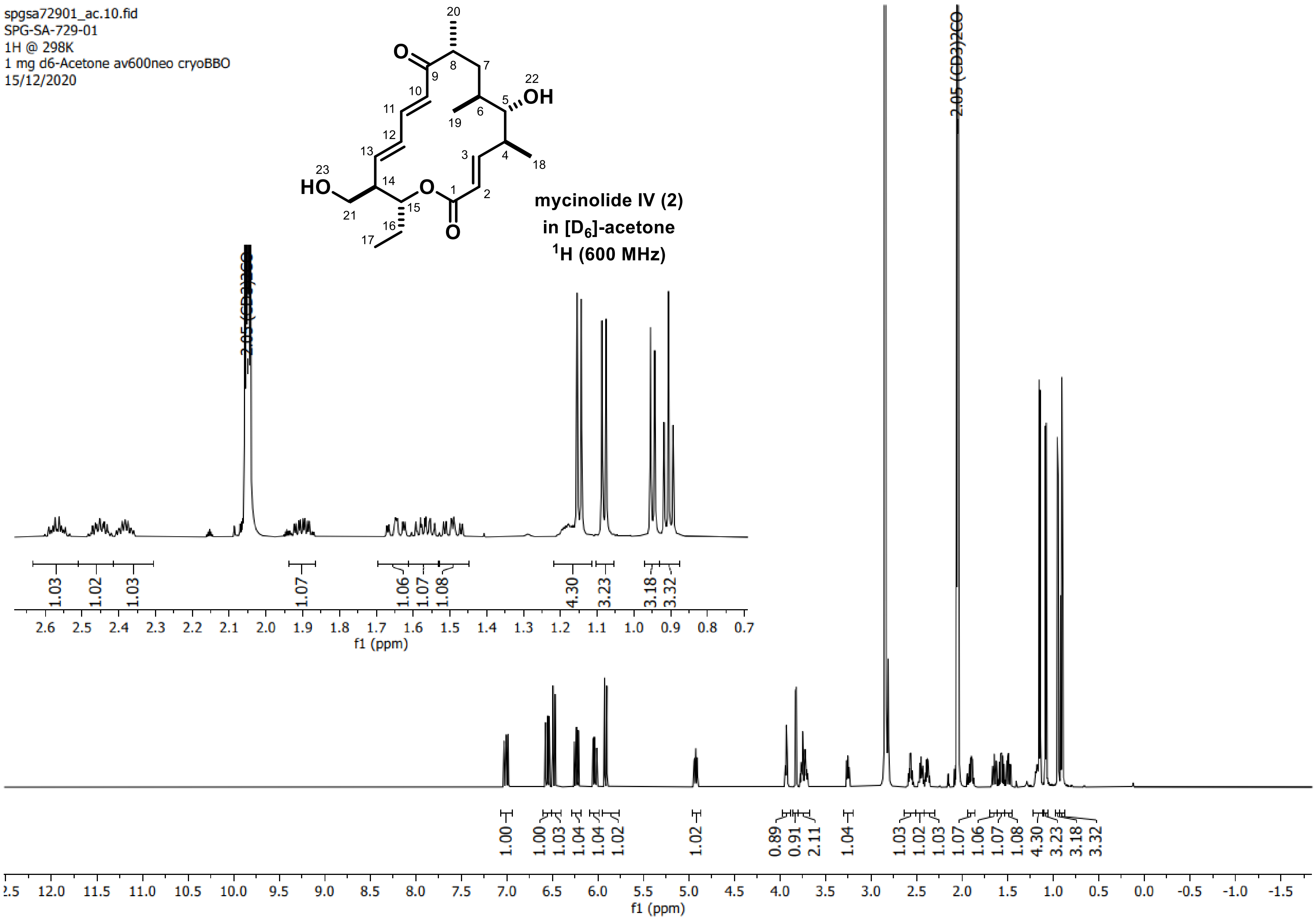
spgsa72901_ac.10.fid
SPG-SA-729-01
1H @ 298K
1 mg d6-Acetone av600neo cryoBBO
15/12/2020
mycinolide IV (2)
in [D6]-acetone
1H (600 MHz)
HO
OH
O
2.05 (CD3)2CO
f1 (ppm)

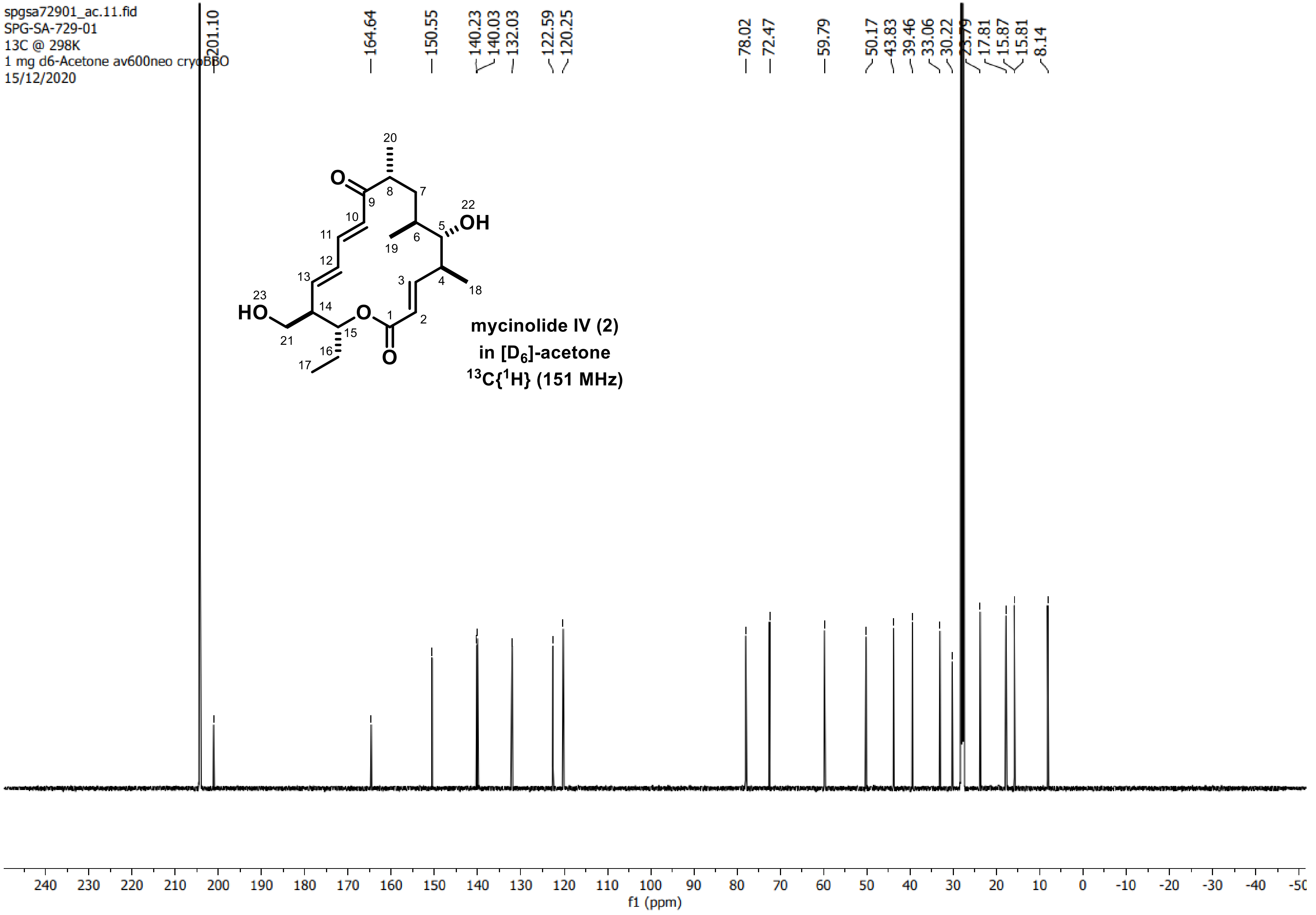

spgsa72901_ac.11.fid
SPG-SA-729-01
13C @ 298K
1 mg d6-Acetone av600neo cryoBBO
15/12/2020
201.10
164.64
150.55
140.23
140.03
132.03
122.59
120.25
78.02
72.47
59.79
50.17
43.83
39.46
33.06
30.22
17.81
15.87
15.81
8.14
mycinolide IV (2)
in [D6]-acetone
13C{1H} (151 MHz)
f1 (ppm)

References

[1] B. Bazán-Tejeda, G. Bluet, G. Broustal, J. Campagne, *Chem. Eur. J.* **2006**, *12*, 8358 – 8366.

[2] R. Okawara, M. Wada, *J. Organomet. Chem*. **1963**, *1*, 81-88.

[3] J. Beckmann, D. Dakternieks, F. Sheen Kuan, E. R. T. Tiekink, *J. Organomet. Chem.* **2002**, *659*, 73–83.

[4] L. Iu, J. A. Fuentes, M. E. Janka, K. J. Fontenot, M. L. Clarke, *Angew. Chem. Int. Ed.* **2019**, *58*, 2120 – 2124; F. Guillen, M. Rivard, M. Toffano, J.-Y. Legros, J.-C. Daran, J.-C. Fiaud, *Tetrahedron* **2002**, *58*, 5895 – 5904.

[5] L. Ferrié, J. Fenneteau, B. Figadère, *Org. Lett.* **2018**, *20*, 3192 – 3196.

[6] R. Baker, J. C. Head, C. J. Swain, *J. Chem. Soc. Perkin Trans. 1* **1988**, 85–97.

[7] A. H. Hoveyda, F. Meng, F. Haeffner, *J. Am. Chem. Soc.* **2014**, *136*, 11304–11307.

[8] C.-X. Wang, R. Ding, S.-T. Jiang, J.-S. Tang, D. Hu, G.-D. Chen, F. Lin, K. Hong, X.-S. Yao, H. Gao, *J. Nat. Prod*. **2016**, *79*, 2446-2454.

[9] G. Späth, A. Fürstner, accompanying paper in this issue.

[10] M. Honda, T. Katsuki, M. Yamaguchi, *Tetrahedron Lett*. **1984**, *25*, 3857–3860.

[11] K. Suzuki, T. Matsumoto, K. Tomooka, K. Matsumoto, G.-I. Tsuchihashi, *Chem. Lett.* **1987**, 113–116.

[12] M. Hayashi, M. Ohno, K. Kinoshita, S. Satoi, M. Suzuki, K.-I. Harada, *J. Antibiotics* **1981**, *34*, 346–349.

[13] M. Hayashi, K. Kinoshita, Y. Sudate, S. Satoi, H. Sakakibara, K.-I. Harada, M. Suzuki, *J. Antibiotics* **1983**, *36*, 175–178.
